# TFCP2L1 suppresses breast cancer progression by promoting ferroptosis through direct and PI3K/AKT-mediated inhibition of GPX4

**DOI:** 10.1038/s41420-026-03231-7

**Published:** 2026-06-26

**Authors:** Xiaoxiao Wang, Wei Lu, Yandi Cui, Keqiang He, Yuanzhi Bie, Shou-Dong Ye, Xiangfen Li

**Affiliations:** 1 Department of Anesthesiology, The First Affiliated Hospital of USTC, Division of Life Sciences and Medicine, University of Science and Technology of China, Hefei, 230001 China; 2 Center for Stem Cell and Translational Medicine, School of Life Sciences and Medical Engineering, Anhui University, Hefei, 230601 China; 3 Department of Genetics and Cell Biology, College of Life Sciences, Nankai University, Tianjin, 300071 China; 4 Department of Otolaryngology-Head and Neck Surgery, The First Affiliated Hospital of USTC, Division of Life Sciences and Medicine, University of Science and Technology of China, Hefei, 230001 China; 5 Shanghai Key Laboratory of Regulatory Biology, Institute of Biomedical Sciences and School of Life Sciences, East China Normal University, Shanghai, 200241 China

**Keywords:** Cancer, Cell signalling, Diagnostic markers, Breast cancer

## Abstract

Breast cancer is the most prevalent type of malignant tumor among women. Here, we identified transcription factor CP2-like 1 (TFCP2L1) as a suppressor of breast cancer cells. Patients with breast cancer have poorer prognoses when *TFCP2L1* is expressed at low levels. Consistently, the expression of *TFCP2L1* is greater in normal breast tissues and cells than in breast cancer tissues and cell lines. Gain- and loss-of-function experiments and xenograft tumor assays revealed that overexpression of *TFCP2L1* significantly inhibited the proliferation and migration of breast cancer cells. Conversely, the knockdown of *TFCP2L1* promoted the growth and migration of breast cancer cells. Mechanistically, TFCP2L1 suppresses breast cancer progression by inhibiting the expression of glutathione peroxidase 4 (*GPX4*), a key positive regulator of ferroptosis. Therefore, the addition of a ferroptosis inhibitor, Ferrostatin-1, can mediate the function of TFCP2L1 in breast cancer cells. On the other hand, high-throughput transcriptome sequencing revealed that *TFCP2L1* negatively regulates the activity of the PI3K/AKT signaling pathway. The administration of SC79, an AKT activator, partially reversed the negative effects of TFCP2L1 on *GPX4* transcription. Together, these findings indicate that TFCP2L1 functions partially by directly and indirectly regulating *GPX4*-mediated ferroptosis and may serve as a potential therapeutic target for breast cancer.

## Introduction

Breast cancer is a highly aggressive disease that poses a significant threat to women’s lives [1]. Over the past decade, its incidence and mortality have ranked highest among all malignant tumors affecting women [1]. There are substantial differences among breast cancer patients, with classification into Luminal A and B breast cancer, Human epidermal growth factor receptor 2 (HER-2) enriched breast cancer, and triple-negative breast cancer (TNBC) on the basis of the levels of estrogen receptor (ER), progesterone receptor (PR), and HER-2 [2]. Treatment modalities for breast cancer include surgical procedures, radiotherapy, chemotherapy, endocrine therapy, and targeted therapy [3]. Recent advancements in these treatments have led to improved five-year survival rates among breast cancer patients. Approximately 95% of patients diagnosed with stage I disease achieve at least five-year survival, which contrasts sharply with the survival rate of only 28% for those diagnosed with stage IV disease [4]. This highlights the significant challenge of overcoming distant metastasis to enhance the outcomes of breast cancer treatments. Protein arginine methyltransferase 6 (PRMT6) methylation of signal transducer and activator of transcription 3 (STAT3) has been reported to promote tumor metastasis in breast cancer [5]. Additionally, enhancer of zeste 2 polycomb repressive complex 2 subunit (EZH2) activates transforming growth factor beta (TGFβ) signaling to facilitate breast cancer bone metastasis through integrin β1-FAK activation [6], whereas kinesin family member 17 (KIF17) preserves the epithelial phenotype of cancer cells and inhibits tumor metastasis [7]. These findings present potential avenues for exploring novel treatment methods for metastatic breast cancer. Recently, researchers have highlighted the role of ferroptosis in breast cancer metastasis [8–10], suggesting that ferroptosis is a promising strategy for treatment. However, despite these advancements, the overall survival of breast cancer patients remains inadequate, underscoring the need for a deeper understanding of the complex mechanisms underlying breast cancer metastasis and the development of novel therapeutic targets.

Transcription factors (TFs) and their specific interactions with target genes are crucial for the activation of gene expression programs that determine cell fate and behavior from early embryonic development to tissue homeostasis in adulthood [11]. A subset of TFs, such as POU class 5 homeobox 1 (OCT4) and SRY-box transcription factor 2 (SOX2), which are normally expressed in pluripotent stem cells, become aberrantly activated in several tumors, including breast cancer [12–14]. In a recent machine learning study, researchers analyzed 12,000 samples across 33 tumor types from The Cancer Genome Atlas (TCGA). These results revealed that features associated with stemness are characterized by an enrichment of pluripotency-associated TFs and are linked to oncogenic dedifferentiation and tumor metastasis [15]. The transcription factor CP2-like 1 (TFCP2L1) belongs to the TFCP2/Grainyhead family, is a member of the TFCP2/TFCP2L1/UBP1 subfamily, and has been identified as a pluripotency-associated transcription factor [16]. TFCP2L1 serves as a critical downstream target of the LIF/Stat3 and Wnt/β-catenin pathways and is crucial for maintaining the self-renewal of mouse embryonic stem cells (ESCs) [16–18]. The involvement of TFCP2L1 in ESC self-renewal suggests that it may regulate cancer occurrence and development [19]. TFCP2L1 plays various roles in the occurrence and progression of clear cell renal cancer [20], thyroid cancer [21], bladder cancer [22], melanoma [23], and hepatocellular carcinoma [24]. Notably, its role in breast cancer remains unclear. Therefore, understanding the expression pattern and function of TFCP2L1 in breast cancer, as well as its regulatory mechanisms, is highly interesting.

Our study revealed that *TFCP2L1* deficiency is associated with an unfavorable prognosis and advanced clinical stage in breast cancer patients. The upregulation of *TFCP2L1* was shown to inhibit the activity of the Phosphoinositide 3-Kinase/Protein Kinase B (PI3K/AKT) signaling pathway while inducing ferroptosis by decreasing the transcription of glutathione peroxidase 4 (GPX4) in breast cancer cells. These findings provide novel insights into the functional significance of TFCP2L1 during the progression of breast cancer.

## Results

### Low levels of *TFCP2L1* are associated with poor prognosis in breast cancer patients

**Fig. 1:**
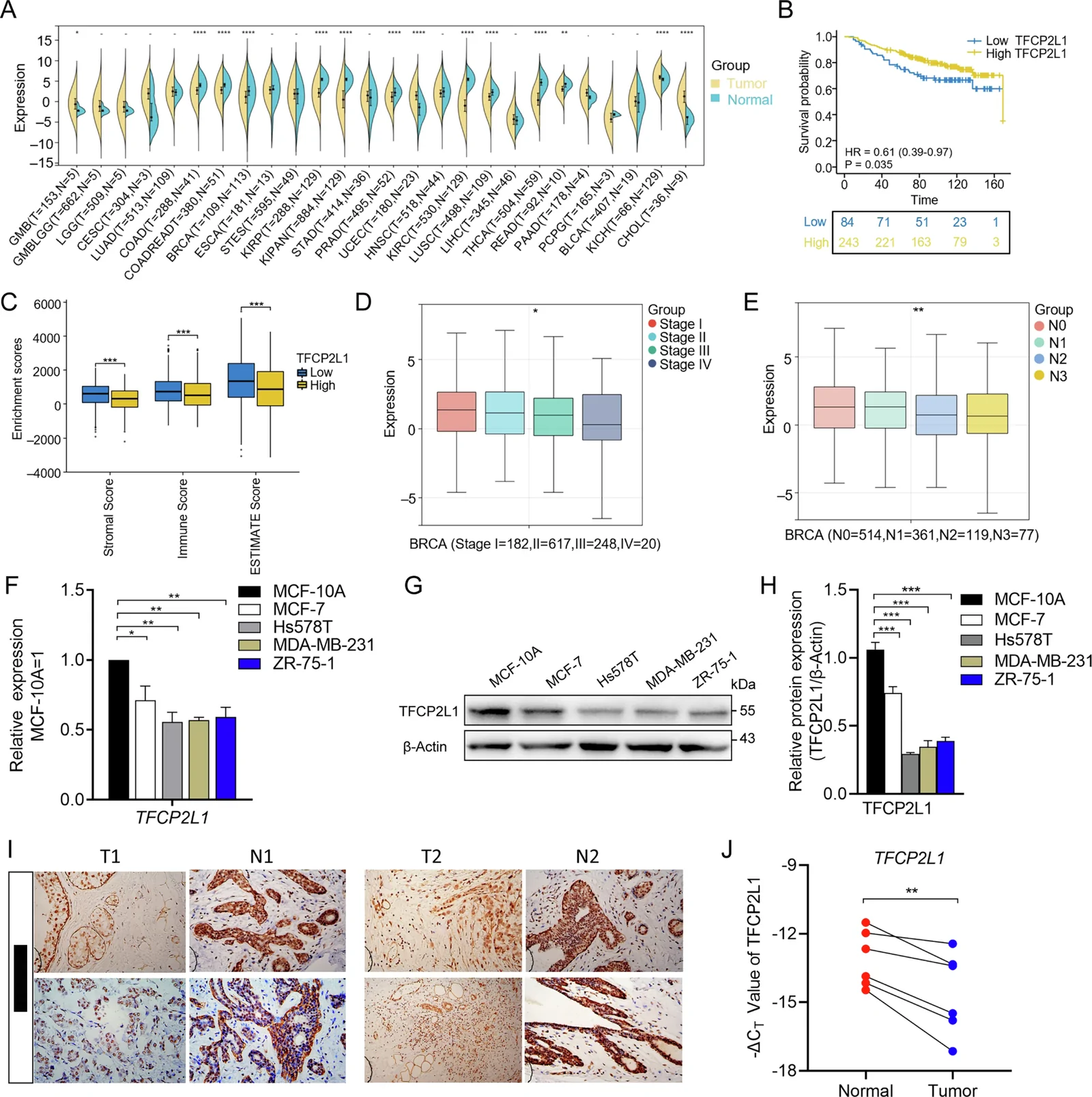
The expression of *TFCP2L1* is low in breast cancer cells and tissues. **A** mRNA level of *TFCP2L1* in different cancer tissues from the TCGA database compared with noncancerous tissues. **B** Kaplan‒Meier survival curves illustrating the survival analysis of patients with different expression levels of *TFCP2L1* in the breast cancer subtype cohort of the TCGA database. **C** Boxplot comparisons of immune infiltration groups in breast cancer patients with high and low expression of *TFCP2L1*. Data were sourced from the TCGA database. **D,E** Boxplots depicting *TFCP2L1* expression levels in breast cancer patients in the TCGA database categorized by clinical TNM stage **D** and N stage **E**. **F** RT‒qPCR analysis of *TFCP2L1* mRNA levels in the cell lines of MCF-10A, MCF-7, Hs578T, MDA-MB-231, and ZR-75-1 cells (*n* = 3). **G** Western blot analysis of the protein levels of TFCP2L1 in different breast cell lines. **H** Densitometric analysis of the relative protein levels of TFCP2L1, as shown in Fig. 1G, was performed with ImageJ software. Protein levels were normalized to those of β-Actin (*n* = 3). **I** Immunohistochemical image showing TFCP2L1 staining in breast cancer tissues and paired adjacent normal tissues (magnification of 400×). Two distinct fields of view were captured per sample. T, Tumor; N, Normal. **J** RT-qPCR analysis of the expression of TFCP2L1 in breast cancer tissues and paired adjacent normal tissues (*n* = 6). The data are presented as mean ± SD. Statistical analyses were performed using one-way ANOVA **F**,**H**. *p < 0.05, **p < 0.01, ***p < 0.001.

To gain insight into the expression pattern of TFCP2L1 in human breast cancer, we conducted an analysis based on the TCGA database and observed a significant reduction in *TFCP2L1* expression in breast cancer tissues compared with normal breast tissues (Fig. 1A). Using the Gene Expression Omnibus (GEO) and TCGA databases, we subsequently analyzed the survival curves and immune infiltration scores of breast cancer patients stratified by *TFCP2L1* expression levels. We observed that patients with high *TFCP2L1* expression presented a better prognosis and lower immune infiltration ability (Fig. 1B,C). Furthermore, to explore the correlation of *TFCP2L1* expression with breast cancer metastasis, we analyzed clinicopathologic parameters from the TCGA cohort and examined *TFCP2L1* mRNA levels in relation to metastatic incidence. The results revealed a significant association between downregulated *TFCP2L1* mRNA and advanced tumor node metastasis (TNM) stage (Fig. 1D,E). A panel of breast cancer cell lines, Hs578T, MCF-7, ZR-75-1 and MDA-MB-231, which are characterized by different malignant behaviors, and a normal breast cell line, MCF-10A, were further used to examine *TFCP2L1* levels. Consistently, lower *TFCP2L1* expression was detected in breast cancer cells at the mRNA and protein levels (Fig. 1F–H). Similarly, the protein levels of TFCP2L1 were lower in breast cancer tissues than in adjacent normal tissues (Fig. 1I,J; Fig. S1A). Collectively, these results suggest that high level of *TFCP2L1* may play a negative role in breast cancer metastasis.

### Overexpression of *TFCP2L1* inhibits the progression of breast cancer cells in vitro

**Fig. 2:**
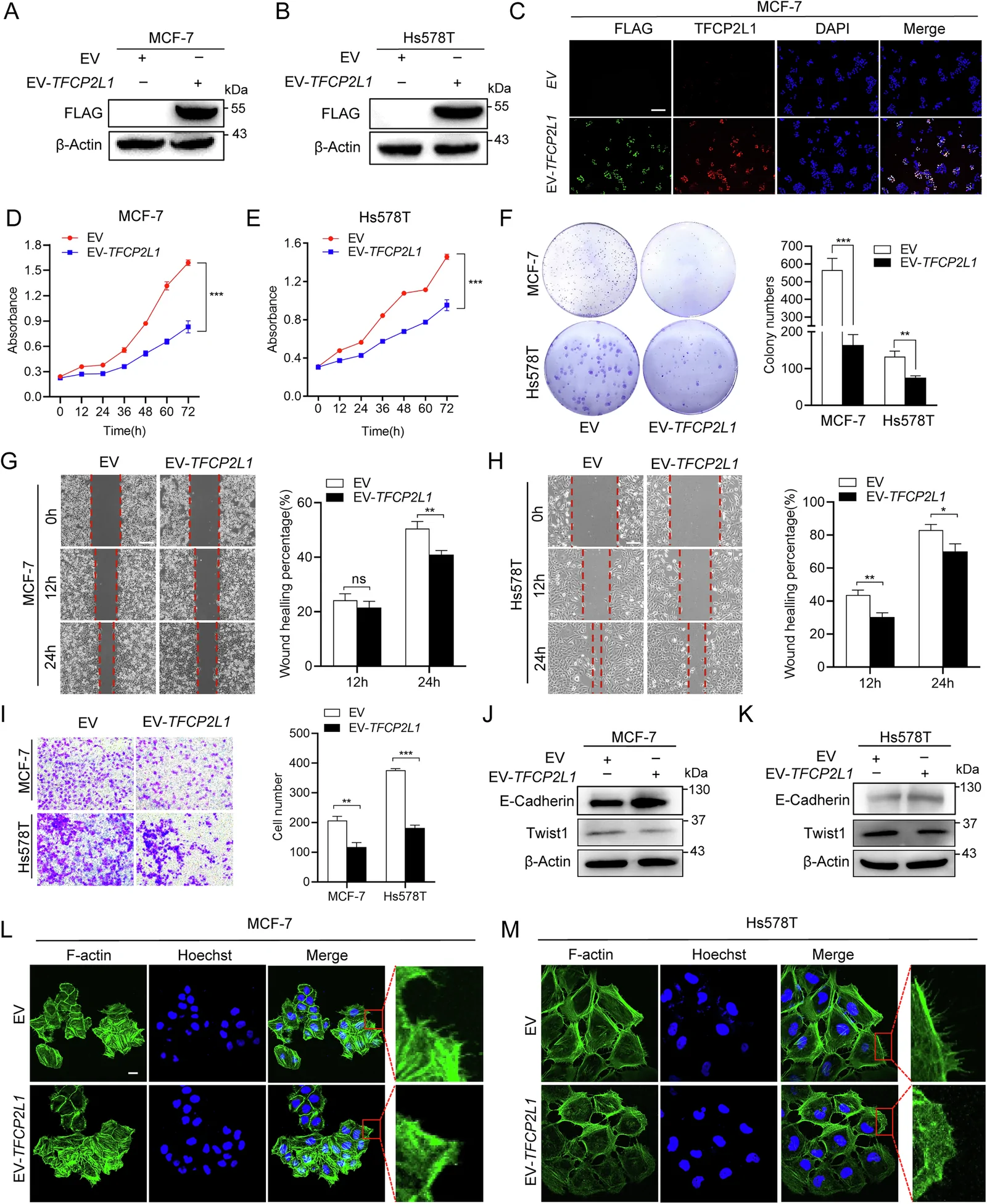
Overexpression of *TFCP2L1* inhibits the growth and migration of breast cancer cells. **A, B** Western blot analysis of FLAG in MCF7 and Hs578T cells transfected with FLAG-tagged human *TFCP2L1* (EV-*TFCP2L1*) or empty vector (EV). β-Actin served as a loading control. **C** Immunofluorescence staining for FLAG and TFCP2L1 in EV-*TFCP2L1*-transfected MCF-7 cells. Nuclei were stained using Hoechst 33342 (Hoechst). Bar, 50 μm. **D‒F** CCK8 **D, E** and colony-formation **F** assays of the proliferation of EV- and EV-*TFCP2L1-*transfected MCF-7 and Hs578T cells (*n* = 3). **G‒I** Wound-healing **G,H** and transwell **I** assays analyses of the migration of EV- and EV-*TFCP2L1-*transfected MCF-7 and Hs578T cells (*n* = 3). Bar, 50 μm. **J, K** Western blot analysis of E-Cadherin and Twist1 protein levels in EV- and EV-*TFCP2L1-*transfected MCF-7 and Hs578T cells. **L, M** Phalloidin staining of EV- and EV-*TFCP2L1*-transfected MCF-7 and Hs578T cells. Bar, 50 μm. The data are presented as mean ± SD. Statistical analyses were performed using two-way ANOVA **D, E** and Student’s *t*-tests **F‒I**. *p < 0.05, **p < 0.01, ***p < 0.001, ns, not significant.

To preliminarily investigate the function of TFCP2L1 in breast cancer progression, FLAG-tagged human *TFCP2L1* inserted into an empty vector (EV-*TFCP2L1*) was overexpressed in two breast cancer lines, MCF-7 and Hs578T, and empty vector (EV)-transfected cells were used as controls (Fig. 2A,B; Fig. S1B,C). Immunofluorescence staining revealed predominant nuclear localization of *TFCP2L1* overexpression in most MCF-7 and Hs578T cells (Fig. 2C; Fig. S1D). Subsequent Cell Counting Kit-8 (CCK8) and colony formation assays demonstrated growth inhibition in both cell lines with *TFCP2L1* overexpression compared with that in EV-transfected group (Fig. 2D−F). Furthermore, wound healing and transwell assays revealed a significant reduction in migration of MCF-7 and Hs578T cells transfected with EV-*TFCP2L1* (Fig. 2G−I). Western blot analysis of epithelial‒mesenchymal transition (EMT)-associated genes revealed a significant increase in E-Cadherin protein levels, whereas the protein levels of twist family bHLH transcription factor 1 (Twist1) were decreased in EV-*TFCP2L1-*transfected MCF-7 and Hs578T cells (Fig. 2J,K; Fig. S1E,F). In addition, Phalloidin staining experiments were performed on EV- and EV-*TFCP2L1-*trasfected cells and revealed a reduction in filamentous pseudopodia formation on the surface of *FLAG‒TFCP2L1*-overexpressing cells compared with control cells, indicating that the mobility of cells induced by *TFCP2L1* overexpression decreased (Fig. 2L,M). Interestingly, overexpression of *TFCP2L1* also inhibits the expression of *CD133*, a cancer stem cell stemness marker gene (Fig. S1G,H). Taken together, these findings suggest that forced expression of *TFCP2L1* suppresses the metastasis of breast cancer cells.

### Knockdown of TFCP2L1 promotes the aggressiveness of breast cancer cells in vitro

**Fig. 3:**
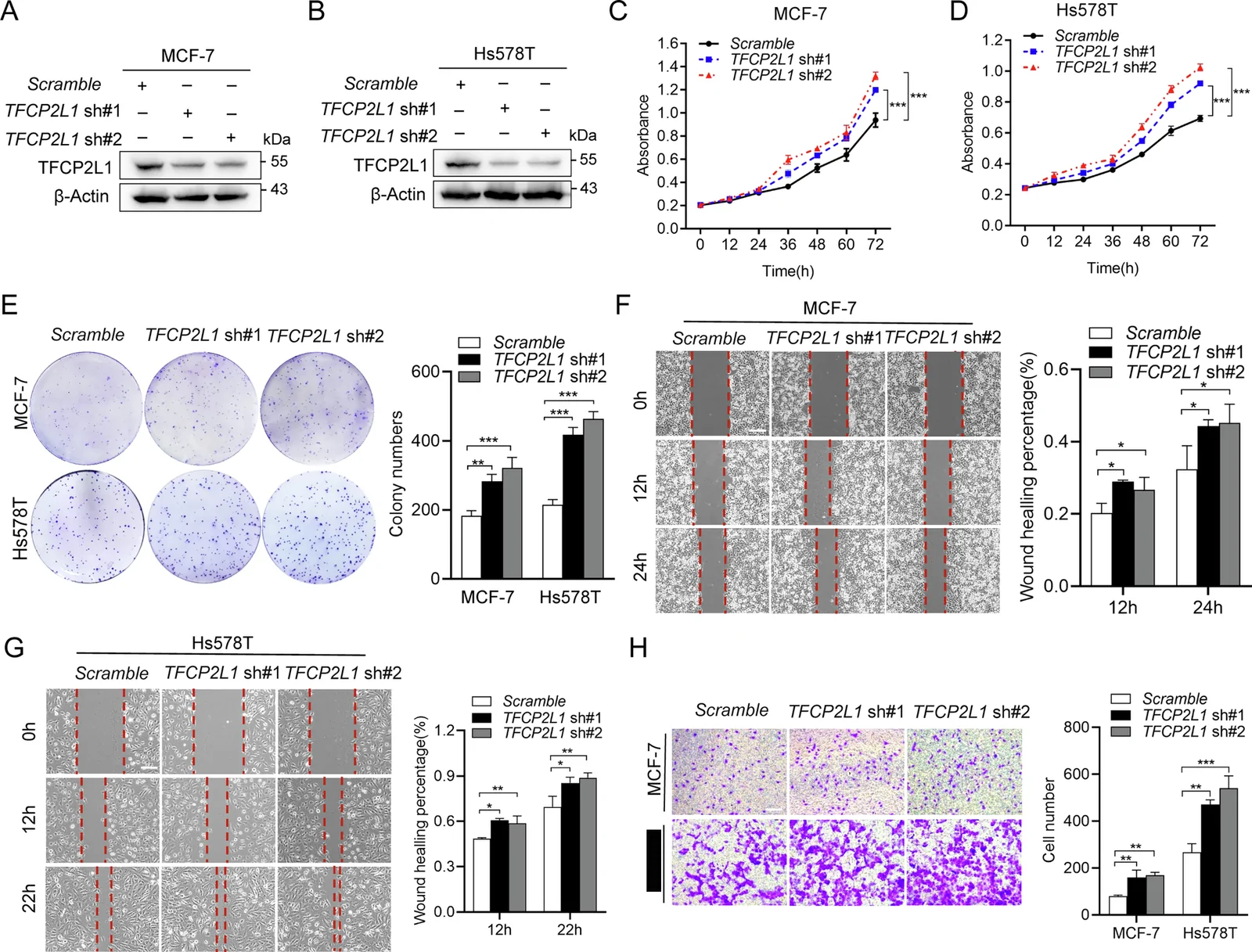
Low levels of *TFCP2L1* facilitate the proliferation and migration of breast cancer cells. **A, B** Western blot analysis of TFCP2L1 levels in *scramble* and *TFCP2L1* shRNA lentivirus-infected MCF7 and Hs578T cells. **C**–**E** CCK8 **C, D** and colony-formation **E** assays of the proliferation of *scramble* and *TFCP2L1* shRNA-transfected MCF7 and Hs578T cells (*n* = 3). **F**–**H** Wound healing **F, G** and transwell **H** assays of the migration of *scramble* and *TFCP2L1* shRNA-expressing MCF7 and Hs578T cells (*n* = 3). Bar, 50 μm. The data are presented as mean ± SD. Statistical analyses were performed using two-way ANOVA **C, D** and one-way ANOVA **E–H**. *p < 0.05, **p < 0.01, ***p < 0.001.

To investigate whether downregulation of *TFCP2L1* facilitates breast cancer cell proliferation and migration, we designed two short hairpin RNA (shRNA) interference sequences targeting the human *TFCP2L1* gene with lentiviral systems (*TFCP2L1* sh#1 and *TFCP2L1* sh#2). Compared with those of the scramble control, the expression levels of *TFCP2L1* were reduced by approximately 60–80% after infection with the *TFCP2L1* shRNA lentivirus (Fig. 3A,B; Fig. S2A–D). CCK8 and colony formation assays revealed that the growth of cells infected with *TFCP2L1* shRNA lentiviruses was significantly greater than that of scramble cells in the control group (Fig. 3C−E). Additionally, wound healing and transwell assays revealed that *TFCP2L*1 knockdown significantly accelerated the migration of MCF-7 and Hs578T cells (Fig. 3F−H). Similar results were observed in MCF-10A cells when *TFCP2L1* expression was knocked down (Fig. S2E), the migration capacity of the normal breast cells was reduced, and proliferation slowed (Fig. S2F,G). Overall, these results suggest that reduced *TFCP2L1* expression promotes the growth and metastasis of breast cancer cells.

### Ectopic expression of *TFCP2L1* decreases the tumor size and suppresses lung metastasis of breast cancer cells in vivo

**Fig. 4:**
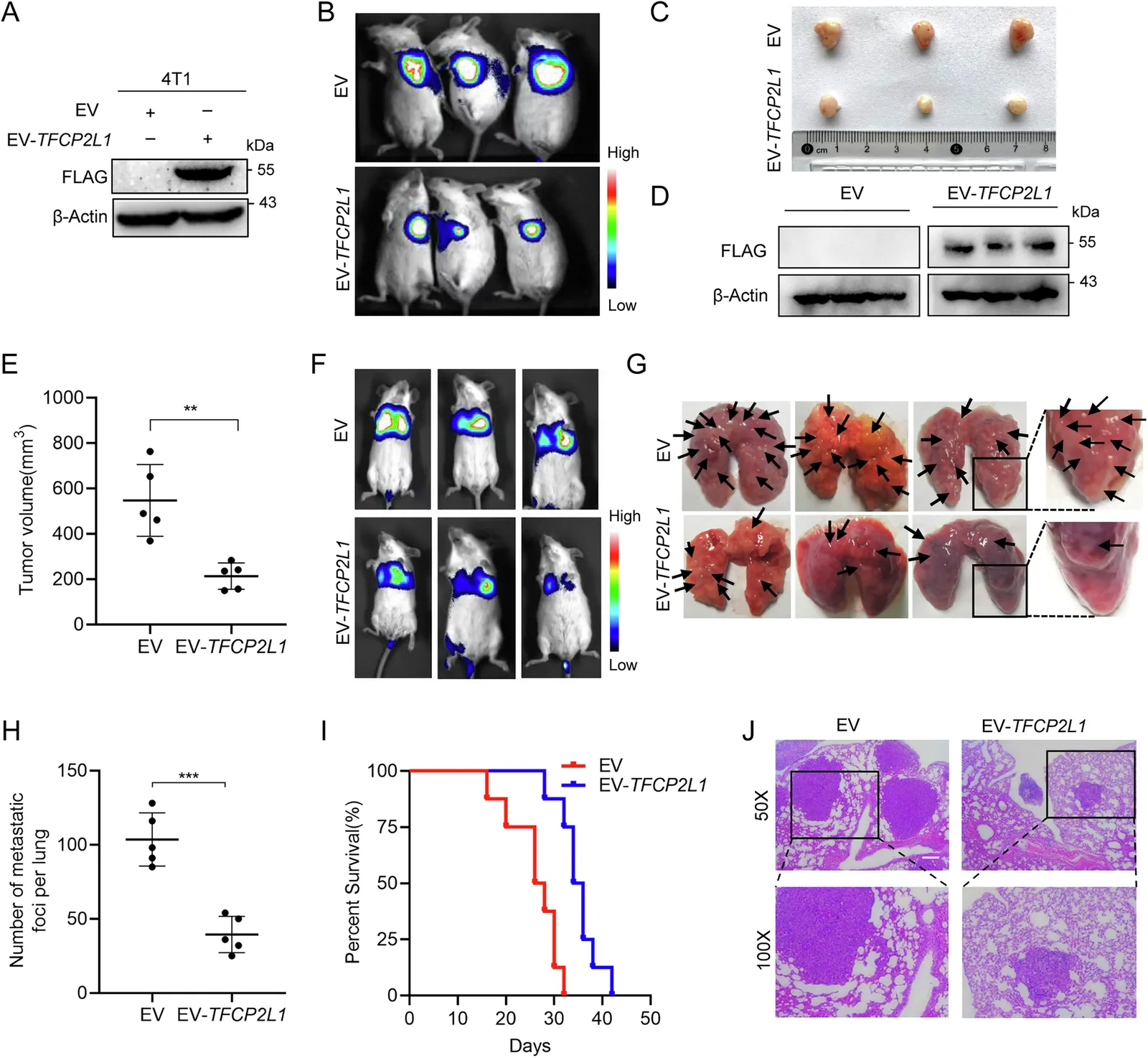
Overexpression of *TFCP2L1* decreases the tumor size and suppresses lung metastasis of breast cancer cells in mice. **A** Western blot analysis of FLAG in luciferase-labeled 4T1 cells overexpressing EV or EV-*TFCP2L1*. **B** Subcutaneous injection of the EV- and EV-*TFCP2L1*-transfected luciferase-labeled 4T1 breast cancer cells into BALB/c mice. Luciferase signals were captured with a Tanon-5200Multi system. **C** Dissected tumors from the mice in Fig. 4B. **D** Western blot analysis of FLAG in the different tumor tissues shown in Fig. 4C. **E** Measurement of tumor volume tumors carrying EV or EV-*TFCP2L1*-expressing cassette (*n* = 5). **F** Injection of EV- and EV-*TFCP2L1*-transfected 4T1 breast cancer cells into BALB/c mice via tail vein. Luciferase signals were captured via a Tanon-5200Multi system. **G** Representative gross view of lung samples with metastatic nodules (black arrows, metastatic nodules). **H** Quantification of pulmonary metastatic lesions (*n* = 5). **I** Overall survival of mice injected with cells via the tail vein. **J** Hematoxylin and eosin-stained lung tissue slides. Scale bar of upper panel, 400 μm. The data are presented as mean ± SD. Statistical analyses were performed using Student’s *t*-tests were used for analysis **E**,**H**, **p < 0.01, ***p < 0.001.

To further elucidate the comprehensive function of *TFCP2L1* in vivo, human *TFCP2L1* was transfected into the mouse breast cancer cell line 4T1, as the amino acid homology between the mouse and human TFCP2L1 proteins reached 93.53% (Fig. 4A) [25]. Subsequently, we subcutaneously injected cells from different groups into the breast pads of mice. After 2 weeks, the bioluminescence signal of the tumors derived from EV-*TFCP2L1-*transfected 4T1 cells was significantly lower than that of the control group (Fig. 4B). The EV-*TFCP2L1-*expressing cells-derived tumors still had high levels of FLAG and TFCP2L1 fusion proteins, and the tumor volume was also significantly lower than that of the EV-expressing cells-derived tumors (Fig. 4C‒E). Then, we injected different groups of 4T1 cells into the tail vein of the mice to examine the lung metastasis ability of the breast cancer cells. After 2 weeks, we assessed the bioluminescence intensity and collected gross lung samples to calculate the number of metastatic nodules before the mice were euthanized. The bioluminescence signal of the tumors with *TFCP2L1* overexpression was significantly weaker than that of the control group, and the number of pulmonary metastatic nodules was obviously reduced (Fig. 4F‒H). Survival analysis demonstrated a significant prolongation in the survival time of mice injected with EV-*TFCP2L1*-transfected 4T1 cells (Fig. 4I). Additionally, lung sections stained with hematoxylin and eosin revealed smaller and fewer lung metastatic nodules in the *TFCP2L1-*overexpressing group than in the control group (Fig. 4J). Overall, these findings indicate that high levels of *TFCP2L1* suppress breast cancer cell metastasis in vivo.

### Upregulation of *TFCP2L1* stimulates ferroptosis to inhibit breast cancer cell metastasis

**Fig. 5:**
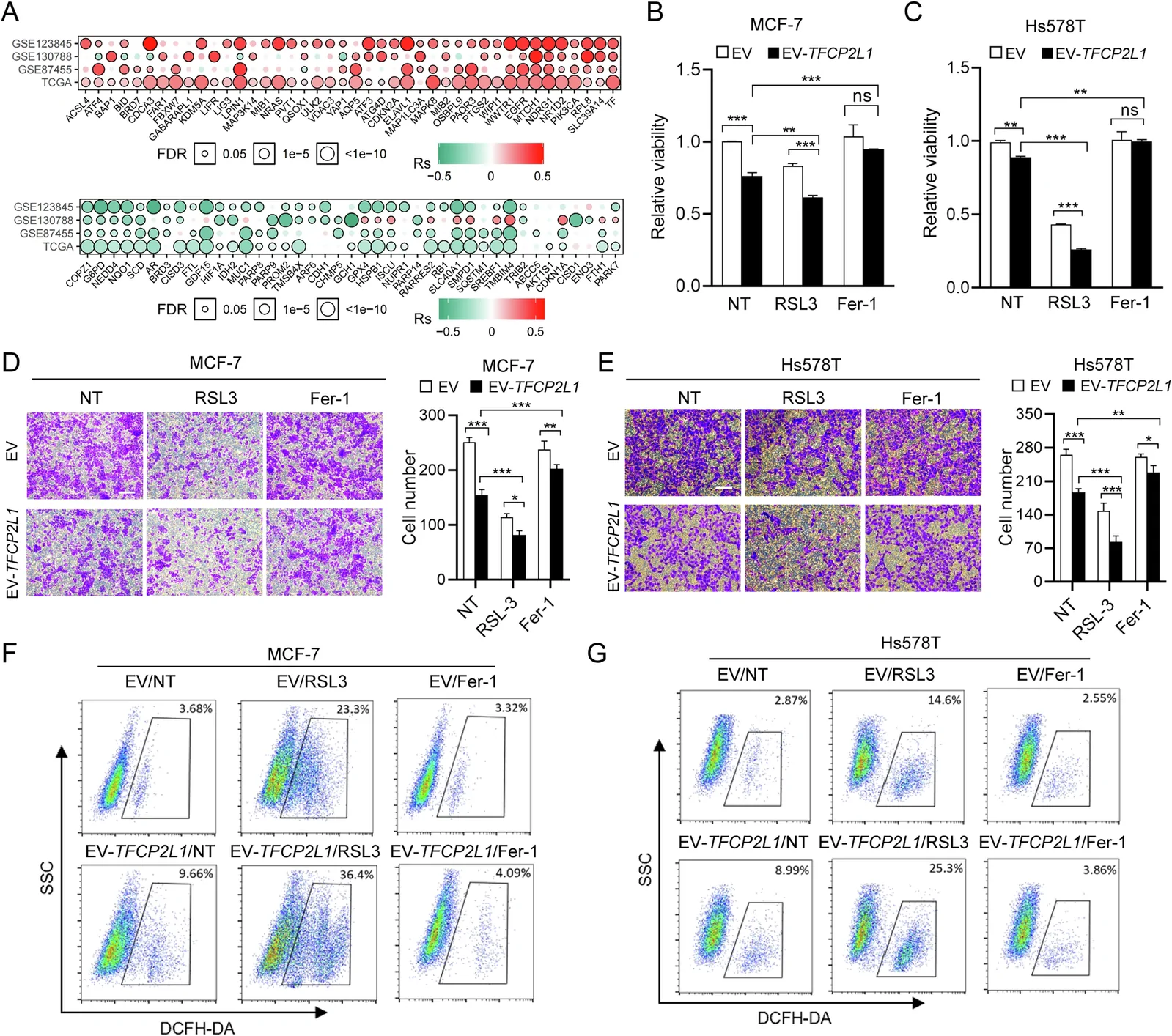
Ferroptosis mediates the effect of TFCP2L1 on breast cancer cells. **A** GSEA revealing the enrichment of ferroptosis-related genes in different subtypes of breast cancer patients. **B, C** Relative viability of EV- and EV-*TFCP2L1*-transfected MCF-7 and Hs578T cells treated with 5 μM Fer-1 or 5 μM RSL-3 for 12 h (*n* = 3). NT, No Treatment. **D, E** Transwell analysis of the migration of EV- and EV-*TFCP2L1*-transfected MCF-7 or Hs578T cells treated with or without 5 μM Fer-1 or 5 μM RSL-3 (*n* = 3). Bar, 50 µm. **F, G** Flow cytometry analysis of EV- and EV-*TFCP2L1*-transfected MCF-7 and Hs578T cells stained with a BODIPY fluorescent probe and treated with 5 μM Fer-1 or 5 μM RSL-3. The data are presented as mean ± SD. Statistical analyses were performed using two-way ANOVA **B‒E**. *p < 0.05, **p < 0.01, ***p < 0.001.

To explore the potential mechanism underlying the *TFCP2L1*-induced inhibition of cancer invasion, we analyzed data from the TCGA and public databases of breast cancer (GSE123845, GSE130788, and GSE84755) via gene set enrichment analysis (GSEA), and identified the enrichment of numerous ferroptosis-related genes in different breast cancer patient subtypes (Fig. 5A). Previous studies have linked oxidative phosphorylation and reduced ‌Reactive Oxygen Species (ROS) levels to the inhibition of ferroptosis [26]. Recent findings suggest that the inhibition of ferroptosis promotes metastasis in breast cancer [8–10]. Therefore, we hypothesized that ferroptosis might be a crucial mechanism underlying the *TFCP2L1*-induced inhibition of breast cancer metastasis. To validate this hypothesis, we initially treated MCF-7 and Hs578T cells with a ferroptosis-specific inducer RSL-3. Treatment of breast cancer cells with varying concentrations of RSL3 revealed massive cell death at 6 μM in Hs578T cells, while concentrations of 3–5 μM induced growth retardation and sporadic cell death. In addition, Hs578T cells exhibited greater sensitivity to the treatment compared to MCF-7 cells. Therefore, 5 μM RSL3 was selected for subsequent experiments (Fig. S3A). Compared with that in the control group, the cell viability of the cells in the *TFCP2L1-*overexpressing group increased after Ferrostatin-1 (Fer-1) treatment, a specific inhibitor of ferroptosis, but significantly decreased after RSL-3 treatment (Fig. 5B,C). Neither the apoptosis inhibitor Z-VAD-FMK nor the necroptosis inhibitor Necrostatin-1 can reverse the inhibition of breast cancer cell proliferation by TFCP2L1 (Fig. S3B). Wound healing and transwell assays revealed that the effects of *TFCP2L1* overexpression on cell migration could also be inhibited by Fer-1 (Fig. 5D,E; Fig. S3C,D). Flow cytometry revealed a significant increase in ROS production in the *TFCP2L1*-overexpressing cells compared with the control cells, and RSL-3 further increased the ROS levels (Fig. 5F,G; Fig. S3E,F). However, Fer-1 addition reduced ROS production in the *TFCP2L1*-overexpressing group (Fig. 5F,G; Fig. S3E,F). In line with these results, fluorescence microscopy analysis revealed a significant increase in the fluorescence intensity of ROS in EV-*TFCP2L1*-overexpressing and RSL3-treated MCF-7 and Hs578T cells, whereas Fer-1 treatment significantly reduced the fluorescence intensity of ROS (Fig. S3G,H). Moreover, although overexpression of *TFCP2L1* did not induce significant loss of mitochondria in MCF-7 cells, it enhanced the response of mitochondria to RSL3, resulting in more severe shrinkage (Fig. S4A). These findings collectively indicate that the upregulation of *TFCP2L1* promotes ferroptosis in breast cancer cells.

### *TFCP2L1* directly inhibits *GPX4* transcription in breast cancer cells

**Fig. 6:**
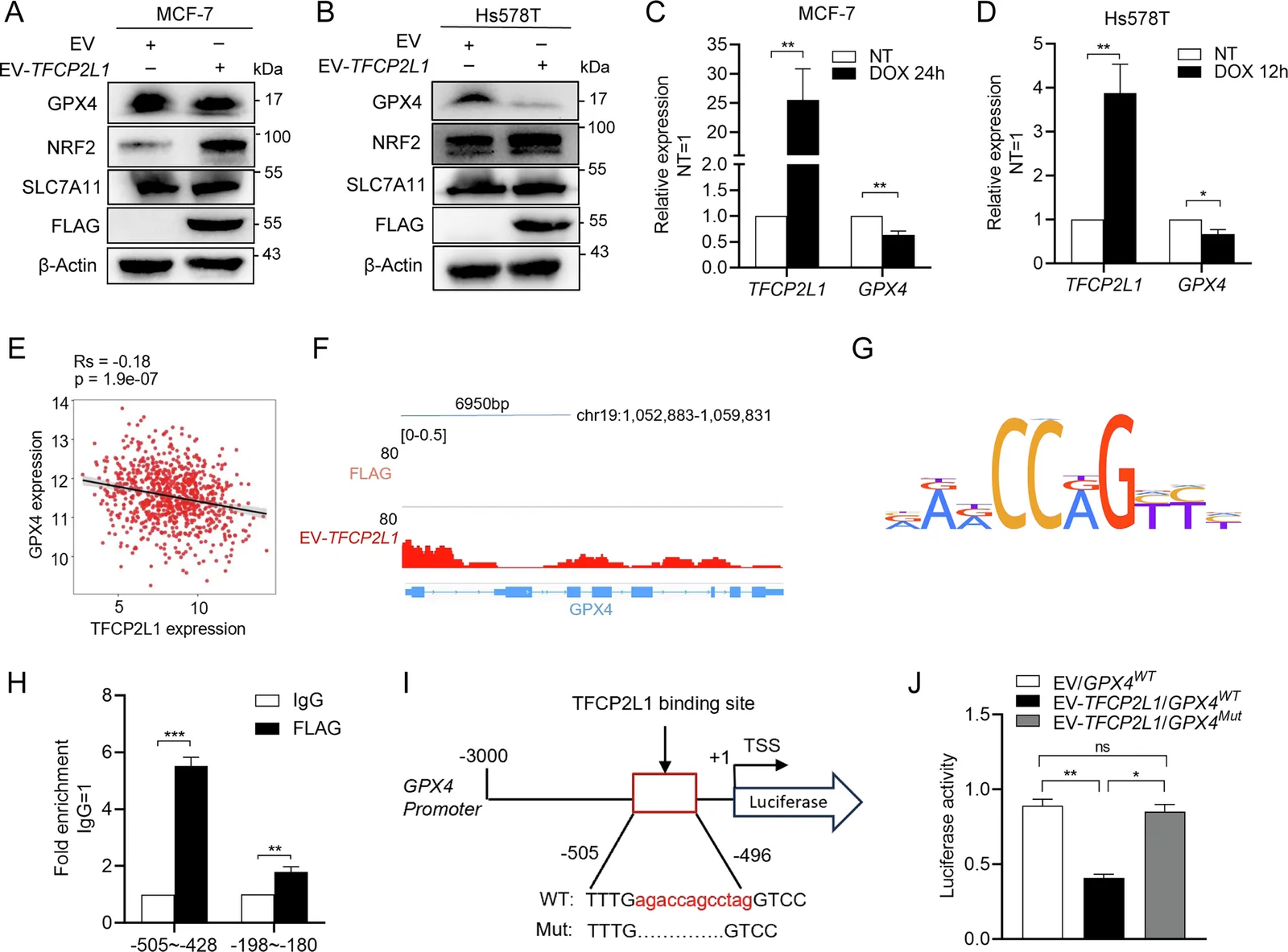
GPX4 is a direct target of TFCP2L1. **A, B** Western blot analysis of the protein levels of GPX4, NRF2, SLC7A11 and TFCP2L1 in EV- and EV-*TFCP2L1*-transfected MCF-7 and Hs578T cells. **C, D** RT‒qPCR analysis of *TFCP2L1* and *GPX4* expression in *i-TFCP2L1*-transfected MCF-7 and Hs578T cells treated with or without 2 μg/ml doxycycline (DOX) (*n* = 3). **E** Correlation analysis of the mRNA levels of *TFCP2L1* and *GPX4* in clinical breast cancer samples. **F** CUT&Tag‒sequencing analysis of the enrichment of TFCP2L1 in the GPX4 promoter indicator region. **G** Predicted consensus binding motif of TFCP2L1 target loci from the HOCOMOCO website. **H** ChIP–RT‒qPCR analysis of the degree of enrichment of TFCP2L1 in the indicated regions of the GPX4 promoter (*n* = 3). **I** Positions of one putative TFCP2L1-binding site in the GPX4 promoter and the corresponding mutant sequence. **J** Luciferase activity analysis of EV or EV-*TFCP2L1*-transfected cells overexpressing the wild-type (WT) or mutant (Mut) GPX4 promoter reporter plasmid (*n* = 3). The data are presented as mean ± SD. Statistical analyses were performed using Student’s *t*-tests **C,D,H** and one-way ANOVA **J**. *p < 0.05, **p < 0.01, ***p < 0.001.

To explore in greater detail the mechanism by which *TFCP2L1* upregulation promotes ferroptosis, we initially examined the expression of several key factors implicated in the pathogenesis of ferroptosis in MCF-7 and Hs578T cells. Surprisingly, we observed decreased GPX4 protein levels in MCF-7 and Hs578T cells stably overexpressing *TFCP2L1* (Fig. 6A,B; Fig. S4B,C). Additionally, RT‒qPCR analysis revealed similar results (Fig. S4D,E). Conversely, knockdown of *TFCP2L1* increased *GPX4* levels in MCF-7 cells (Fig. S4F,G). Next, we established a *TFCP2L1* gene-inducible cell line (*i-TFCP2L1*) using the PiggyBac (PB) ‒TETON system, in which exogenous *TFCP2L1* can be effectively induced upon treatment with doxycycline (Dox) (Fig. 6C,D). Concurrently, we observed downregulation of *GPX4* levels in Dox-treated cells (Fig. 6C,D). Furthermore, we validated the negative correlation between *TFCP2L1* and *GPX4* in the clinical breast cancer tissues (Fig. 6E).

To investigate whether the transcription of *GPX4* is directly regulated by *TFCP2L1*, CUT&Tag sequencing was performed and the results revealed enrichment of TFCP2L1 at the promoter region of GPX4 (Fig. 6F). Additionally, we searched the HOCOMOCO website (https://hocomoco11.autosome.ru/), which provides a complete collection of transcription factor binding models for humans and mice identified via large-scale chromatin immunoprecipitation (ChIP)-sequencing analysis, to analyze the consensus binding motifs of *TFCP2L1* (Fig. 6G) and predict two potential binding sites within GPX4 promoter regions (from -3000 to +1). Motif 1 is located at -505 to -496, and motif 2 is located at -198 to -180. We subsequently conducted ChIP in EV-*TFCP2L1-*transfected MCF-7 cells using the FLAG antibody and observed significant enrichment of motif 1 (Fig. 6H). To confirm the direct effect of *TFCP2L1* on *GPX4* transcription, we transfected MCF-7 cells with a luciferase reporter vector driven by the *GPX4* promoter fragment (pGL6‒GPX4), containing either wild-type motif 1 (WT) or a mutant motif 1 sequence (Mut), in the presence or absence of EV-*TFCP2L1* (Fig. 6I). Under these conditions, we observed that *TFCP2L1* upregulation reduced the luciferase activity driven by GPX4^WT^ compared with that in cells transfected with only pGL6‒GPX4^WT^ (Fig. 6J). Conversely, EV-*TFCP2L1*-transfected cells harboring pGL6‒GPX4^Mut^ plasmid presented stronger luciferase activity than those harboring the pGL6‒GPX4^WT^ plasmid did (Fig. 6J). Collectively, these data indicate that *GPX4* is a target of *TFCP2L1* and the latter directly suppresses *GPX4* expression in breast cancer cells.

### Increased expression of *GPX4* restored the inhibitory effects of *TFCP2L1* on breast cancer cell progression

**Fig. 7:**
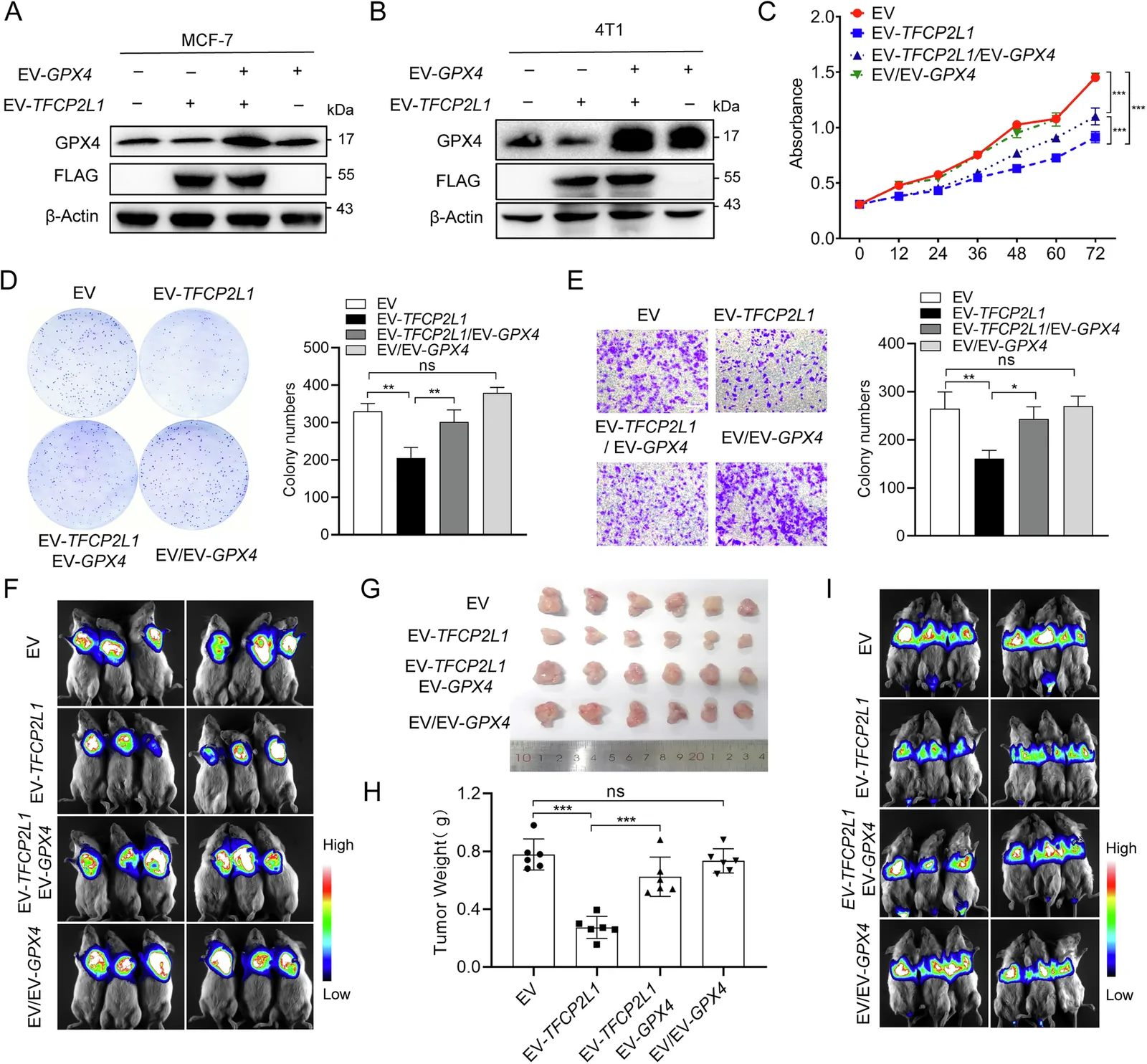
GPX4 mediates the effects of TFCP2L1 on breast cancer cells. **A,B** Western blot analysis of the protein levels of GPX4 and FLAG in EV-, EV-*TFCP2L1-* or/and EV-*GPX4*-transfected MCF-7 cells **A** and luciferase-labeled 4T1 cells **B**. **C** CCK8 assay analysis of the proliferation of MCF-7 cells transfected with EV, EV-*TFCP2L1* or/and EV-*GPX4*. **D** Colony formation assay analysis of the proliferation of MCF-7 cells transfected with EV, EV-*TFCP2L1* or/and EV-*GPX4* (*n* = 3). **E** Transwell assay analysis of the migration of MCF-7 cells transfected with EV, EV-*TFCP2L1* or/and EV-*GPX4* (*n* = 3). Bar, 50 µm. **F** Representative bioluminescence images of mice injected subcutaneously with luciferase-labeled 4T1 cells transfected with EV, EV-*TFCP2L1* or/and EV-*GPX4*. **G** Dissected tumors from the mice in Fig. 7F. **H** Comparison of the average weights of the tumors in the different groups shown in Fig. 7G (*n* = 6). **I** Representative bioluminescence images of mice injected with luciferase-labeled 4T1 cells transfected with EV, EV-*TFCP2L1* or/and EV-*GPX4* via the tail vein. The data are presented as mean ± SD. Statistical analyses were performed using two-way ANOVA **C** and one-way ANOVA **D**,**E**,**H**. *p < 0.05, **p < 0.01, ***p < 0.001.

To validate this perspective, we overexpressed *GPX4* in EV-*TFCP2L1*-transfected MCF-7 and 4T1 cells (Fig. 7A,B; Fig. S4H,I). First, we assessed the combined biological effects of *TFCP2L1* and *GPX4* using the CCK8 and colony formation assays. The results showed that following transfection with *GPX4*, the proliferation capacity of MCF-7 cells, hindered by *TFCP2L1* overexpression, was restored (Fig. 7C,D). In addition, GPX4 upregulation counteracted the inhibitory effects of *TFCP2L1* overexpression on MCF-7 cell migration (Fig. 7E). In line with the in vitro observations, the subcutaneous tumor size and degree of lung metastasis were notably smaller and low, respectively, in the *TFCP2L1*-overexpressing 4T1 cell group than in the *EV*-transfected 4T1 group, as revealed by quantitative analysis by weighing and imageJ software (Fig. 7F‒I; Fig. S5A‒C), while the effects of TFCP2L1 could be mitigated by *GPX4* overexpression (Fig. 7F‒I; Fig. S5A‒C). Importantly, mouse Tfcp2l1 has a function similar to that of human TFCP2L1 in 4T1 breast cancer cells when overexpressed (Fig. S6A). Specifically, overexpression of FLAG-tagged mouse *Tfcp2l1* (EV-*mTfcp2l1*) significantly inhibited the ability of the 4T1 cell line to form tumors in mice (Fig. S6B‒E). However, overexpression of mouse *Gpx4* (EV-*mGpx4*) alleviated the effect of EV-*mTfcp2l1* (Fig. S6B‒E). Similarly, human *TFCP2L1*-overexpressing Hs578T cells exhibited lower subcutaneous tumor-forming capacity in nude mice compared to EV control cells, but *GPX4* overexpression restored the tumorigenic ability of *TFCP2L1*-overexpressing Hs578T cells (Fig. S6F‒H). Furthermore, when EV-*TFCP2L1*-expressing Hs578T cells were injected into nude mice via the tail vein, their ability to metastasize to the lungs was reduced; however, *GPX4* overexpression enhanced the lung metastasis capacity of these cells (Fig. S6I,J). Taken together, these findings indicate that the upregulation of *TFCP2L1* hampers the progression of breast cancer by suppressing the expression of *GPX4* both in vitro and in vivo.

### *TFCP2L1* indirectly reduces the synthesis of *GPX4* by partially inhibiting the PI3K/AKT signaling pathway

**Fig. 8:**
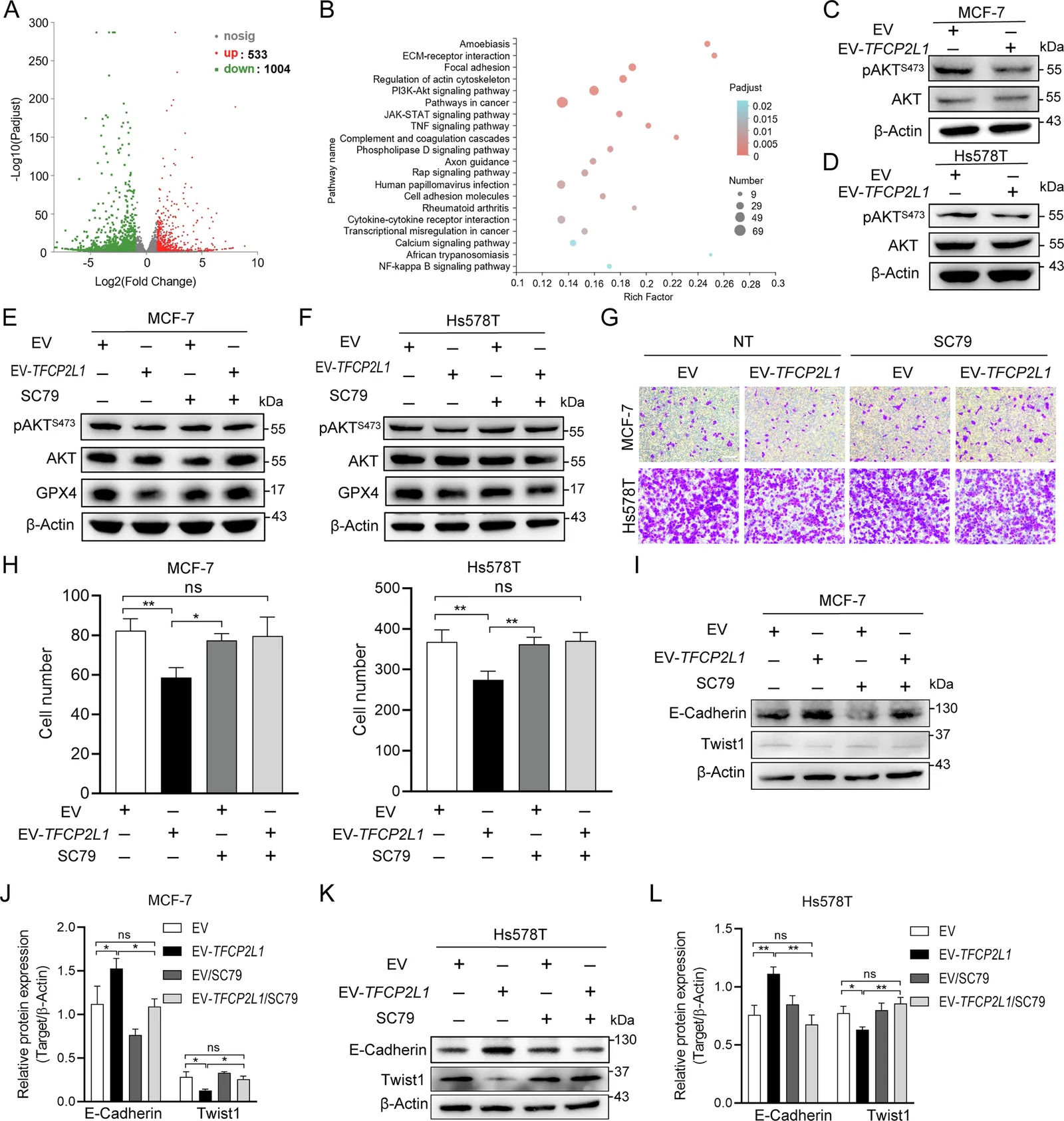
Activation of the PI3K/AKT signaling pathway mediates the function of TFCP2L1 in breast cancer cells. **A** Volcano map showing the DEGs in Hs578T cells regulated by *TFCP2L1*. **B** KEGG pathway analysis of the DEGs regulated by *TFCP2L1*. **C, D** Western blot analysis of the total protein levels of AKT and phosphorylated AKT in MCF-7 and Hs578T cells overexpressing EV or EV-*TFCP2L1*. **E, F** Western blot analysis of the protein levels of AKT, pAKT^S473^ and GPX4 in EV- or EV-*TFCP2L1*-transfected MCF-7 and Hs578T cells in the presence or absence of 10 μM SC79. **G** Transwell assay analysis of the migration of EV- or EV-*TFCP2L1-*transfected MCF-7 and Hs578T cells treated with or without SC79 (*n* = 3). Bar, 50 µm. **H** Quantification of EV- or EV-*TFCP2L1*-transfected MCF-7 and Hs578T cells in Fig. 8G (*n* = 3). **I** Western blot analysis of the protein levels of E-Cadherin and Twist1 in EV- or EV-*TFCP2L1-*transfected MCF-7 cells treated with or without SC79. **J** Densitometric analysis of the relative protein levels of E-Cadherin and Twist1, as shown in Fig. 8I, was performed with ImageJ software. Protein levels were normalized to those of β-Actin (*n* = 3). **K** Western blot analysis of the protein levels of E-Cadherin and Twist1 in EV- or EV-*TFCP2L1-*transfected Hs578T cells treated with or without SC79. **L** Densitometric analysis of the relative protein levels of E-Cadherin and Twist1, as shown in Fig. 8K, was performed with ImageJ software. Protein levels were normalized to those of β-Actin (*n* = 3). The data are presented as mean ± SD. Statistical analyses were performed using one-way ANOVA **H,J,L**. *p < 0.05, **p < 0.01, ***p < 0.001.

To investigate whether TFCP2L1 inhibits breast cancer cell proliferation and migration via other mechanisms, the whole transcriptase profiles of EV- and EV-*TFCP2L1*-transfected cells were analyzed via RNA sequencing. The overexpression of *TFCP2L1* regulated a total of 1537 differentially expressed genes (DEGs) more than two-fold in Hs578T cells, including 533 upregulated genes and 1004 downregulated genes, compared with those in the EV control group (Fig. 8A). Kyoto Encyclopedia of Genes and Genomes (KEGG) analysis of the DEGs revealed that the PI3K/AKT signaling pathway was enriched (Fig. 8B). Western blot analysis and densitometric analysis showed that the relative expression level of phospho-AKT^T308^ (pAKT^T308^) and phospho-AKT^S473^ (pAKT^S473^) decreased in EV-*TFCP2L1*-transfected MCF-7 and Hs578T cells, whereas no significant change in the total protein level of AKT was observed compared with that in the EV control group (Fig. 8C,D; Fig. S7A–F). The expression of target genes of Janus kinase (JAK)/STAT3 and mitogen-activated protein kinase kinase/mitogen-activated protein kinase (MEK/ERK) signaling pathways was inconsistent between MCF-7 and Hs578T cells (Fig. S7G,H); therefore, we subsequently focused on AKT for further investigation. To examine whether activation of PI3K/AKT signaling can mediate the function of TFCP2L1, a rescue experiment utilizing the AKT agonist SC79 was conducted, and the results of Western blot analysis and densitometric analysis revealed that the addition of SC79 reversed the decrease in pAKT^T308^, pAKT^S473^ and GPX4 protein levels induced by the upregulation of *TFCP2L1* in MCF-7 and Hs578T cells (Fig. 8E,F; Fig. S7A–D,I,J). Furthermore, the effects of the PI3K/AKT pathway on cell migration were examined. Transwell assays revealed that SC79 restored the impaired migration ability of MCF-7 and Hs578T cells induced by *TFCP2L1* overexpression (Fig. 8G,H). Western blot analysis demonstrated that the overexpression of *TFCP2L1* induced the expression of the EMT-related proteins E-Cadherin and Twist1 in MCF-7 and Hs578T cells, which was reversed upon activation of the PI3K/AKT pathway (Fig. 8I‒L). Finally, we investigated the potential mechanism by which *TFCP2L1* inhibits PI3K/AKT signaling activity. RT-qPCR was used to detect genes related to this pathway enriched by KEGG, and the results showed that the expression of platelet derived growth factor subunit B (*PDGFB*) was significantly reduced (Fig. S7K,L). These findings collectively suggest that the influence of TFCP2L1 on GPX4 synthesis could also be mediated through the PI3K/AKT signaling pathway.

## Discussion

**Fig. 9:**
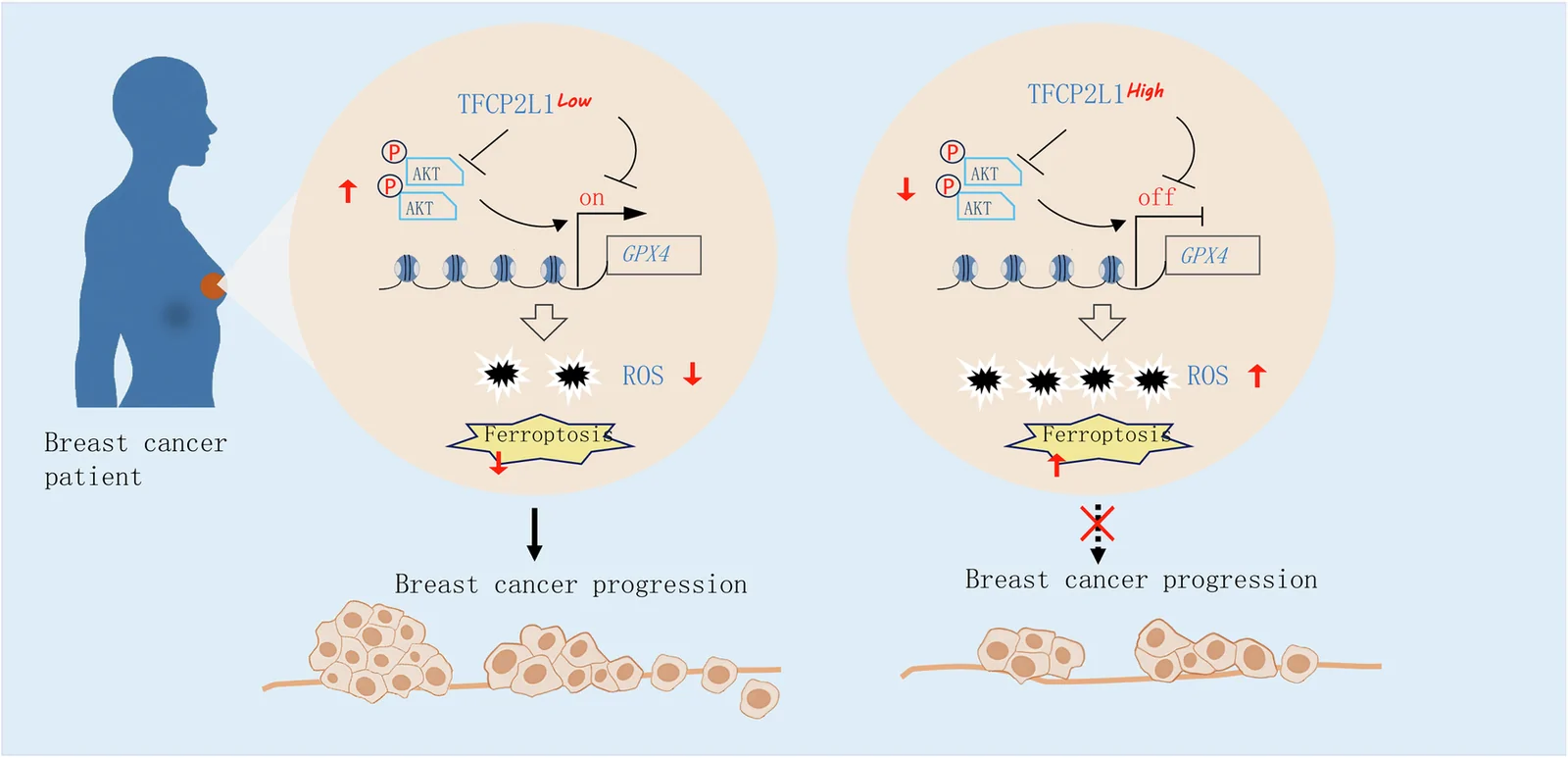
A schematic diagram of the working mode of TFCP2L1 in breast cancer progression. TFCP2L1 acts as a tumor suppressor via ferroptosis by directly inhibiting *GPX4* transcription or associating with the P3K/AKT signaling pathway to indirectly limit *GPX4* expression.

The self-renewal markers of embryonic stem cells often promote cancer cell proliferation and metastasis. To date, several studies have discussed the role of TFCP2L1 in tumorigenesis and cancer development. Notably, evidence has shown that TFCP2L1 plays a bifunctional role in different malignancies [19]. In some cancers, TFCP2L1 functions as a tumor suppressor. Tun et al. demonstrated that the downregulation of both the mRNA and protein levels of TFCP2L1 in clear renal cell carcinoma and the loss of TFCP2L1 might play crucial roles in epithelial dedifferentiation [20]. In thyroid cancer, Li et al. reported that circHACE1 acts as a sponge to absorb miR-346 and elevates *TFCP2L1* expression in differentiated thyroid cancer cells, thus inhibiting cell malignancy [27]. Additionally, TFCP2L1 silencing can counteract the suppressive effect of a miR-346 inhibitor on malignancy [27]. A recent study reported increased TFCP2L1 expression when C-X-C motif chemokine receptor 2 (CXCR2) activity was blocked in a melanoma mouse model, resulting in the downregulation of growth-related genes and the upregulation of tumor suppressor genes [23]. Conversely, TFCP2L1 acts as an oncogene in certain cancers. In bladder cancer, TFCP2L1 is phosphorylated by cyclin dependent kinase 1 (CDK1) and subsequently transcriptionally represses inhibitor of DNA binding 2 (*ID2*), thereby promoting cell proliferation, stemness, and invasion [22, 28]. In addition, TFCP2L1 can induce Nanog homeobox (*NANOG*) expression to activate the JAK/STAT3 pathway to enhance the proliferation of cell stem cells in hepatocellular carcinoma [24]. In this study, our results showed that upregulation of TFCP2L1 can inhibit the progression of breast cancer cells (Fig. 9). The different roles of TFCP2L1 in tumors from different tissues may be related to the distinct tissue microenvironments, in which the signals regulating *TFCP2L1* gene expression and the downstream regulatory networks mediated by TFCP2L1 are different.

Bioinformatic analysis revealed enrichment of ferroptosis-related genes in patients with different subtypes of breast cancer, which indicates that the inhibitory effect of TFCP2L1 on breast cancer may be related to ferroptosis (Fig. 5A). Ferroptosis, a recently identified iron-dependent programmed cell death pathway regulated by GPX4, is characterized by the accumulation of ROS and lipid peroxides within cells [29]. Morphologically, ferroptosis differs significantly from apoptosis, necrosis, autophagy, and other forms of cell death [30–32]. Increasing evidence suggests that the induction of ferroptosis can inhibit cancer cell proliferation, migration and invasion [33, 34]. According to these reports, Kruppel-like transcription factor 2 (KLF2) inhibits cancer cell migration and invasion by regulating ferroptosis through GPX4 in clear cell renal cell carcinoma [35]. Similarly, SIRT3 inhibits gallbladder cancer by inducing AKT-dependent ferroptosis and blocking the EMT [36]. Tripartite motif containing 34 (TRIM34) targeting enhances ferroptosis sensitivity and augments immunotherapy efficacy in hepatocellular carcinoma [37]. GPX4, a major ferroptosis regulator, uses glutathione to protect cells from ferroptosis by eliminating phospholipid peroxides [38]. Our results revealed that *TFCP2L1* could inhibit the transcription of *GPX4*, thereby promoting ferroptosis, contributing additional insights into GPX4 regulatory mechanisms.

The PI3K/AKT signaling cascade is crucial for various physiological processes, including cell cycle progression, differentiation, and metabolism [39]. PI3K/AKT pathway aberrations are frequently found in breast cancers and are associated with cellular transformation, tumorigenesis, cancer progression, and drug resistance [40]. The activation of the PI3K/AKT signaling pathway by transmembrane 4 L six family member 1 (TM4SF1) promotes the proliferation, migration and invasion of breast cancer cells [41]. 2D-CuPd nanoenzymes overcome tamoxifen resistance in cancer by regulating the PI3K/AKT/mTOR pathway [42]. Furthermore, PI3K/AKT signaling regulates ferroptosis, suggesting a promising avenue for cancer therapy [43]. KLF2 induces ferroptosis by inhibiting the PI3K/AKT pathway, thereby impeding cancer progression and metastasis [44]. AKT signaling is activated by upregulation of the chaperonin containing TCP1 subunit 3 (CCT3), which inhibits ferroptosis in lung adenocarcinoma cells, thereby increasing proliferation [45]. Notably, several recent studies have reported that GPX4 is a downstream gene of the PI3K/AKT signaling pathway in breast cancer [46–48]. The relationship between the PI3K/AKT signaling pathway and ferroptosis involves a dynamic regulatory system, which is highly important for treatment methods and disease management. In the present study, our results revealed that TFCP2L1 can regulate AKT, reducing its phosphorylation level to inhibit the transcription of GPX4 and regulate ferroptosis. Notably, the observation that AKT activator SC79 only partially reverses TFCP2L1‑mediated GPX4 downregulation and cell migration indicates that direct transcriptional repression by TFCP2L1 is the primary mechanism, whereas the indirect PI3K/AKT pathway serves to amplify the ferroptosis signal. The relative contribution of each axis may be context‑dependent, likely varying with the genetic background of different breast cancer subtypes.

In summary, the strong correlation of low *TFCP2L1* expression with poor prognosis supports TFCP2L1 as a promising prognostic biomarker. Moreover, its ability to suppress tumor growth and lung metastasis by inducing ferroptosis suggests that restoring TFCP2L1 or mimicking its activity could be a novel therapeutic strategy, particularly for triple‑negative breast cancer. However, several unanswered questions remain. First, the upstream molecular events linking TFCP2L1 to PI3K/AKT inhibition are not yet defined, although we observed that *TFCP2L1* overexpression significantly downregulates *PDGFB*, a known activator of PI3K/AKT signaling. Second, the current xenograft models do not fully recapitulate the tumor immune microenvironment, where ferroptosis may interact with antitumor immunity. Addressing these issues will be essential for future translational development.

## Materials and methods

### Cell lines and cell culture

The breast cancer cell lines Hs578T, MCF-7, MDA-MB231, and ZR-75-1 were obtained from the National Collection of Authenticated Cell Cultures (Chinese Academy of Sciences) and maintained in DMEM (2326200, Viva Cell, China) supplemented with 10% FBS (FSP500, ExCell Bio, Australia). The STR profile of MCF-7 and Hs578T were validated by sequencing. The breast cell line MCF-10A and the human embryonic kidney cell line HEK-293T were obtained from Thermo Fisher Scientific and maintained in the same medium as the breast cancer cells were. All the cells were maintained in a humidified incubator with 5% CO2 at 37°C. Mycoplasma contamination was checked monthly to ensure cell line purity.

### Plasmid construction

Mouse and human *TFCP2L1* and *GPX4* coding regions were inserted into the PiggyBac system (PB510B-1, System Bioscience) or PCDH lentivirus vectors (PVT11075, Nova Lifetech) tagged with FLAG. To induce RNA interference in breast cancer cells, shRNA constructs targeting specific regions of TFCP2L1 were designed and cloned and inserted into pLKO.1-TRC (#10878, Addgene). The primer sequences are listed in Tables S1 and S2.

### Cell transfection and lentiviral infection

The target gene plasmid (2 μg) was transfected into HEK-293T cells using Hieff Trans TM Liposomal Transfection Reagent (40802ES03, YEASEN, China) along with the auxiliary plasmids psPAX2 (1.25 μg) and pMD2.G (0.75 μg). After two days, the virus was harvested. When the breast cancer cell density reached 60%, lentivirus was added to the culture medium in the presence of 8 μg/ml polybrene. Stable infected cells were selected with 2 μg/ml puromycin or/and 10 μg/ml blasticidin S HCl after 48 h.

### Western blot

Cells were lysed in ice-cold RIPA cell buffer (P0013B, Beyotime Biotechnology, China) supplemented with a protease inhibitor cocktail. The protein samples were separated on a 10% or 15% SDS‒PAGE gel and electrotransferred to PVDF membranes, which were then blocked with 5% skim milk. Following blocking, the membrane was incubated overnight at 4 °C with specific primary antibodies. The secondary antibody was incubated at room temperature for 1 h. Immunoreactive bands were visualized via a Chemiluminescence Gel Imaging System (Tanon 5200multi, Shanghai). The primary antibodies are FLAG (F1804, Sigma, 1:2000), TFCP2L1 (AF5726, R&D Systems, 1:1000), TFCP2L1 (UPA02723, General Biol, China, 1:500), β-Actin (380624, ZENBIO, 1:1000), E-Cadherin (20874-1-AP, Proteintech, 1:1000), Twist1 (25465-1-AP, Proteintech, 1:1000), GPX4 (381958, ZENBIO, 1:1000), NRF-2 (380773, ZENBIO, 1:1000), SLC7A11 (HA60098, HUABIO, 1:1000), AKT (R23412, ZENBIO, 1:1000) and Phospho-Akt (Ser473) (R22961, ZENBIO, 1:1000).

### Reverse transcription–quantitative PCR

Total RNA was isolated via a total RNA extraction kit (19221ES50, Yeasen Biotechnology, China), and 1 µg of the isolated RNA was reverse transcribed into cDNA using the cDNA Synthesis SuperMix for qPCR kit (R333-01, Vazyme, China). Quantitative RT-PCR was performed using the Universal SYBR qPCR Master Mix reagent (11201ES03, Yeasen Biotechnology). Each experiment was conducted in triplicate. The relative expression was determined by the 2-DCq method and normalized to *β-Actin* expression. The primer sequences are listed in Table S3.

### Immunofluorescence staining

The cells were washed twice with PBS, fixed in 4% paraformaldehyde for 30 min, and then washed twice with PBS. The primary antibodies were diluted in blocking buffer containing 5% BSA and 0.2% Triton X-100, and incubated overnight at 4°C. After being washed twice with PBS, the cells were incubated with a specific fluorescent secondary antibody (1:1000) and Hoechst 33342 (H3570, Invitrogen, 1:10000) in blocking buffer at 37°C for 1 h. The cells were then photographed under a Leica DMI8 microscope after being washed twice with PBS. The primary antibodies used were FLAG (F1804, Sigma, 1:500) and TFCP2L1 (AF5726, R&D Systems, 1:500).

### Cell proliferation assay

Breast cancer cells were seeded at a density of 2000 cells per well in 96-well plates, and each experiment was performed in quintuplicate. After the cells were seeded for 4 h, the Cell Counting Kit-8 (CCK8) reagent (K1018, APE×BIO Biotechnology) was prepared and used according to the manufacturer’s protocol. After incubation for 1 h at 37°C in the dark, the absorbance at 450 nm was measured using a microplate reader (SpectraMax M5, Molecular Devices, USA). The CCK8 reagent was subsequently added every 12 h for further measurements.

### Transwell assay

Cells were dissociated into single cells using trypsin and counted using a cell counter. Either 6×10^4^ or 8×10^4^ cells were suspended in 100 μl of serum-free DMEM and added to the upper chamber, while 600 μl of fresh culture medium containing 10% fetal bovine serum was added to the lower chamber. Following incubation at 37°C for 12–24 h, the cells on the upper side of the membrane were gently removed with a cotton swab, while the migrated cells on the lower side were fixed with 4% paraformaldehyde and stained with 0.5% crystal violet (C8470, Solarbio, China) for 30 min. The cells adhering to the membrane were imaged using a Leica DMI8 microscope and counted using ImageJ software.

### Wound-healing assay

First, 6 × 10^5^ cells were seeded into six-well plates. Scratch wounds were created via the use of a 10 μl pipette tip, when the cell density reached 85%−90%. The cells were subsequently washed with PBS and were maintained in the culture medium containing 2% FBS. The appropriate scratch area was then selected under a microscope for image acquisition. Wound healing was monitored at 0 h, 12 h and 24 h, and the healing area was quantified using ImageJ software. Each experiment was performed in triplicate.

### Colony formation assay

Cells were seeded into six-well plates at approximately 400–500 cells per well and cultured for 2 weeks. The cell colonies were fixed with 4% paraformaldehyde and stained with 0.5% crystal violet (C8470, Solarbio) for 30 min. After being washed three times with PBS, the colonies were counted using ImageJ after being photographed. Each experiment was performed in triplicate.

### Phalloidin cytoskeleton staining assay

Cells were washed twice with PBS and then fixed with 4% paraformaldehyde at room temperature for 20 min. After being washed with PBS, the cell membrane was perforated with 0.1% Triton X-100. Next, the cells were incubated with the prepared Actin-Tracker Green-488 solution (C2201S, Beyotime Biotechnology) at room temperature for 60 min. The cells were gently rinsed with PBS 3 times to remove excess dye before imaging.

### Animal models

Five-week-old female BALB/c mice were purchased from Henan Skobes Biotechnology Co., Ltd. (Henan, China). For the lung metastasis experiments, briefly, the mice were injected with 2 × 10^6^ cells carrying luciferase coding sequences via the tail vein. After 12–15 days, a mixture of anesthetics and D-fluorescein potassium salt (150 mg/kg) (40902es02, Yeasen, China) at a 1:1 ratio was injected into the abdominal cavity of the mice. The mice were then placed in a Tanon-5200Multi chemiluminescence gel imaging system (Shanghai Tanon, China) to measure fluorescence intensity. For the subcutaneous tumorigenesis experiment, the mice were injected with 2×10^6^ cells carrying the luciferase coding sequence subcutaneously. Randomization was employed along with single blinding during measurements.

### Immunohistochemistry

The paired samples of breast cancer and adjacent normal tissues were obtained from the Pathology Center of the First Affiliated Hospital of the University of Science and Technology of China. Immunohistochemistry is performed according to standard experimental procedures. Briefly, immunohistochemistry was performed on 4-μm-thick formalin-fixed paraffin-embedded tissue sections. A well-validated rabbit monoclonal TFCP2L1 antibody (UPA02723, General Biol, China) was used at a dilution ratio of 1:200 and incubated for 60 min at room temperature.

### Detection of intracellular ROS

An ROS assay kit (S0033M, Beyotime Biotechnology) was used. Briefly, the cells were incubated with 2,7-dichlorofluorescein diacetate (DCFH-DA, 1:1000) and Hoechst 33342 (H3570, Invitrogen, 1:10000) in a dark incubator at 37°C for 30 min to determine the intracellular ROS levels. The cells were subsequently washed twice with PBS. The level of ROS in the cells was visualized using a fluorescence microscopy.

### CUT&Tag assay

Cells were suspended in cold DPBS and counted using a cell counter. The CUT&Tag experiment was performed using the CUT&Tag Kit (TD903, Vazyme, China). DNA fragments were precipitated using a FLAG antibody (F1804, Sigma, 1:100). A DNA library was prepared using the TruePrep Index Kit V2 for Illumina (TD202, Vazyme). Sequence information was analyzed using high-throughput sequencing. The screen shots of peak enrichment were analyzed by IGV (version 2.12.3).

### ChIP assay

ChIP experiments were conducted using a ChIP assay kit (P2078, Beyotime Biotechnology) according to the manufacturer’s instruction. A FLAG antibody was used for immunoprecipitation, while IgG served as a negative control. The enrichment levels were determined via RT‒qPCR. The primer sequences and sites within the promoter regions of GPX4 are provided in Table S4.

### Luciferase assay

The promoter sequence of GPX4 (-3000 to +1) was inserted into the pGL6 plasmid to generate pGL6‒GPX4. Both wild-type (WT) and mutant (Mut) pGL6‒GPX4 plasmids were cotransfected into breast cancer cells along with a *Renilla* luciferase plasmid and *TFCP2L1*-overexpressing constructs. Luciferase activity was assessed using the TransDetect® Double-Luciferase Reporter Assay Kit (FR201, Trans-Gen Biotech, China) after 48 h.

### Accession number

Our RNA-sequencing data in this study were deposited in the Gene Expression Omnibus of NCBI with the accession code GSE270578.

### Statistical analysis

The number of biological replicates is indicated in each legend. All data are reported as the mean ± SD. The data was visualized with GraphPad Prism 8. Unpaired, two-tailed Student’s *t*-tests were employed for comparisons between two groups, and one-way or two-way ANOVA with Sidak’s multiple comparisons test was utilized for comparisons among multiple groups. A *P*-value of less than 0.05 was considered statistically significant.

## Supplementary information

Supplementary Information

Supplementary Information Tables S1-4

Original Western Blots

## Data Availability

The data that support the findings of this study are available from the corresponding author upon reasonable request.
