# Supplementary material for: TFCP2L1 suppresses breast cancer progression by promoting ferroptosis through direct and PI3K/AKT-mediated inhibition of GPX4
Source: Cell Death Discov. 2026 Jun 26;12:364. doi: 10.1038/s41420-026-03231-7 (PMC13562584; doi:10.1038/s41420-026-03231-7)
Supplement: Supplementary file 1 [file 41420_2026_3231_MOESM1_ESM.pdf]

A

Parameter	T1	T2	T3	T4	T5	T6
Age	31	56	46	59	38	41
Size (cm)	2×1.5×1	2.5×2.2×2.2	1.5×1.5×1	1×1×1	1.1×1.1×1.1	2.5×2×1.5
ER/PR/Her2	ER+/PR+/Her2+	ER+/PR-/Her2+	ER-/PR-/Her2-	ER-/PR-/Her2-	ER-/PR-/Her2-	ER-/PR-/Her2-
Ki67 Score	+, ~20%	+, ~60%	+, ~70%	+, ~30%	+, ~70%	+, ~80%
Grade	2	3	3	2	4	3

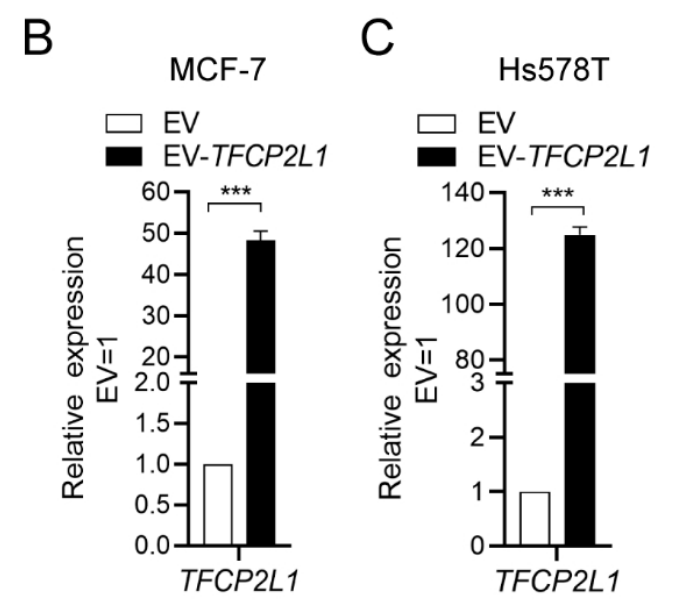

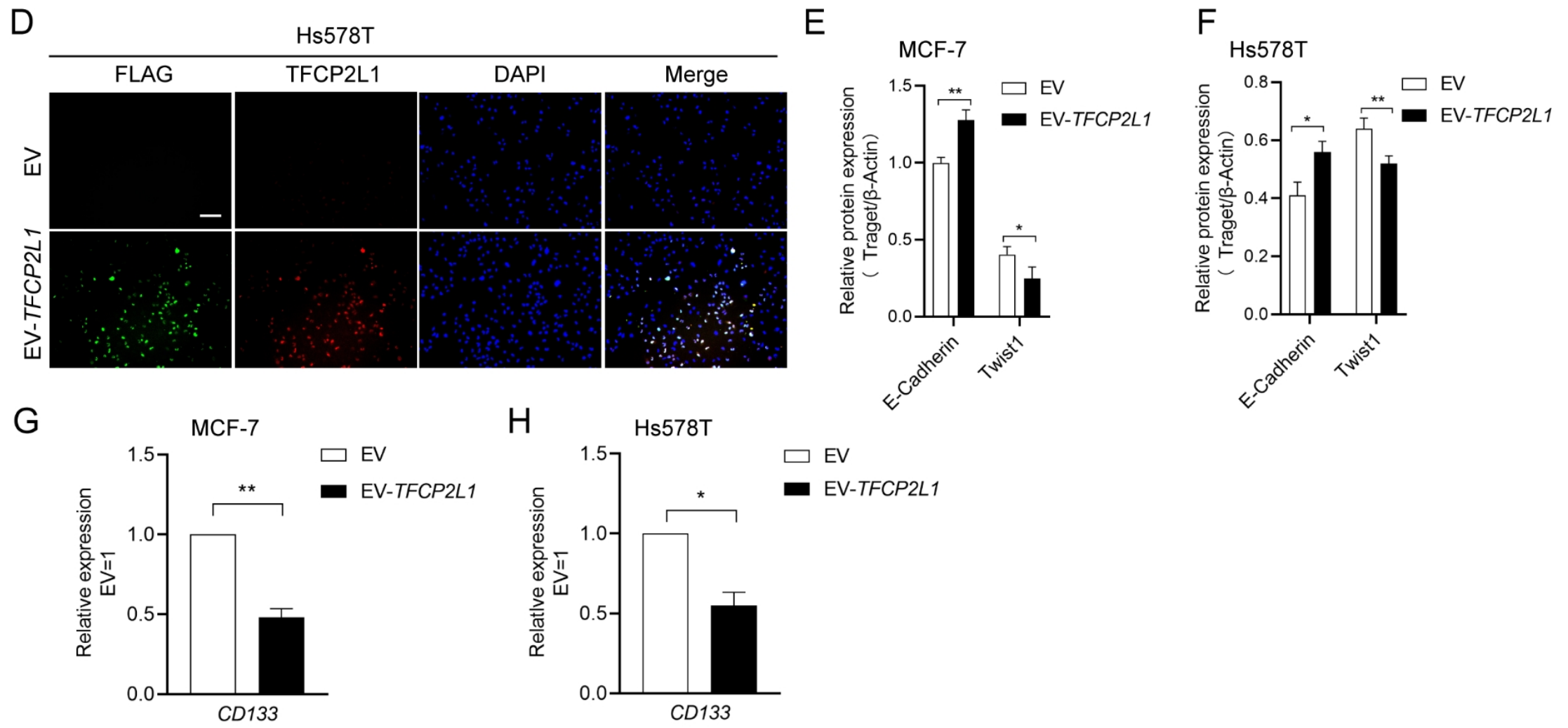

Figure S1. Overexpression of *TFCP2L1* inhibits the progression of breast cancer cells. A) Relevant information on six breast cancer patients (n=6). B,C) RT–qPCR analysis of the expression of *TFCP2L1* in EV- and EV-*TFCP2L1*-transfected MCF-7 and Hs578T cells (n=3). D) Immunofluorescence staining for FLAG and TFCP2L1 in EV- and EV-*TFCP2L1*-transfected Hs578T cells. Bar, 50 µm. E,F) Densitometric analysis of the relative protein levels of E-Cadherin and Twist1, as shown in Figure 2J and K, was performed with ImageJ software. Protein levels were normalized to those of β-Actin (n=3). G,H) RT-qPCR was used to detect *CD133* expression in MCF-7 and Hs578T cells expressing *EV* or *EV-TFCP2L1*, which were cultured in suspension to form spheres (n=3). The data are presented as mean ± SD. Statistical analyses were performed using Student's *t*-tests (B,C,E,F,G,H). *$p < 0.05$, **$p < 0.01$, ***$p < 0.001$.

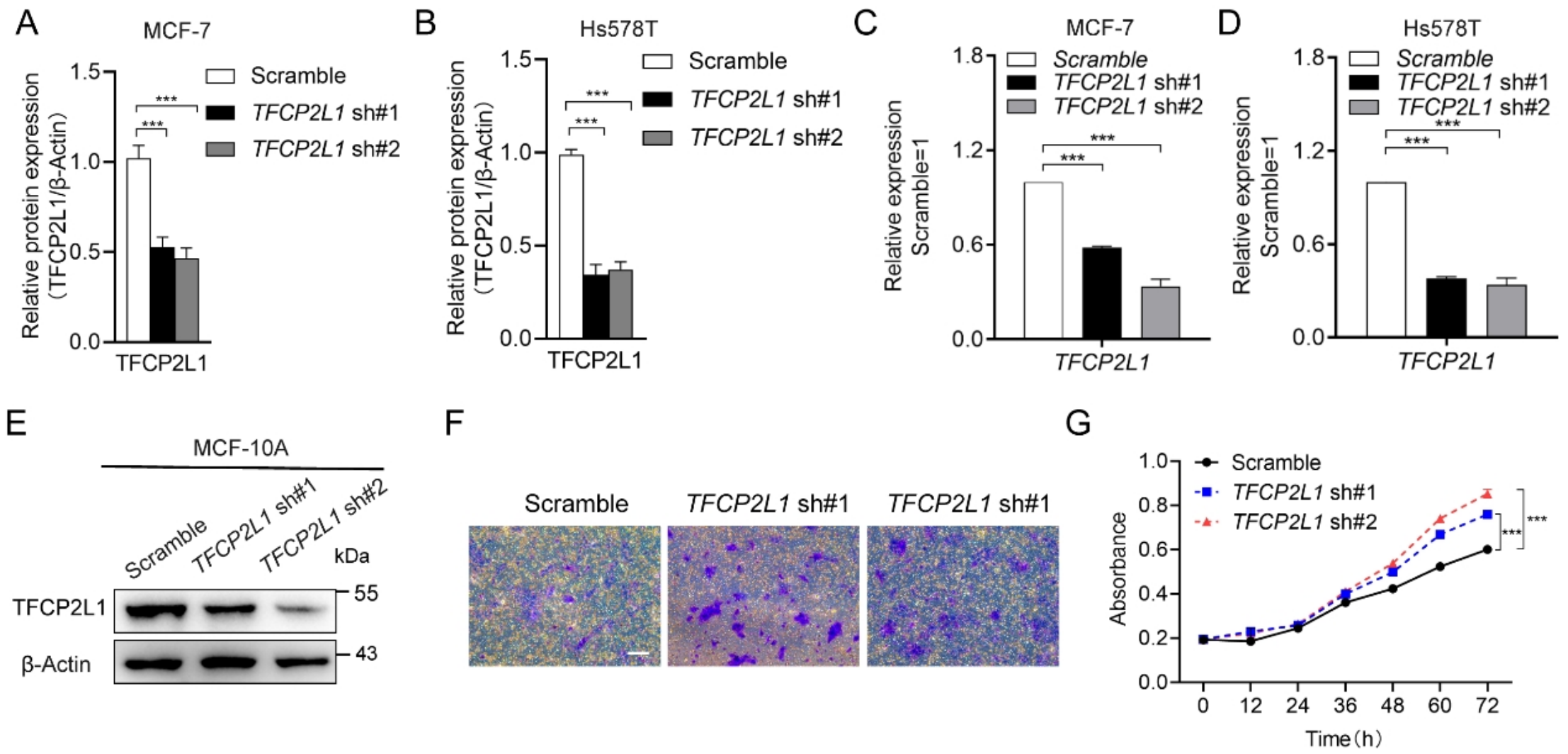

Figure S2. Overexpression and knockdown of *TFCP2L1* regulates the aggressiveness of breast cancer cells. A,B) Densitometric analysis of the relative protein levels of TFCP2L1 as shown in Figure 3A and B, was performed with ImageJ software. Protein levels were normalized to those of β-Actin (n=3). C,D) RT–qPCR analysis of the expression of *TFCP2L1* in *scramble* and *TFCP2L1* shRNA lentivirus-infected MCF-7 and Hs578T cells (n=3). E) Western blot analysis of TFCP2L1 and β-Actin protein levels in scramble and *TFCP2L1* shRNA lentiviruses infected MCF-10A cells. F,G) Transwell (F) and CCK8 (G) assays of the migration of scramble and *TFCP2L1* shRNA-expressing MCF-10A cells (n=3). Bar, 50 µm. The data are presented as mean ± SD. Statistical analyses were performed using one-way ANOVA (A–D) and two-way ANOVA (G) . $^{**}p < 0.01$, $^{***}p < 0.001$.

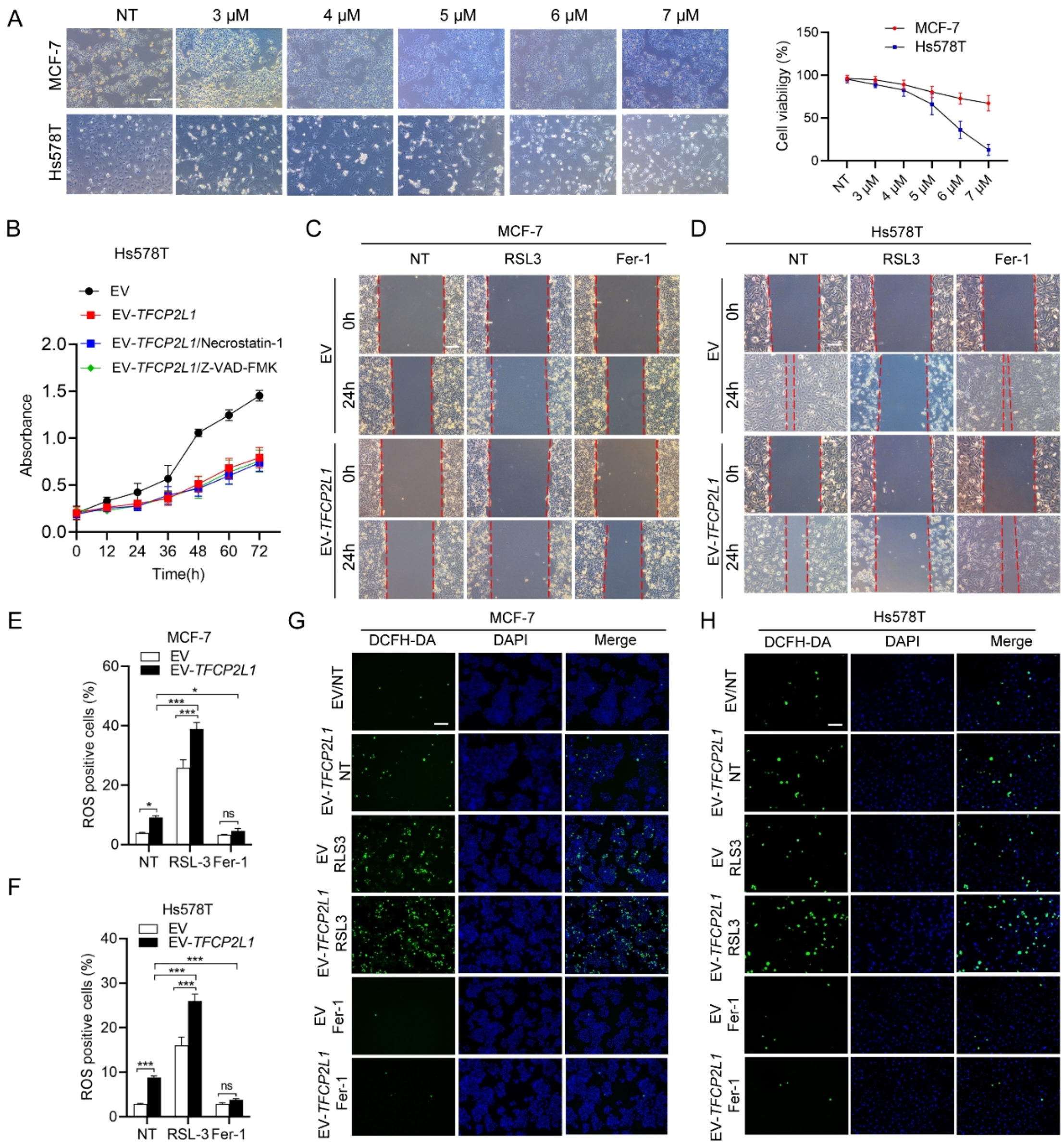

Figure S3. Overexpression of *TFCP2L1* stimulates ferroptosis to inhibit breast cancer cell metastasis. A) Morphology of MCF-7 and Hs578T cells treated with different concentrations of RSL3. The inhibitory effect was measured by CCK8 assay and cytotoxicity was observed. B) CCK8 assay analysis of the proliferation of Hs578T cells transfected with EV or EV-*TFCP2L1* in the presence or absence of Z-VAD-FMK or Necrostatin-1 (n=3). C,D)Wound healing analysis of the migration of EV- and EV-*TFCP2L1*-transfected MCF-7 cells treated with or without 5 μM Fer-1 or 5 μM RSL-3. Bar, 50 μm. E,F) Quantification of results of FACS in Figure 5F and 5G (n=3). G,H) Immunofluorescence staining for ROS intensity in EV- and EV-*TFCP2L1*-transfected MCF-7 or Hs578T cells treated with or without Fer-1 and RSL-3. Bar, 50 μm. The data are presented as mean ± SD. Statistical analyses were performed using two-way ANOVA (E,F). *$p < 0.05$, ***$p < 0.001$. ns, not significant.

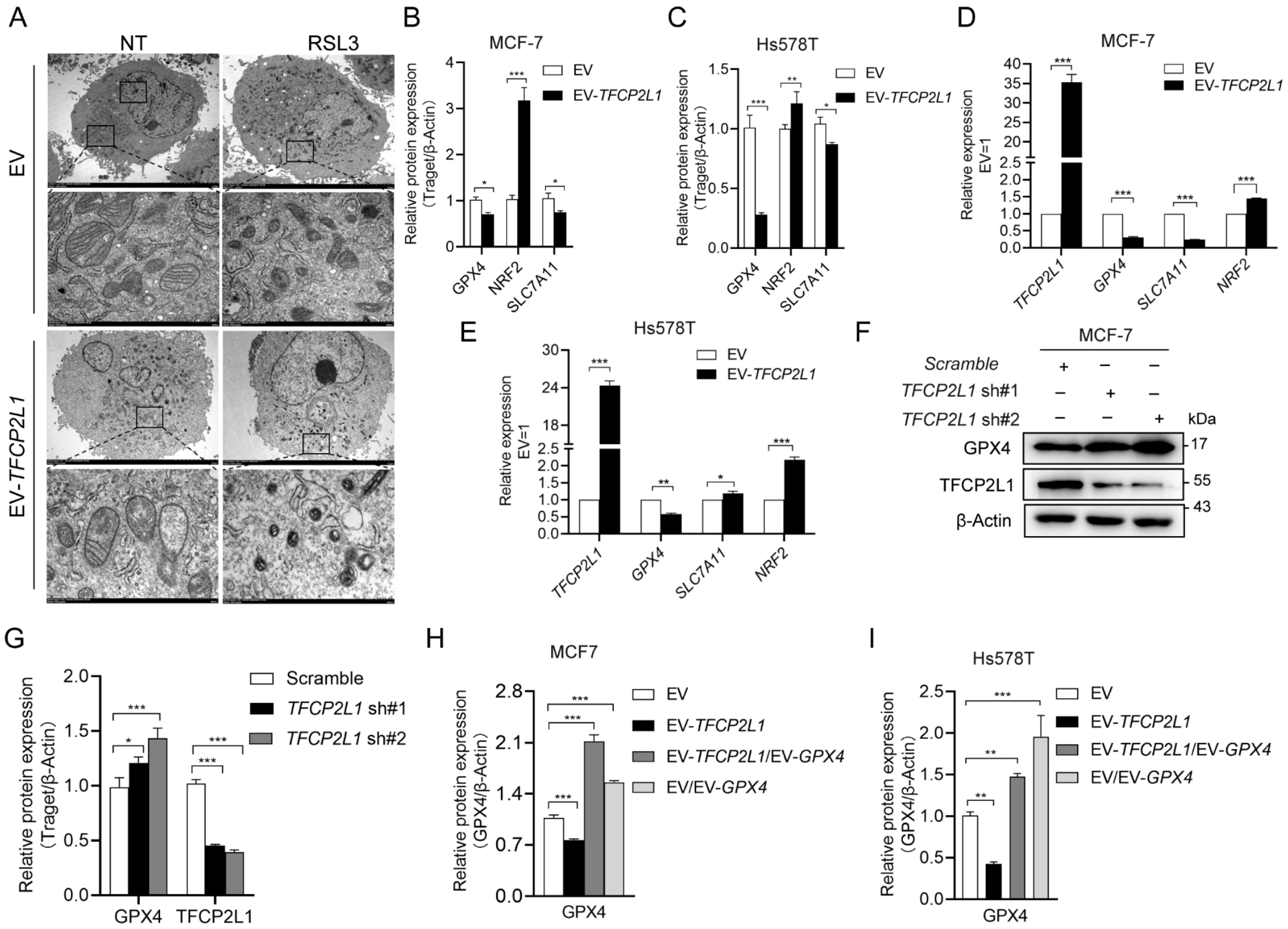

Figure S4. *TFCP2L1* inhibits *GPX4* expression in breast cancer cells. A) The mitochondrial morphology in MCF-7 cells expressing EV or EV-*TFCP2L1* under electron microscopy in the presence or absence of RSL(up, ×3000; down, ×20000). B,C) Densitometric analysis of the relative protein levels of GPX4, NRF2 and SLC7A11, as shown in Figure 6A and B, was performed with ImageJ software. Protein levels were normalized to those of β-Actin (n=3). D,E) RT–qPCR analysis of *TFCP2L1*, *GPX4*, *SCL7A11*, and *NRF2* in EV- and EV-*TFCP2L1*-transfected MCF-7 and Hs578T cells (n=3) . F) Western blot analysis of GPX4 protein levels in scramble and *TFCP2L1* shRNA lentivirus-infected MCF-7 cells. G) Densitometric analysis of the relative protein levels of GPX4 and TFCP2L1, as shown in Figure S4F, was performed with ImageJ software. Protein levels were normalized to those of β-Actin (n=3). H,I) Densitometric analysis of the relative protein levels of GPX4 as shown in Figure 7A and B, was performed with ImageJ software. Protein levels were normalized to those of β-Actin (n=3). Statistical analyses were performed using Student's *t*-tests (B-E) and one-way ANOVA (G-I) . *$p < 0.05$, **$p < 0.01$, ***$p < 0.001$.

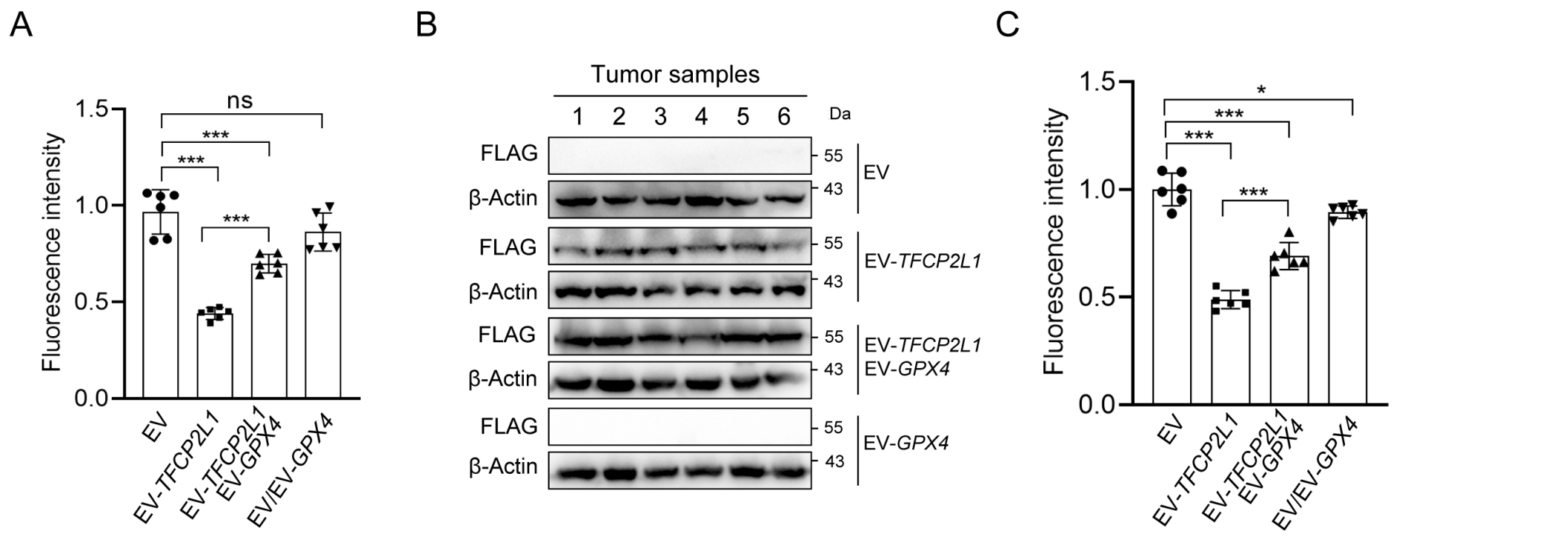

Figure S5. Overexpression of GPX4 impairs the function of *TFCP2L1*. A) Quantitative analysis of the fluorescence intensity in different tumors, as shown in Figure 7F, was performed using ImageJ software (n=6). B) Western blot analysis of FLAG in different dissected tumors, as shown in Figure 7G. C) Quantitative analysis of the fluorescence intensity in different tumors, as shown in Figure 7I, was performed using ImageJ software (n=6). The data are presented as mean ± SD. Statistical analyses were performed using one-way ANOVA (A,C). $*p < 0.05$, $***p < 0.001$.

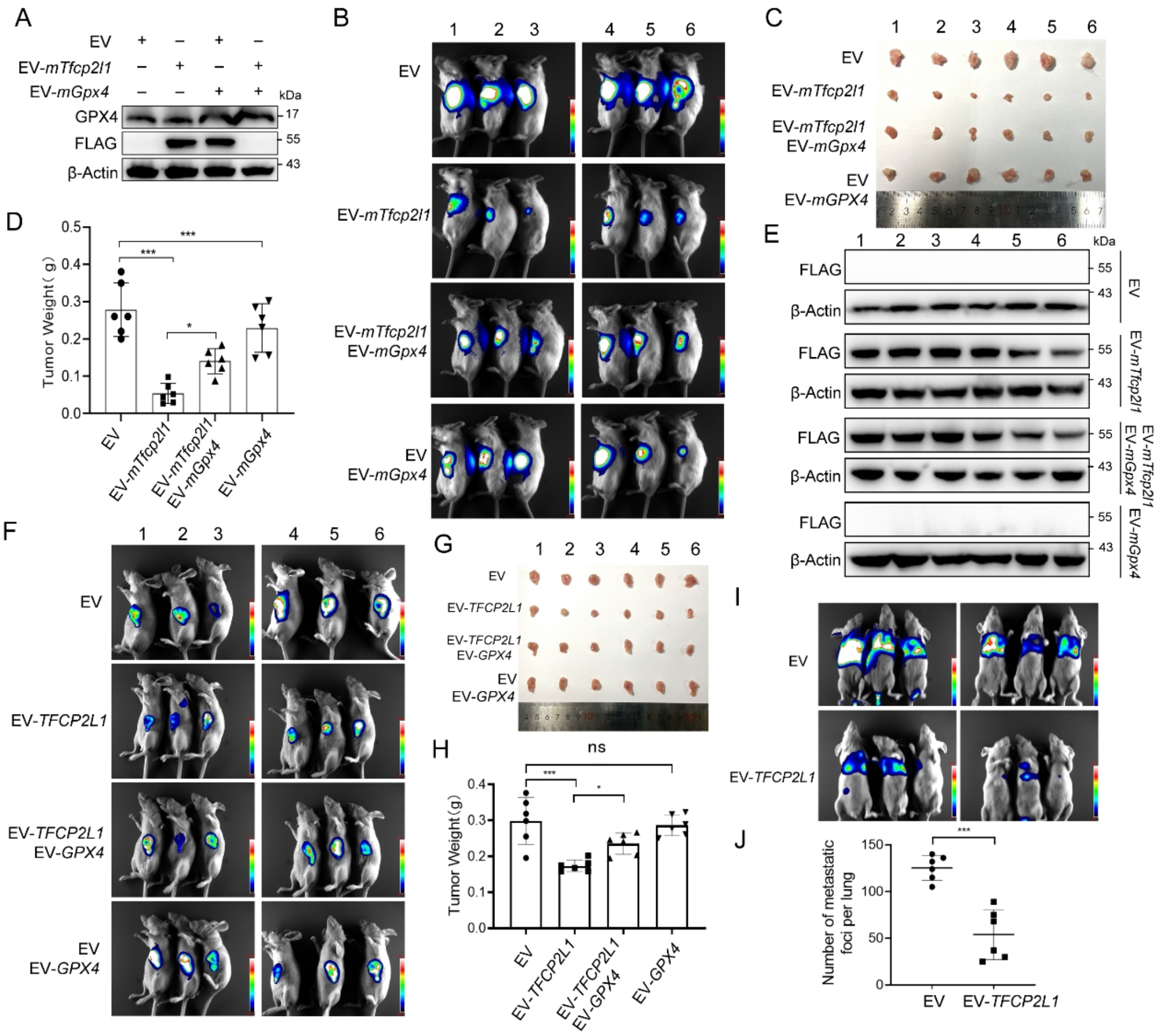

Figure S6. Overexpression of mouse *Gpx4* mediates the function of mouse *Tfcp2l1* in the 4T1 cell line. A) Western blot analysis of FLAG and GPX4 in 4T1 cells transfected with an EV, FLAG-tagged mouse *Tfcp2l1*-overexpressiing plasmid (EV-m*Tfcp2l1)* or/and a mouse *Gpx4*-overexpressing plasmid without a tag (EV-m*GPX4)*. B) Representative bioluminescence images of mice injected subcutaneously with luciferase-labeled 4T1 cells transfected with EV, EV-m*Tfcp2l1* or/and EV-m*GPX4*. C) Dissected tumors from the mice in Figure S6B. D) Comparison of the average weights of the tumors in the different groups shown in Figure S6C (n=6). E) Western blot analysis of the FLAG in different dissected tumors, as shown in Figure S6C. F) Representative bioluminescence images of NOD mice injected subcutaneously with luciferase-labeled Hs578T cells transfected with EV, EV-*TFCP2L1* or/and EV-*GPX4*. G) Dissected tumors from the mice in Figure S6F. H) Comparison of the average weights of the tumors in the different groups shown in Figure S6F (n=6). I) Injection of EV- and EV-*TFCP2L1*-transfected Hs578T cells into NOD mice via tail vein. Luciferase signals were captured via a Tanon-5200Multi system. J) Comparison of the average number of metastatic nodules in the lungs among different groups of mice (n=6). The data are presented as mean ± SD. Statistical analyses were performed using one-way ANOVA (D,H) and Student's *t*-tests (J). *$p < 0.05$, ***$p < 0.001$.

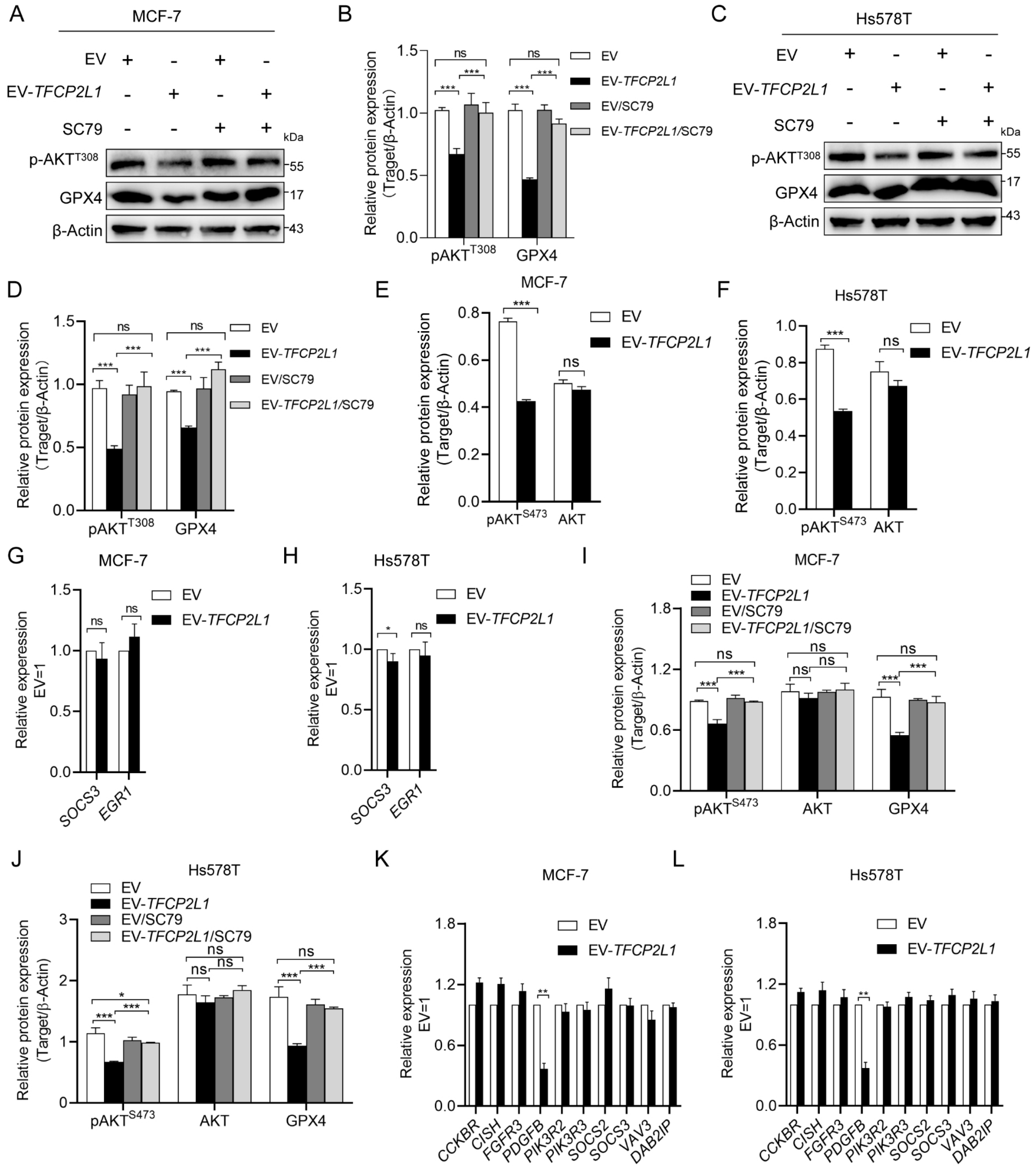

Figure S7. The activation of AKT affects the expression of GPX4 regulated by TFCP2L1. A) Western blot analysis of pAKT^{T308} and GPX4 protein levels in EV- and EV-*TFCP2L1*-overexpressing MCF-7 cells in the presence or absence of SC79. B) Densitometric analysis of the relative protein levels of pAKT^{T308} and GPX4, as shown in Figure S7A, was performed with ImageJ software. Protein levels were normalized to those of β-Actin (n=3). C) Western blot analysis of pAKT^{T308} and GPX4 protein levels in EV- and EV-*TFCP2L1*-overexpressing Hs578T cells in the presence or absence of SC79. D) Densitometric analysis of the relative protein levels of pAKT^{T308} and GPX4, as shown in Figure S7C, was performed with ImageJ software. Protein levels were normalized to those of β-Actin (n=3). E,F) Densitometric analysis of the

relative protein levels of $pAKT^{S473}$ and AKT, as shown in Figure 8C and D, was performed with ImageJ software. Protein levels were normalized to those of β-Actin (n=3). G,H) RT-qPCR analysis of the expression levels of SOCS3 and EGR1 in EV- and EV-*TFCP2L1*-expressing MCF-7 and Hs578T cells (n=3). I,J) Densitometric analysis of the relative protein levels of $pAKT^{S473}$, AKT and GPX4, as shown in Figure 8E and F, was performed with ImageJ software. Protein levels were normalized to those of β-Actin (n=3). K,L) RT-qPCR detected the expression of *CCKBR*, *CISH*, *FGFR3*, *PDGFB*, *PIK3R2*, *PIK3R3*, *SOCS2*, *SOCS3*, *VAV3* and *DAB2IP* in MCF-7 and Hs578T cells expressing EV or EV-*TFCP2L1* (n=3). The data are presented as mean ± SD. Statistical analyses were performed using Student's *t*-tests (E,F,G,H,K,L) and one-way ANOVA (B,D,I,J). *$p < 0.05$, ***$p < 0.001$.
