# Supplementary material for: TFCP2L1 suppresses breast cancer progression by promoting ferroptosis through direct and PI3K/AKT-mediated inhibition of GPX4
Source: Cell Death Discov. 2026 Jun 26;12:364. doi: 10.1038/s41420-026-03231-7 (PMC13562584; doi:10.1038/s41420-026-03231-7)
Supplement: Supplementary file 2 [file 41420_2026_3231_MOESM2_ESM.pdf]

Supplementary Tables

Table S1. Primer sequences for gene overexpression experiment

Symbol	Forward sequence (5'-3')	Reverse sequence (5'-3')
	Homo sapiens(human)	
TFCP2L1	ATGCTCTTCTGGCACACGCA	TCAGAGTCCACATTTCAGGAT
GPX4	ATGAGCCTCGGCCGCCTTTGC	TCATGAGTGCCGGTGGAAGGC

Table S2. Primer sequences for gene knockdown experiment.

Symbol	Forward sequence (5'-3')	Reverse sequence (5'-3')
TFCP2L1 sh#1	CCGGGATCACAGAGATGAA<br>CCGGATCTCGAGATCCGGTT<br>CATCTCTGTGATCTTTTTG	AATTCAAAAAGATCACAGAGA<br>TGAACCGGATCTCGAGATCCG<br>GTTCATCTCTGTGATC
TFCP2L1 sh#2	CCGGAGGTAGCAGTGGAAG<br>CTACTACTCGAGTAGTAGCT<br>TCCACTGCTACCTTTTTTG	AATTCAAAAAAGGTAGCAGTG<br>GAAGCTACTACTCGAGTAGTA<br>GCTTCCACTGCTACCT
TFCP2L1 sh#3	CCGGAGCATGAGATCTCTGA<br>GATGACTCGAGTCATCTCAG<br>AGATCTCATGCTTTTTTG	AATTCAAAAAAGCATGAGATC<br>TCTGAGATGACTCGAGTCATCT<br>CAGAGATCTCATGCT

Table S3. Primer sequences for RT–qPCR analysis

Symbol	Forward sequence (5'-3')	Reverse sequence (5'-3')
TFCP2L1	GTGGGGACTACTCGGAGCAT	AAGCTGTTGGGAGAGCCATT
β-ACTIN	TATCGCTGCGCTGGTCG	CCCACGATGGAGGGGAATAC
E-Cadherin	TGGAGAGACACTGCCAACTG	CTCAAAATCCTCCCTGTCCA
N-Cadherin	GACAATGCCCCTCAAGTGTT	CCATTAAGCCGAGTGATGGT
VIMENTIN	ATGCTCGAGTTGAAGCCTGT	CTCCAGGTCATCGTGGTTCT
GPX4	CCGTCTCTCCACAGTTCCTC	GGGGCAGGTCCTTCTCTATC
NRF2	CGGTATGCAACAGGACATTG	CTGTCAACTGGTTGGGGTCT
SLC7A11	TTTGCACCCTTTGACAATGA	AGCCCAATAAAAAGCCACCT

Table S4. List of primers used for ChIP–RT–qPCR analysis

Location From UCSC	Forward sequence (5’-3’)	Reverse sequence (5’-3’)
-505 ~ -496	CTGGAGGATCACTTGAGGTCA	TCTCTGCTTCCCGAGTAGCTG
-198 ~ -180	ATTCTCAGGTTGTCTGGCCTG	CGCGTCGGGAGGCAGCGCAGT
