# Supplementary material for: TFCP2L1 suppresses breast cancer progression by promoting ferroptosis through direct and PI3K/AKT-mediated inhibition of GPX4
Source: Cell Death Discov. 2026 Jun 26;12:364. doi: 10.1038/s41420-026-03231-7 (PMC13562584; doi:10.1038/s41420-026-03231-7)

Figure 1G

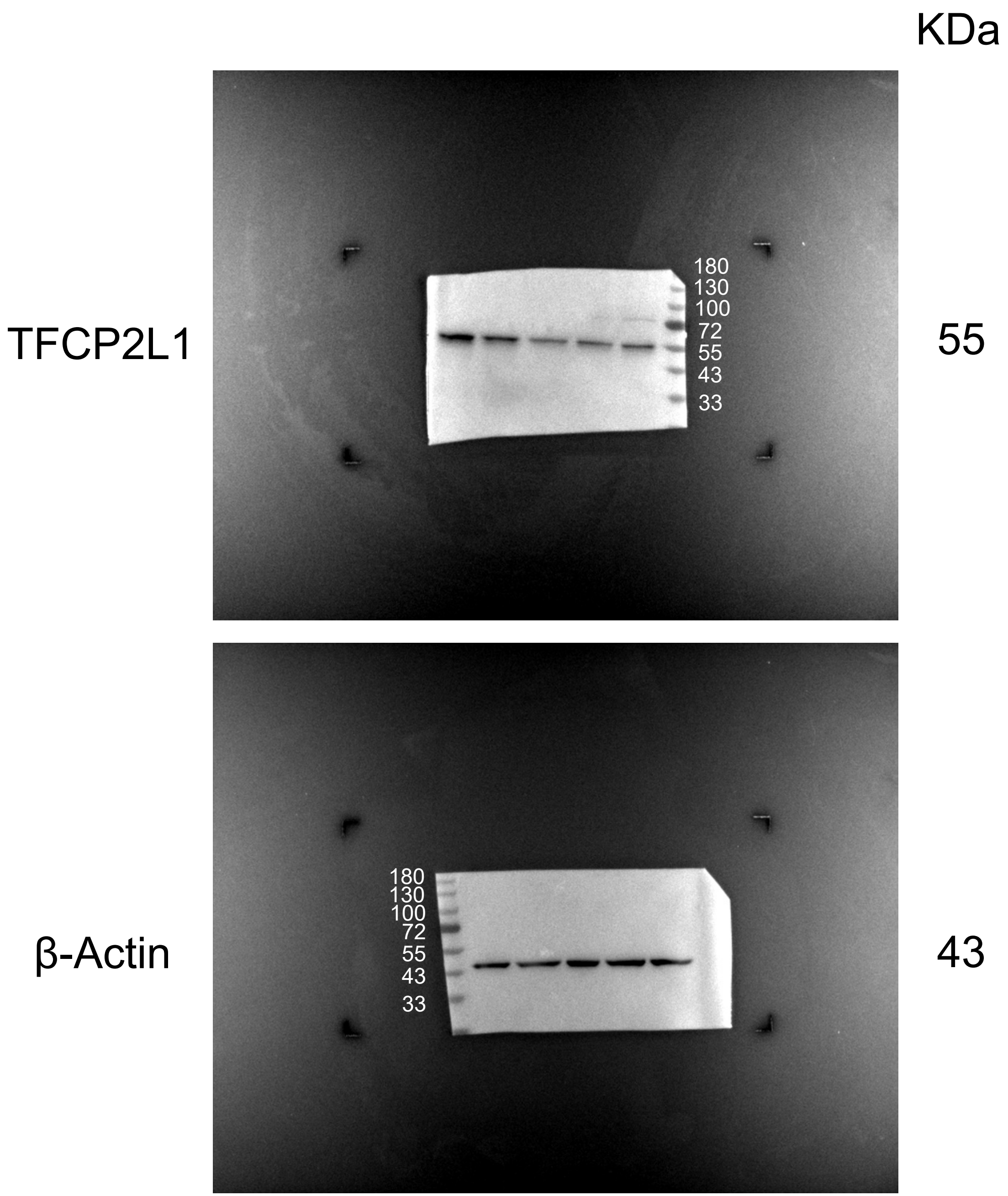
KDa
TFCP2L1
55
180
130
100
72
55
43
33
β-Actin
43
180
130
100
72
55
43
33

Figure 2A

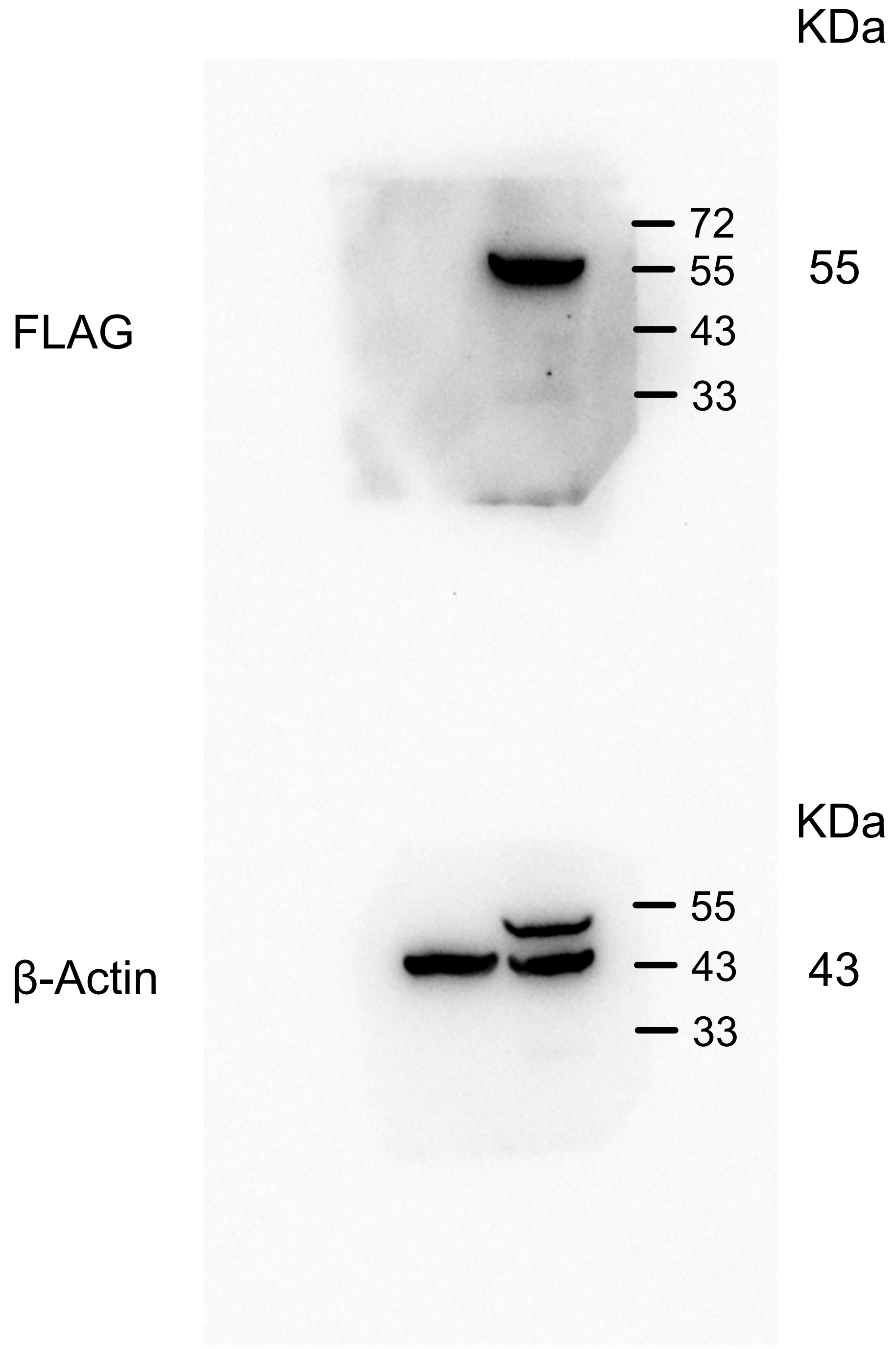

Figure 2B

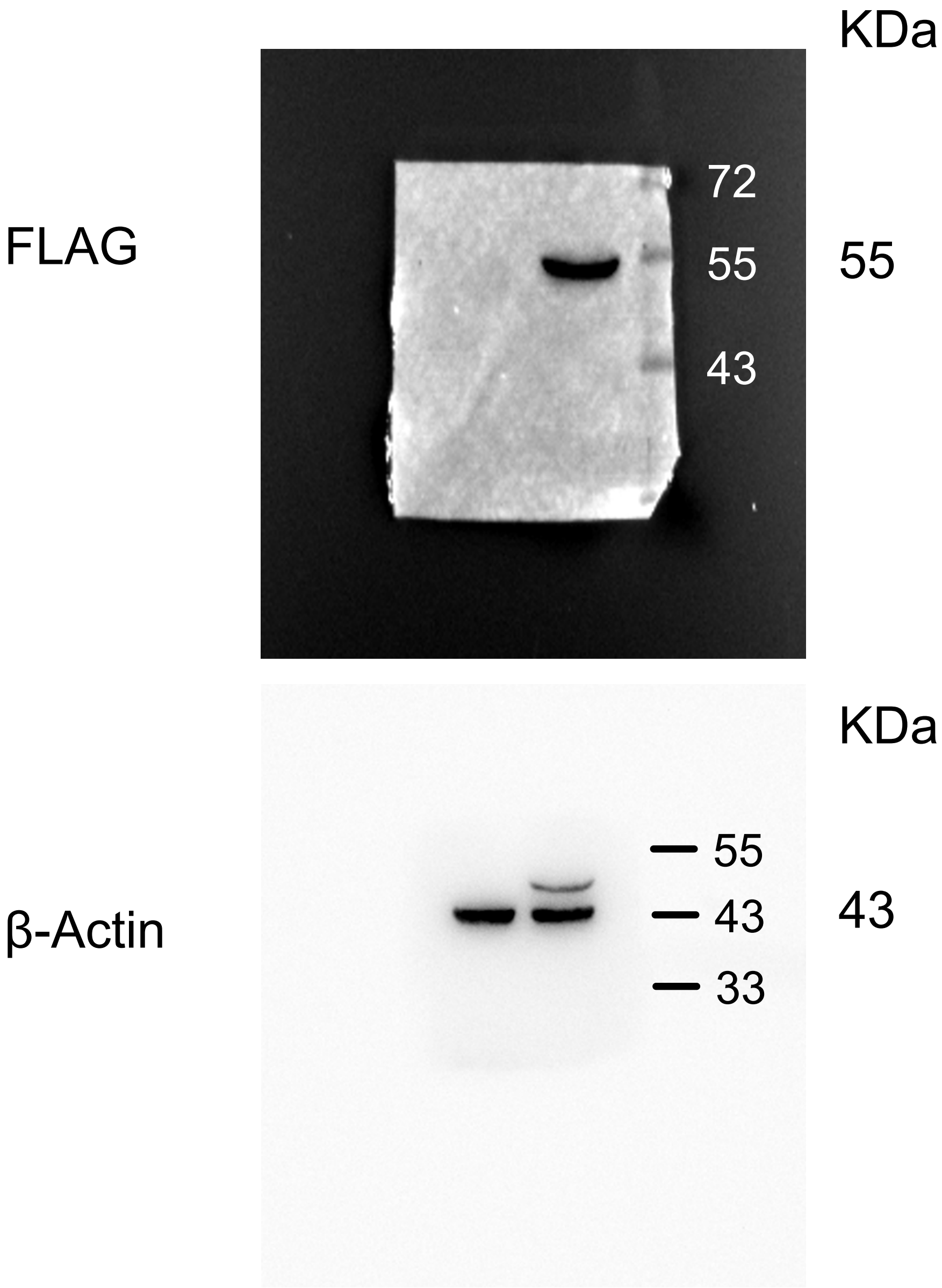

Figure 2J

KDa

E-cadherin 130

TWIST1 37

β-Actin 43

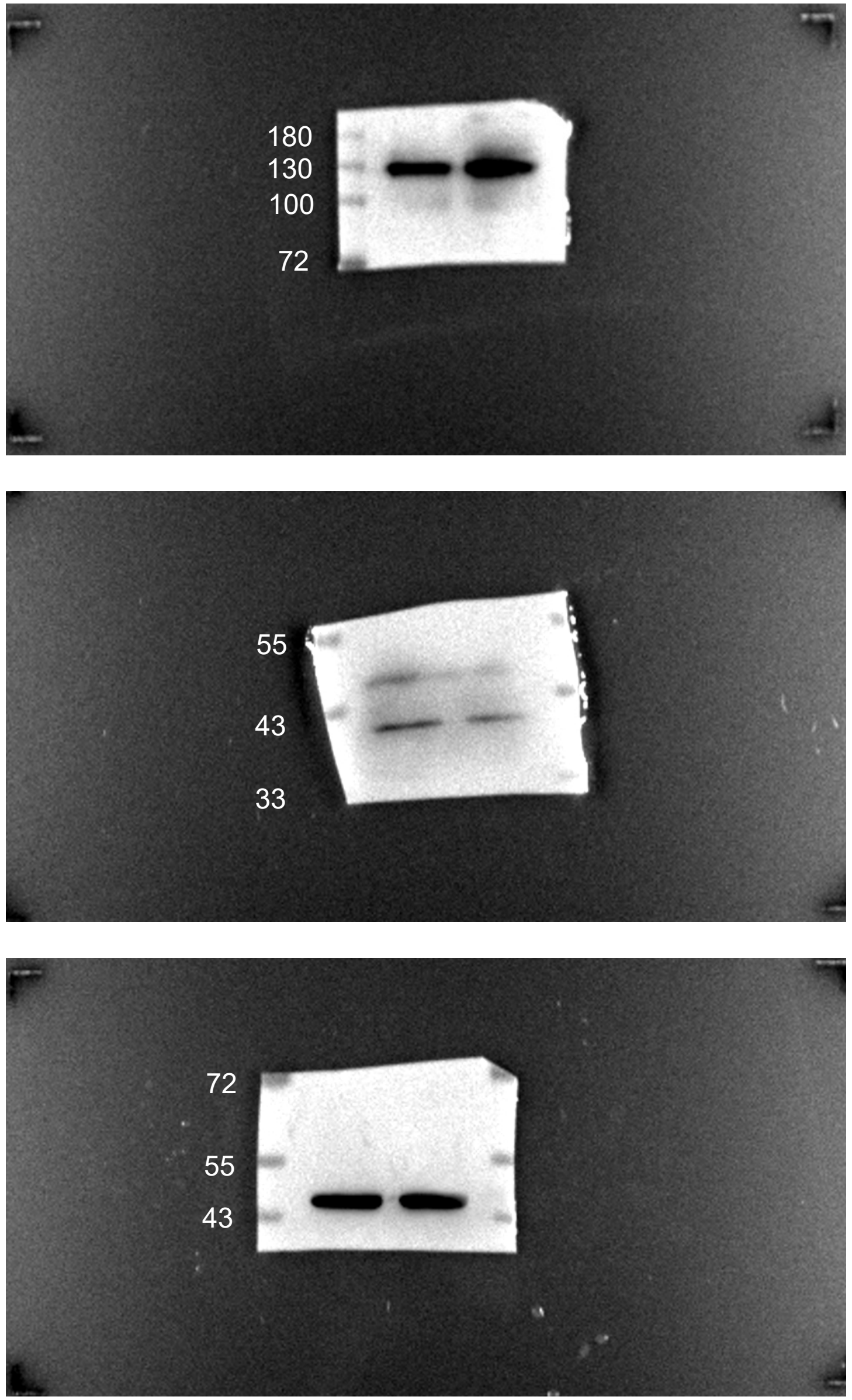

Figure 2K

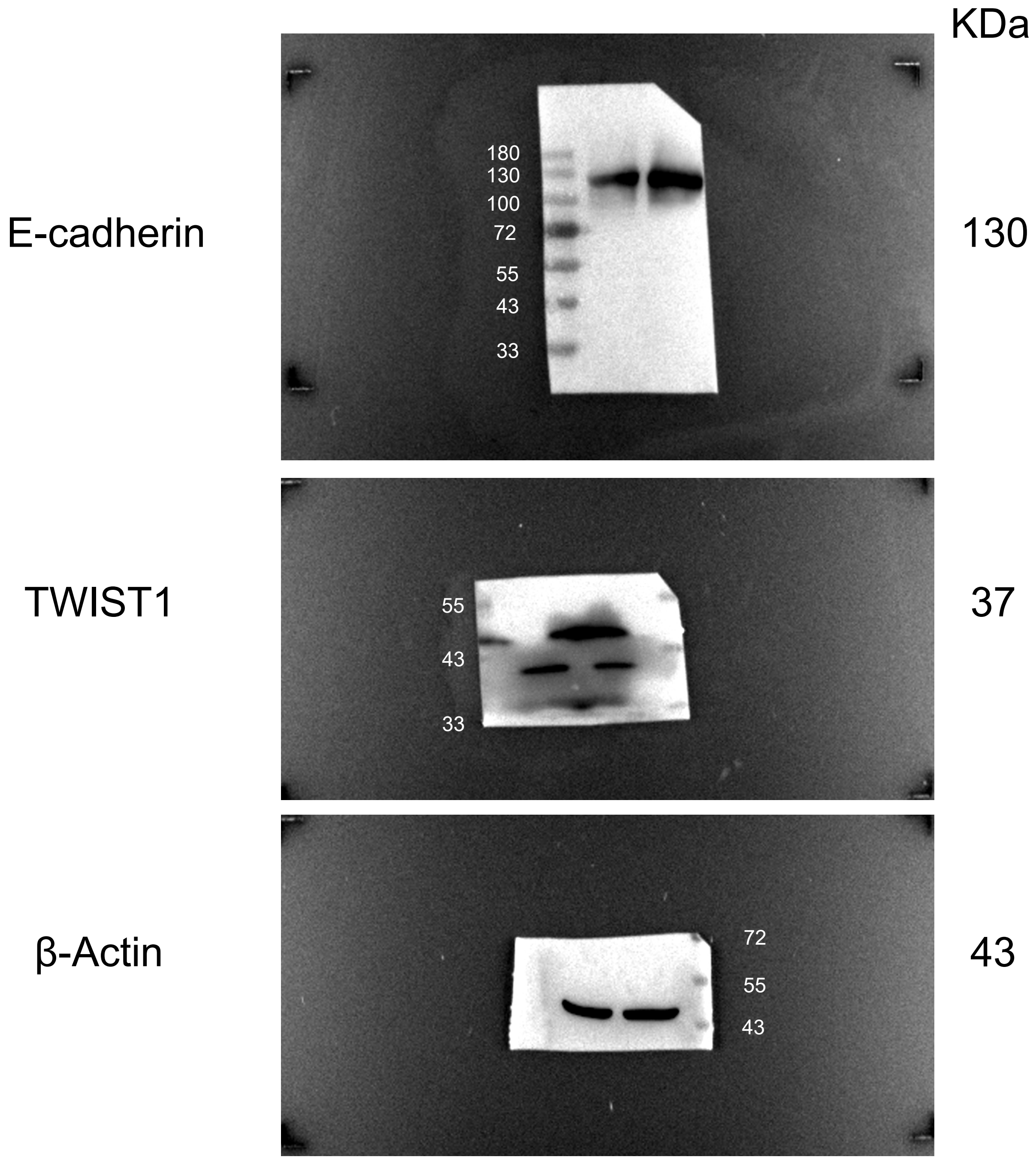
KDa
E-cadherin
180
130
100
72
55
43
33
130
TWIST1
55
43
33
37
β-Actin
72
55
43
43

Figure 3A

KDa

TFCP2L1 55

β-Actin 43

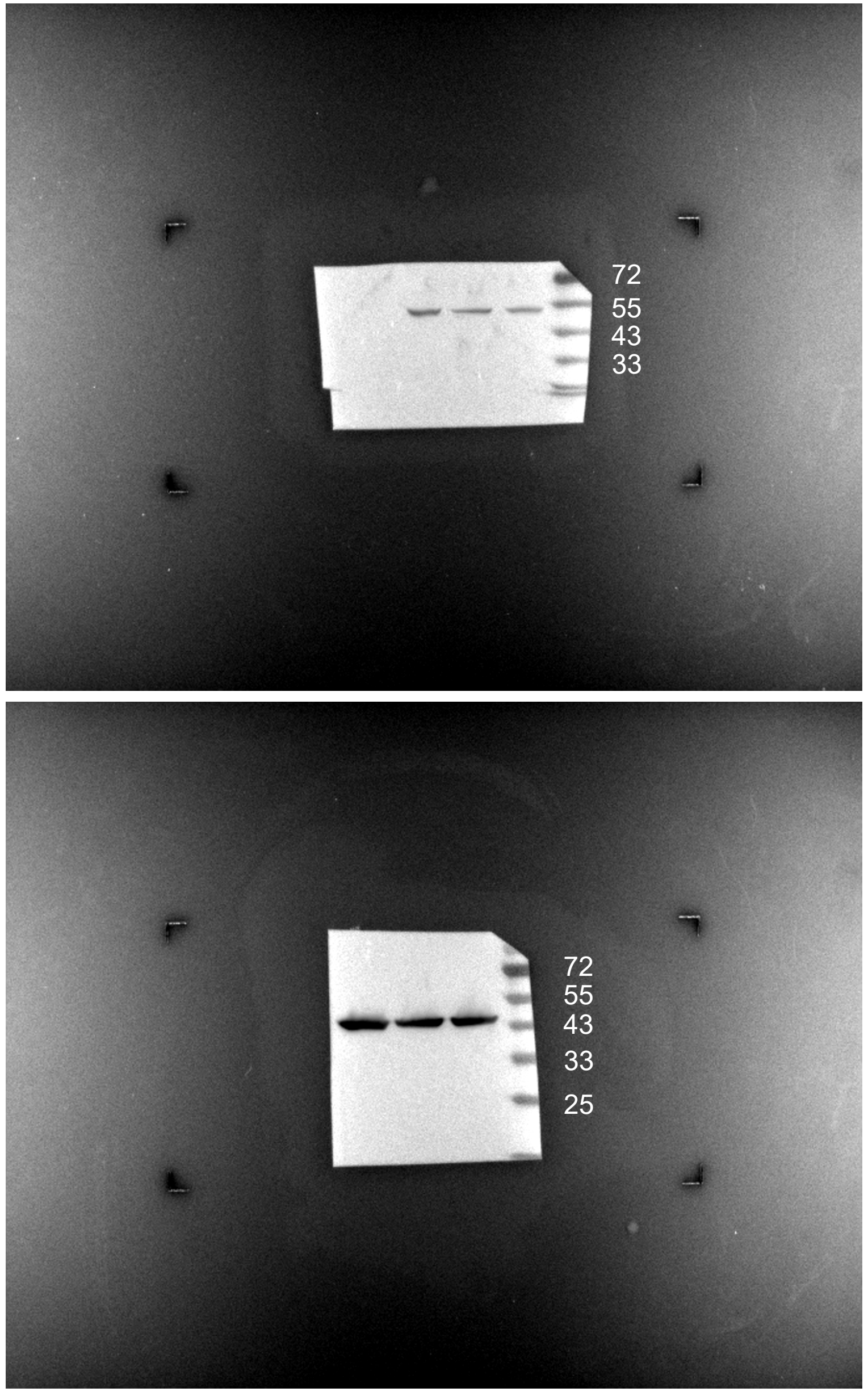

Figure 3B

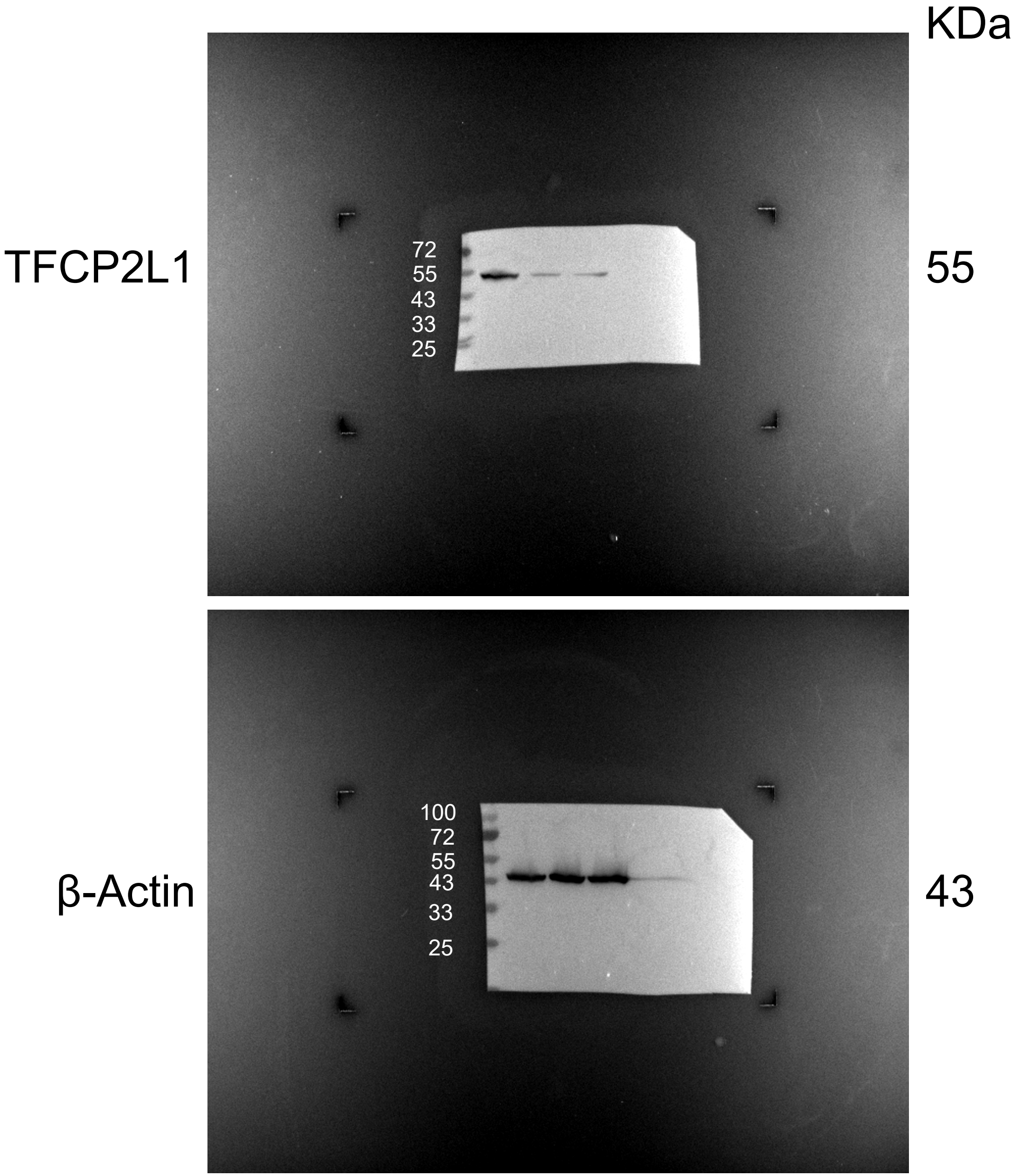

Figure 4A

KDa

FLAG 55

β-Actin 43

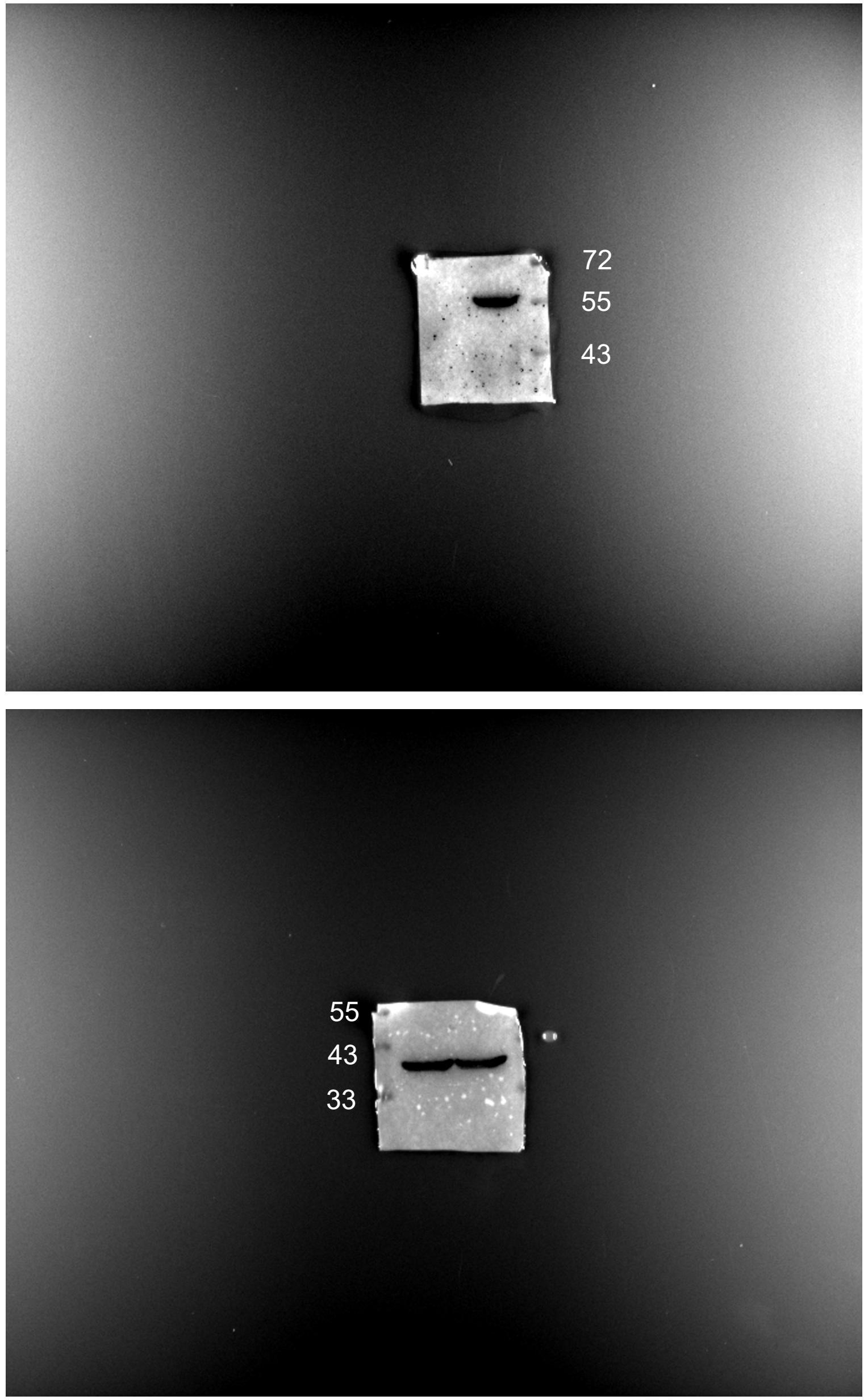

Figure 4D

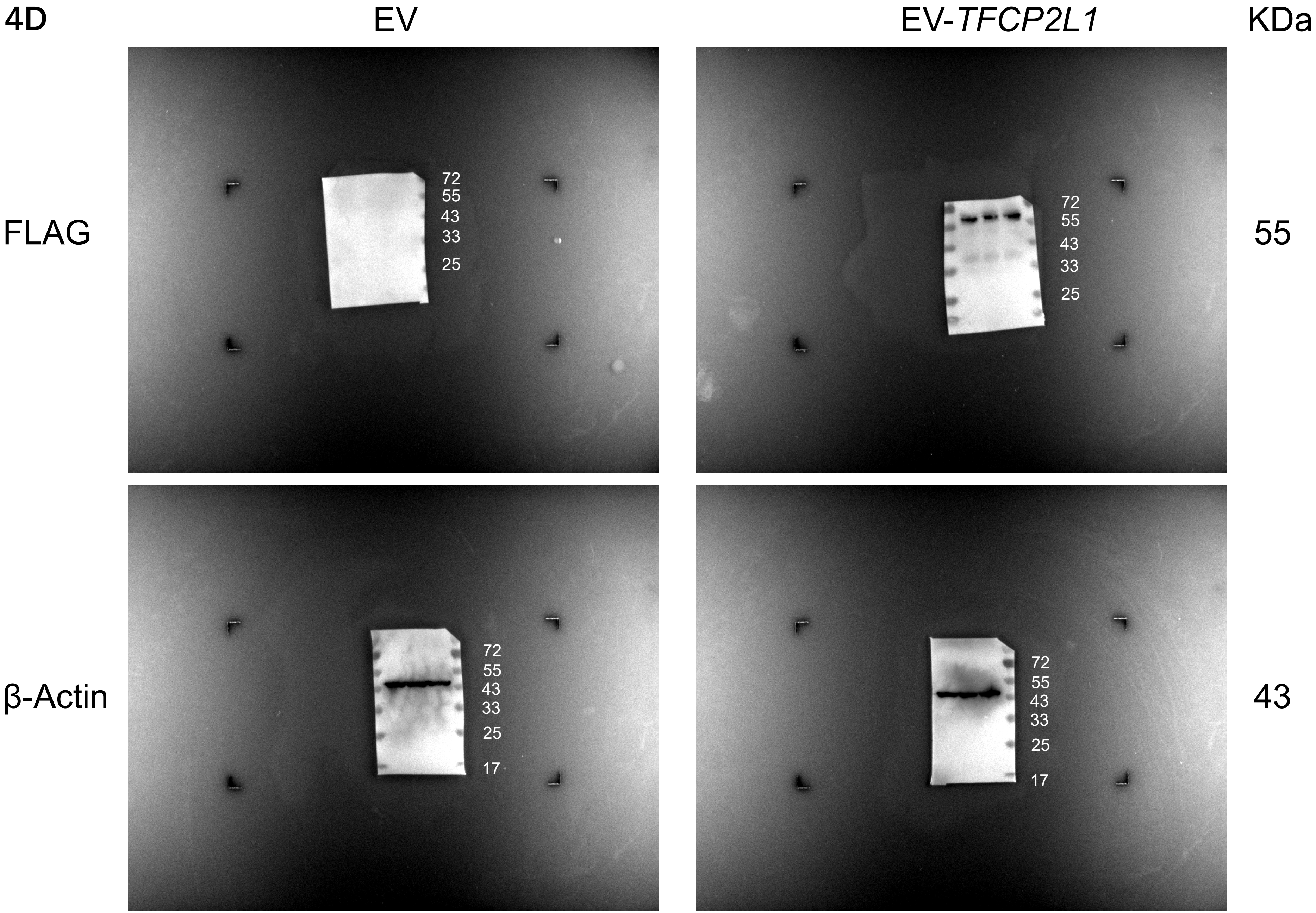

Figure 6A

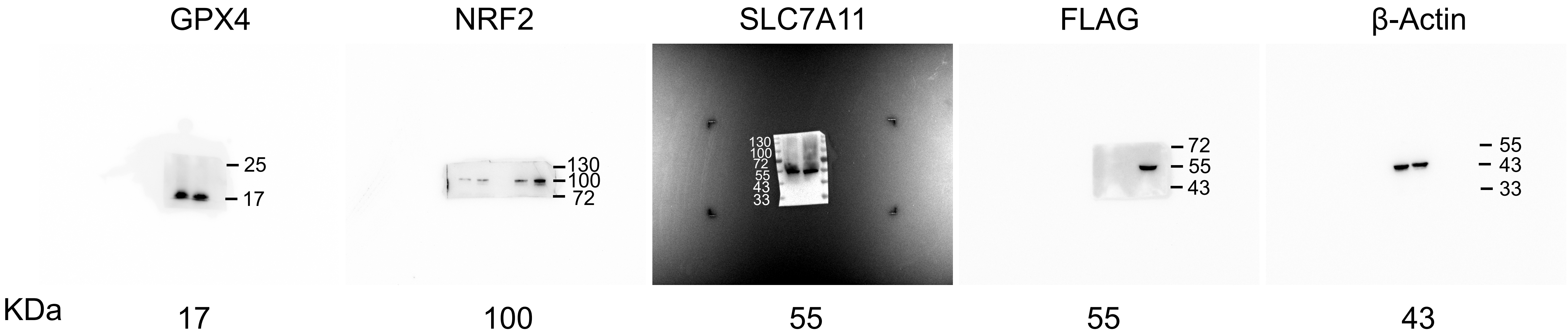
GPX4
NRF2
SLC7A11
FLAG
β-Actin
25
17
130
100
72
130
100
72
55
43
33
72
55
43
55
43
33
KDa
17
100
55
55
43

Figure 6B

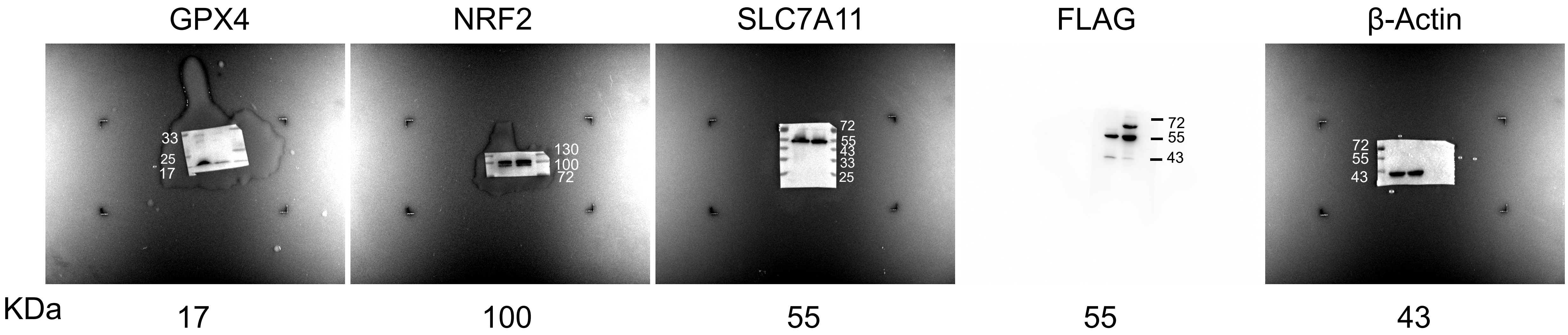
GPX4
NRF2
SLC7A11
FLAG
β-Actin
33
25
17
130
100
72
72
55
43
33
25
72
55
43
72
55
43
KDa
17
100
55
55
43

Figure 7A

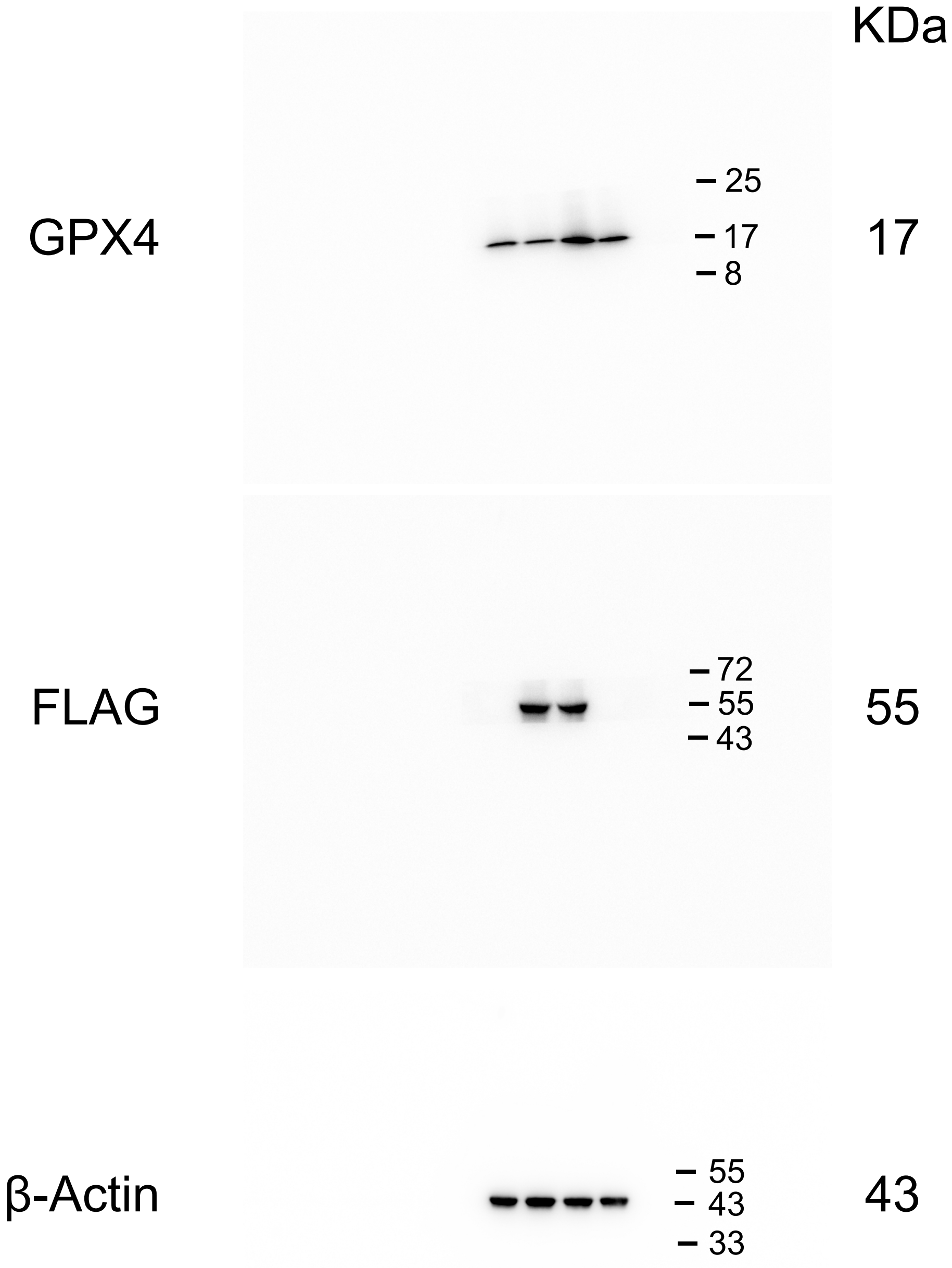

Figure 7B

KDa

GPX4 17

FLAG 55

β-Actin 43

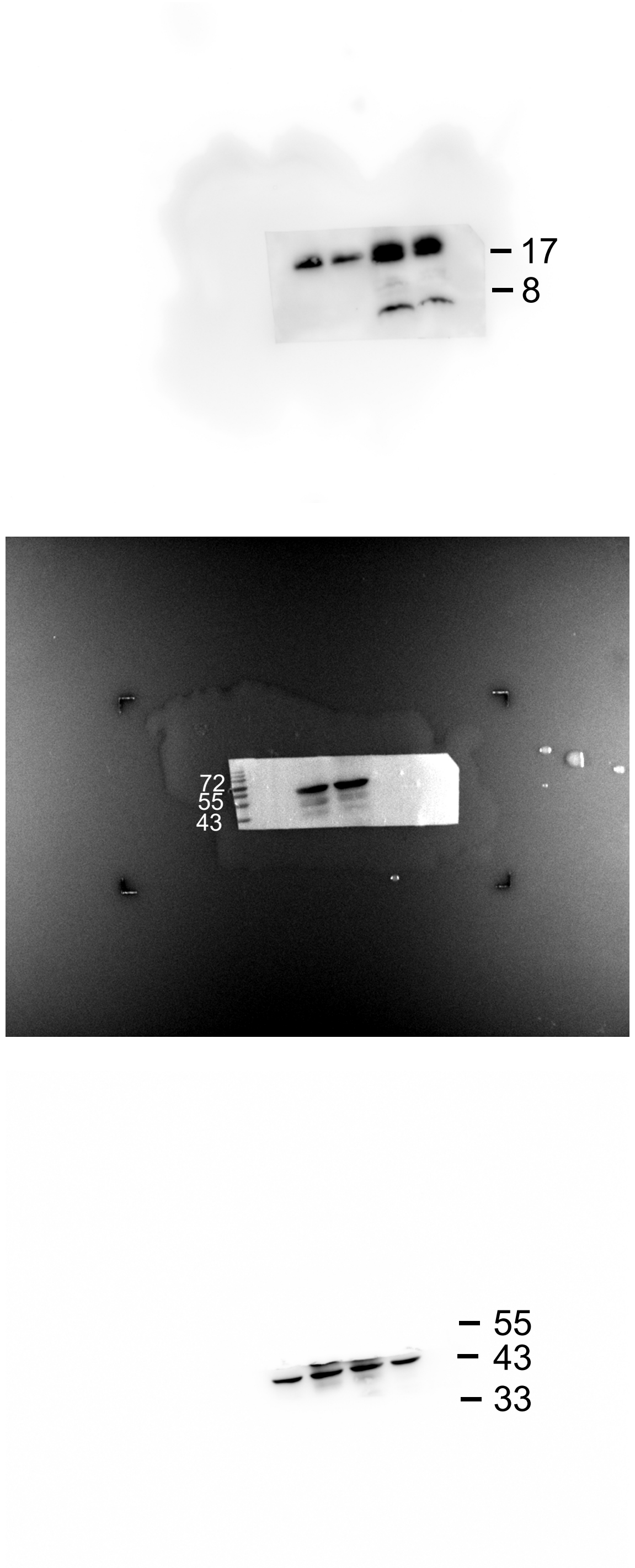

Figure 8C

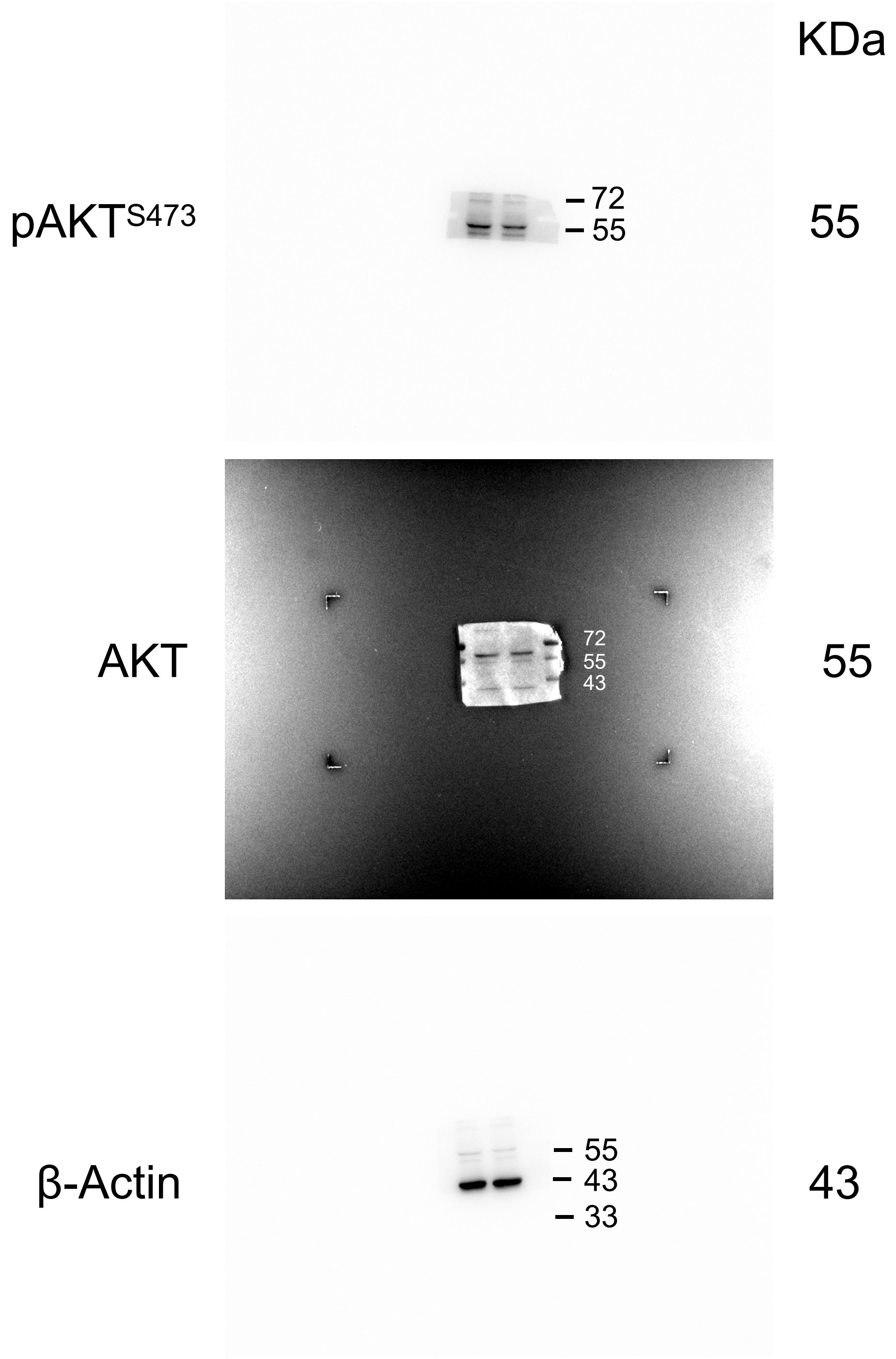

Figure 8D

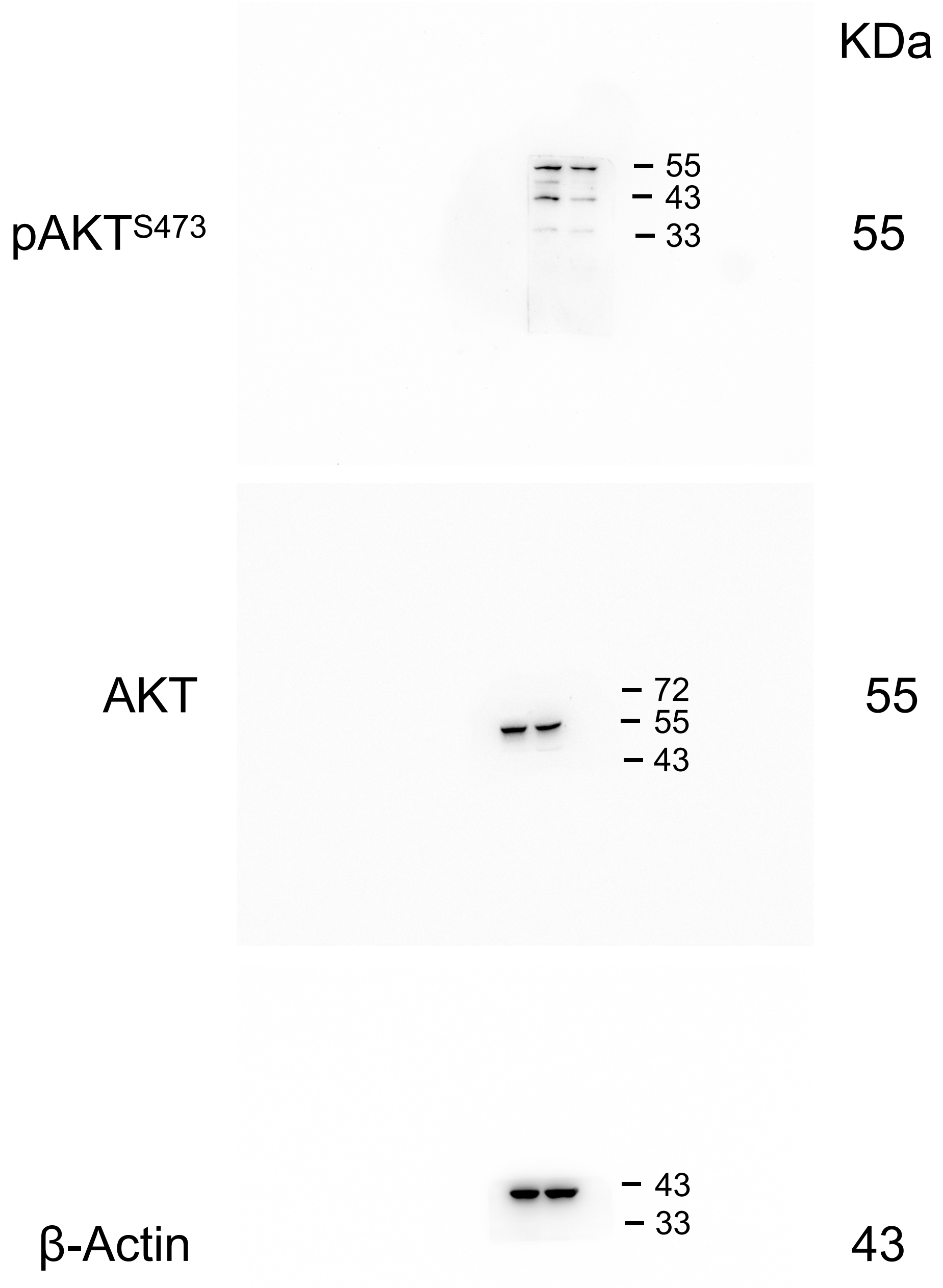

Figure 8E

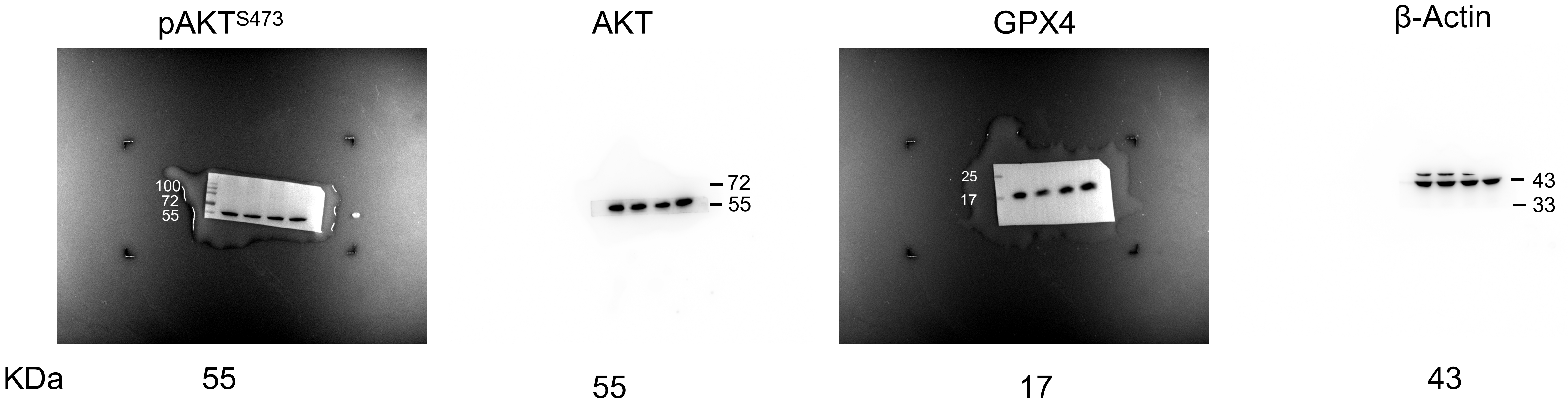
pAKT S473
AKT
GPX4
β-Actin
100
72
55
72
55
25
17
43
33
KDa
55
55
17
43

Figure 8F

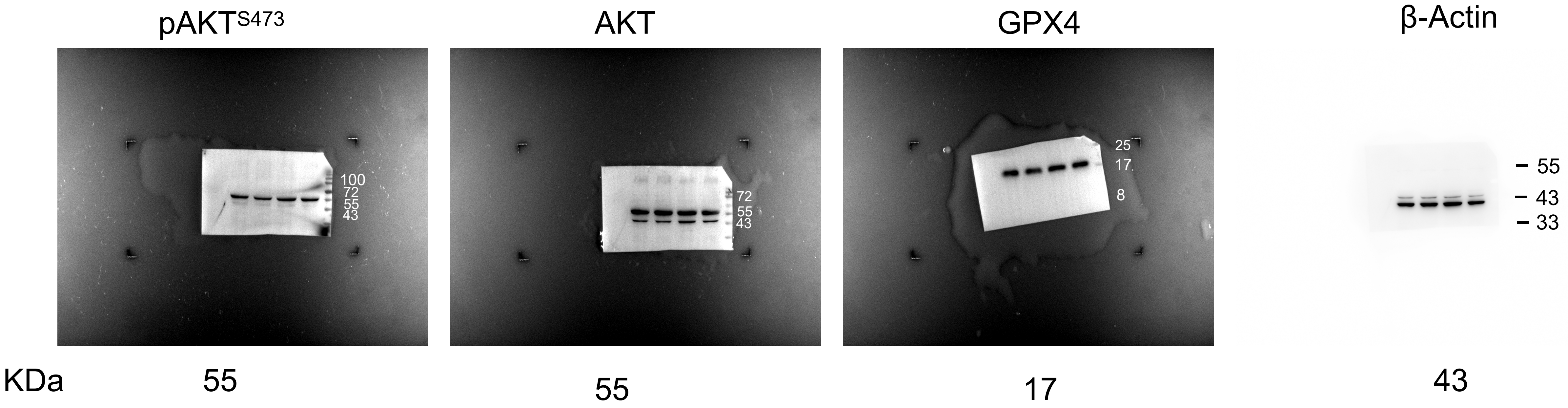
pAKT^{S473}
AKT
GPX4
β-Actin
100
72
55
43
72
55
43
25
17
8
55
43
33
KDa
55
55
17
43

Figure 8I

KDa

E-cadherin 130

TWIST1 37

β-Actin 43

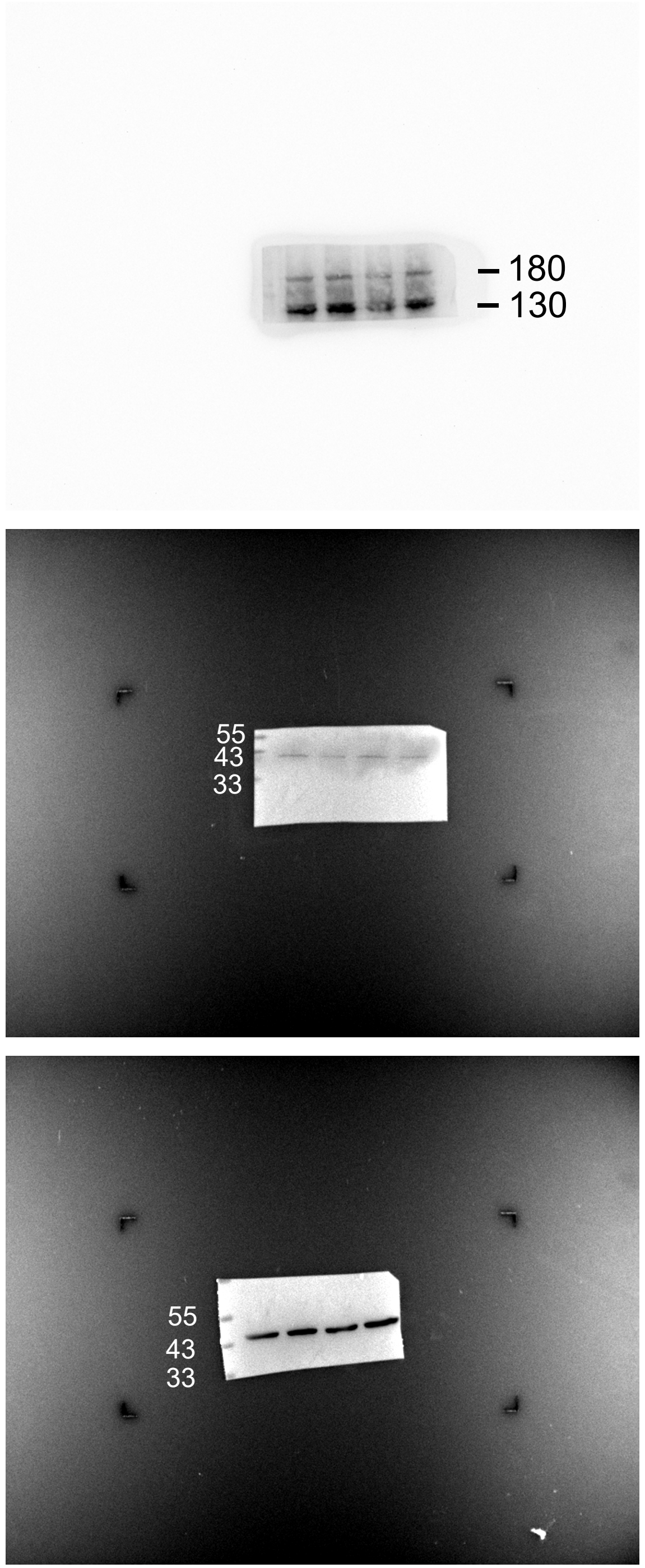

Figure 8K

KDa

E-cadherin 130

TWIST1 37

β-Actin 43

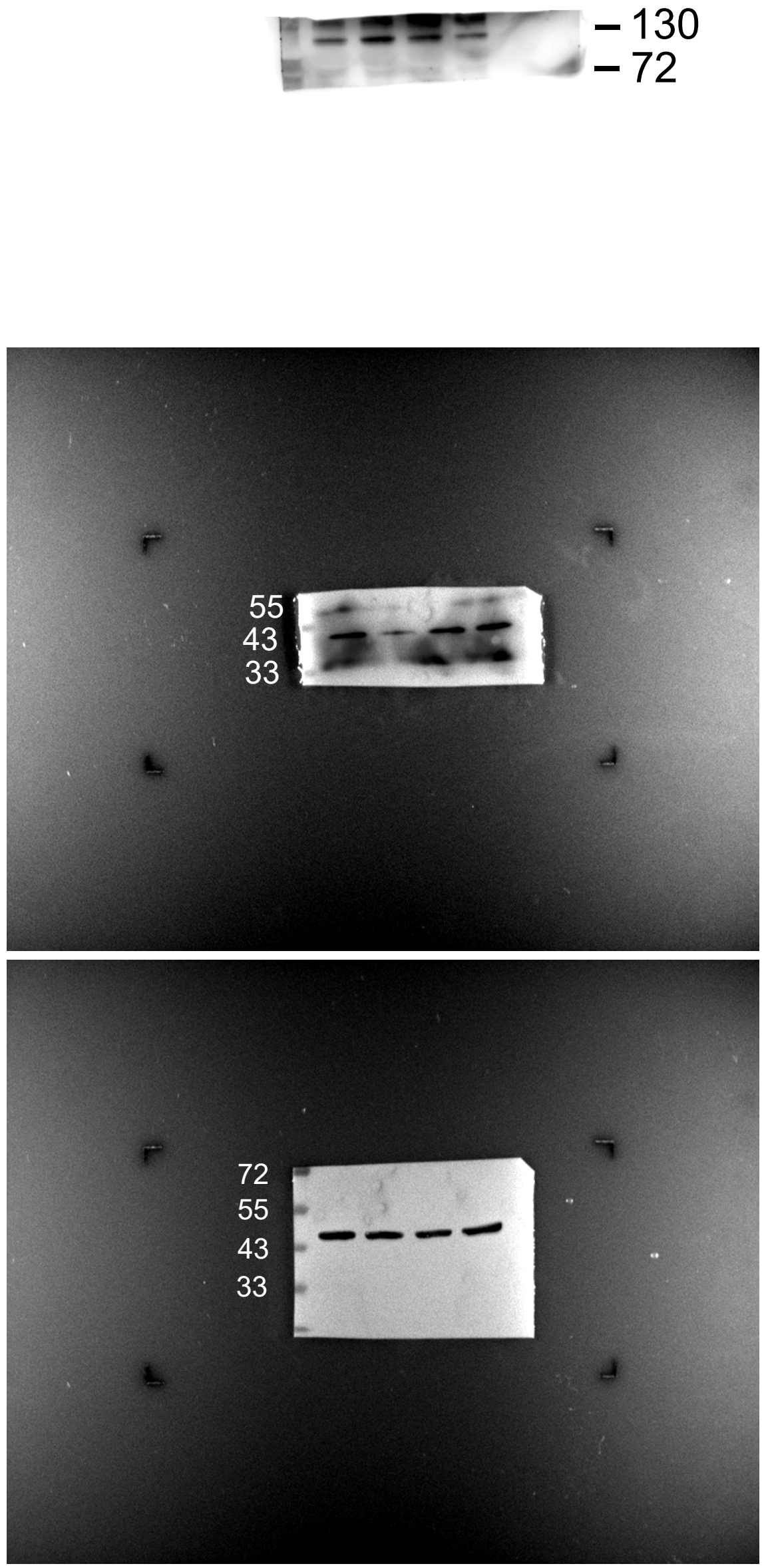

Figure S2 E

KDa

TFCP2L1 — 55

β-Actin — 43

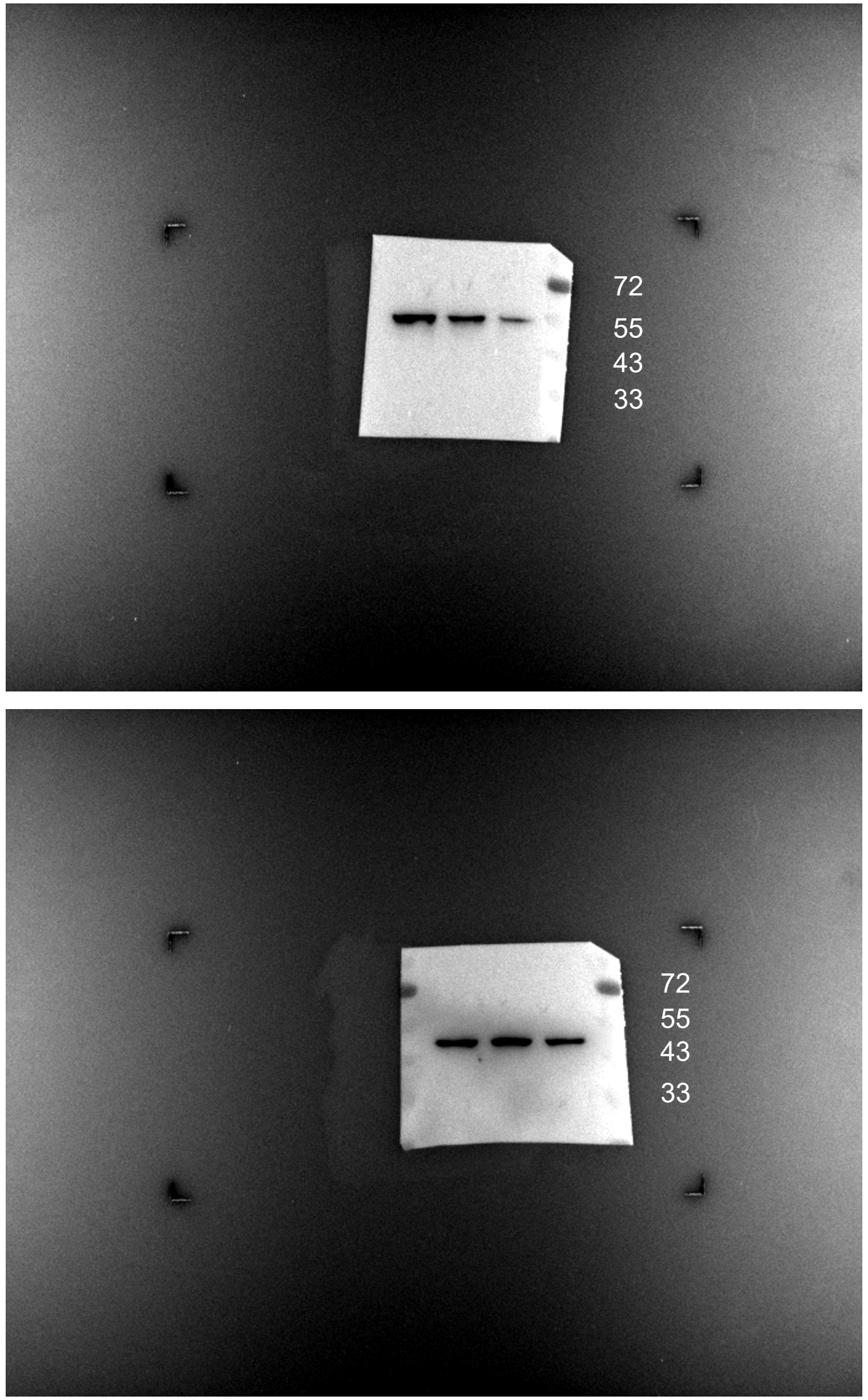

Figure S4 F

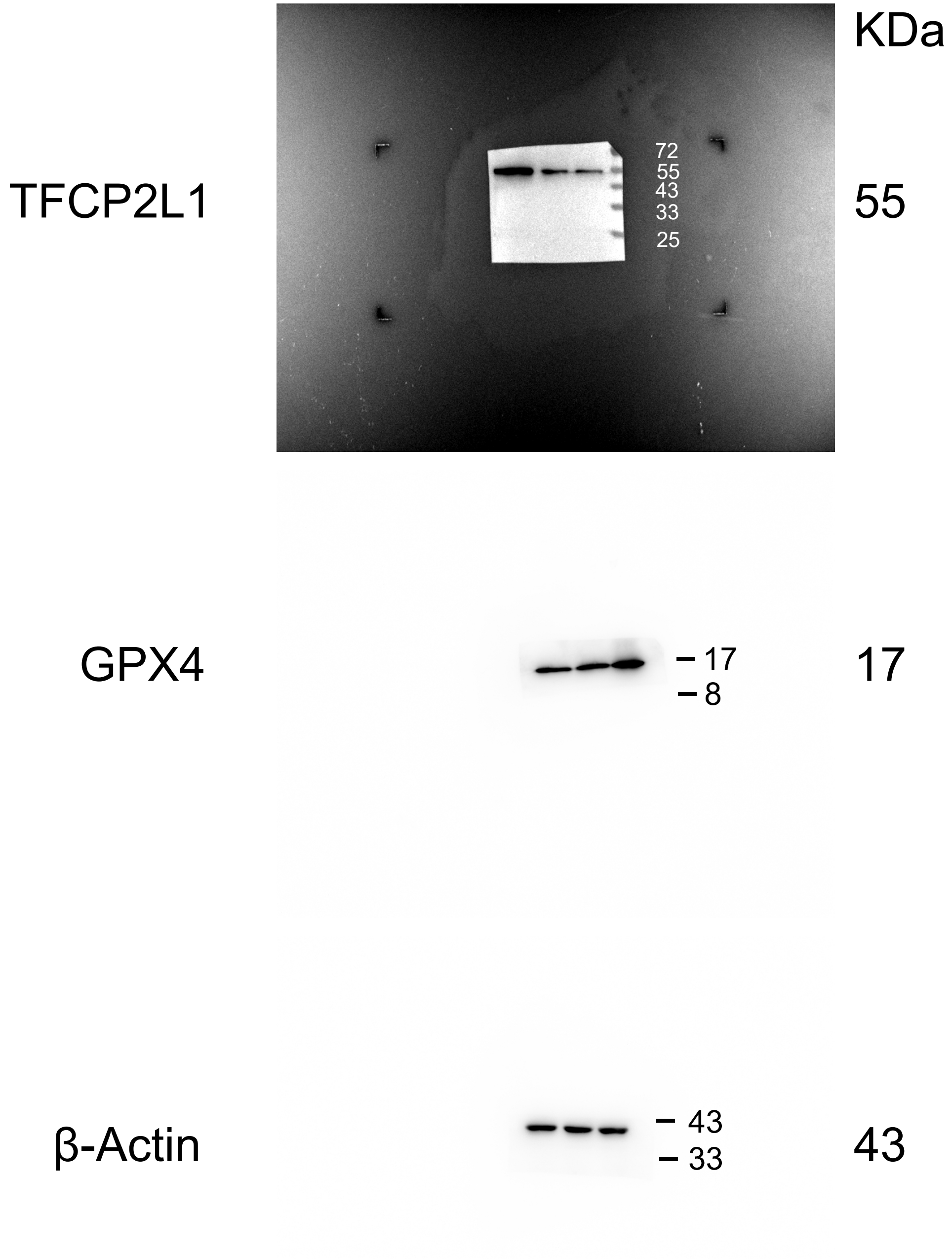

Figure S5 B

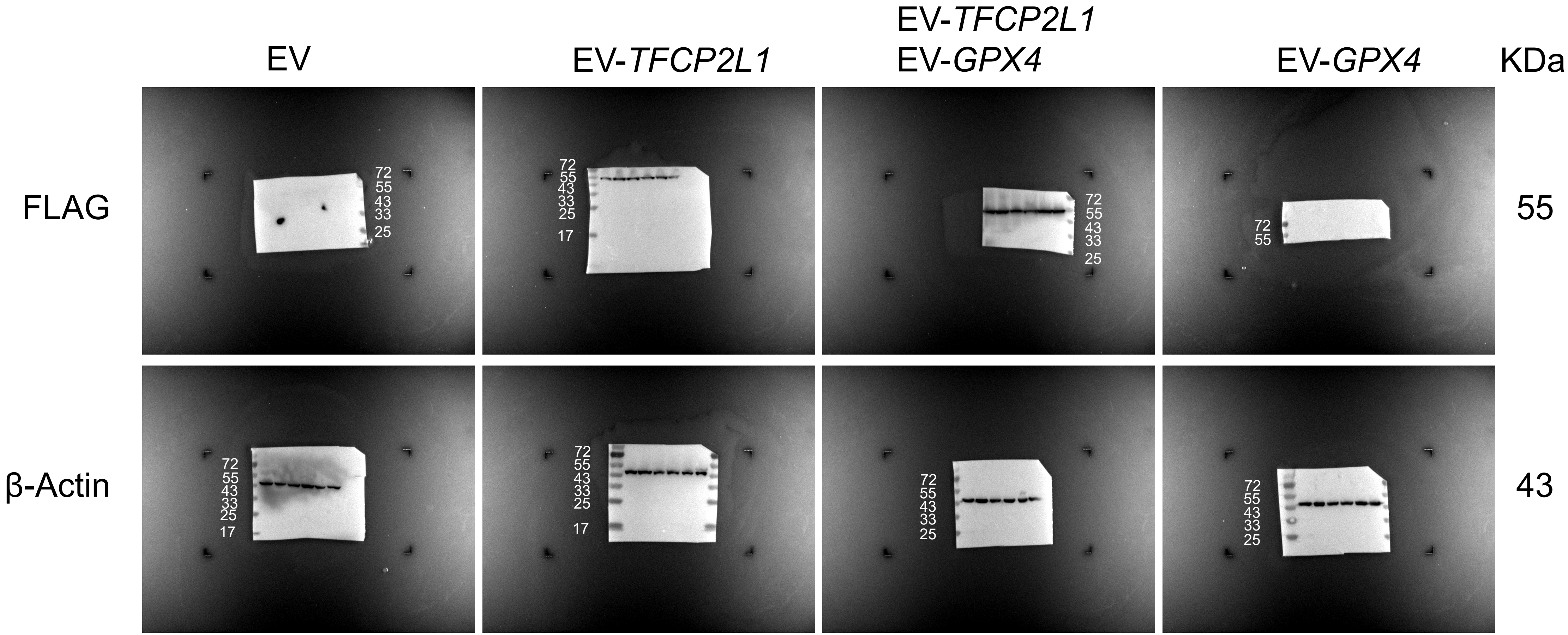
EV
EV-*TFCP2L1*
EV-*TFCP2L1*
EV-*GPX4*
EV-*GPX4*
KDa
FLAG
55
β-Actin
43
72
55
43
33
25
17

Figure S6 A

KDa

GPX4 17

FLAG 55

β-Actin 43

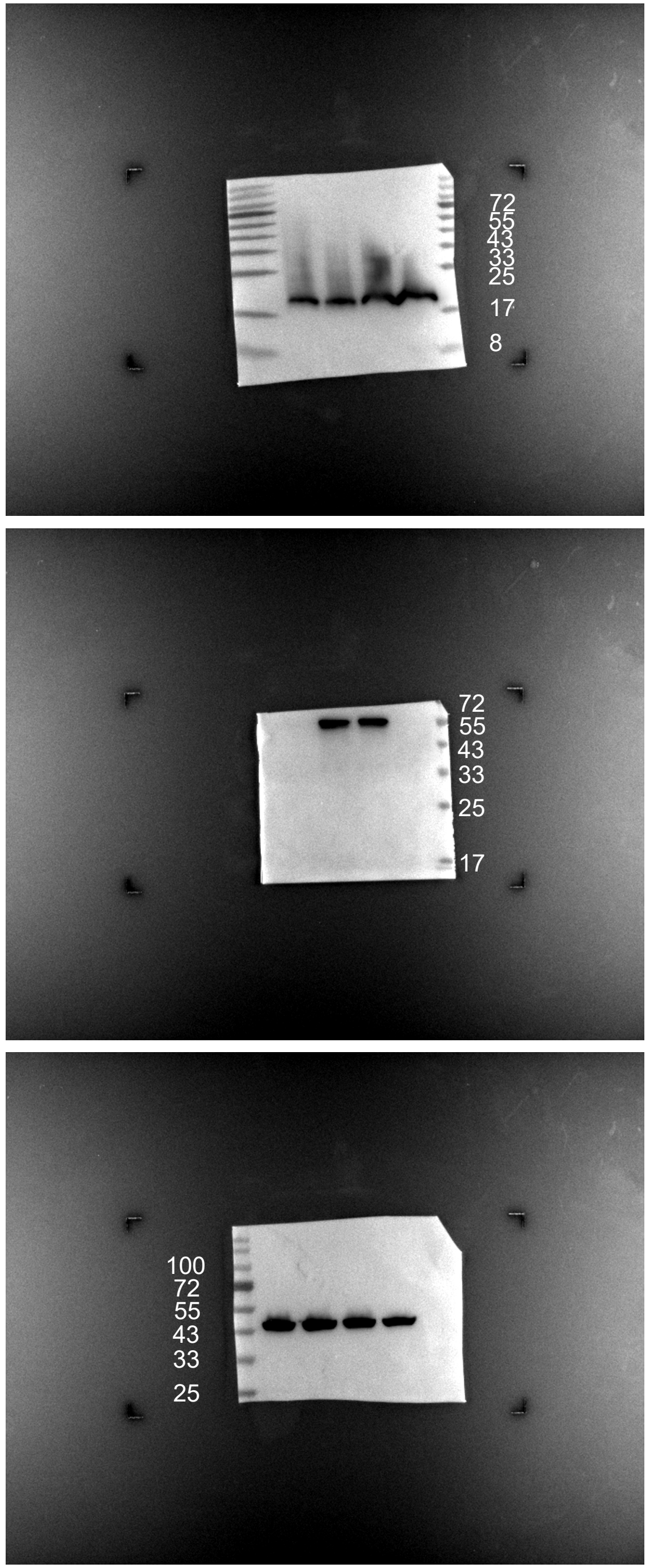

Figure S6 E

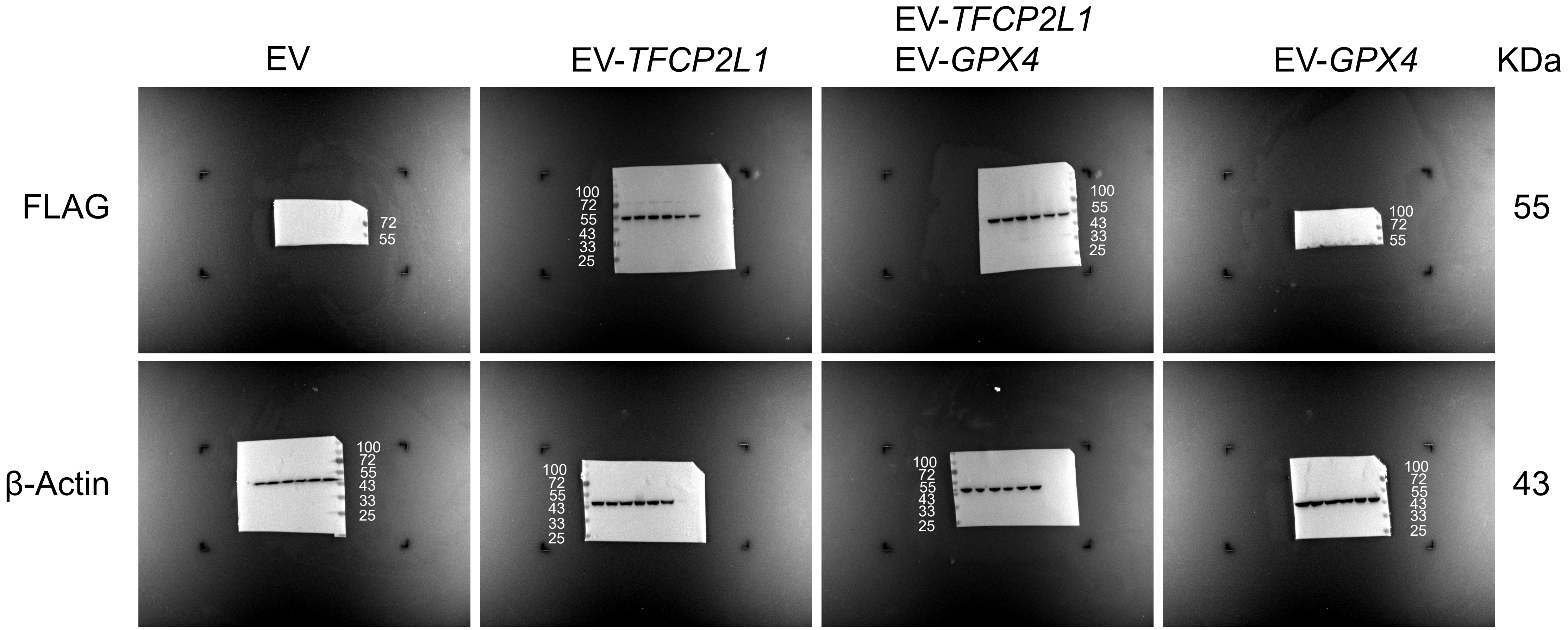
EV
EV-*TFCP2L1*
EV-*TFCP2L1*
EV-*GPX4*
EV-*GPX4*
KDa
FLAG
55
β-Actin
43
100
72
55
43
33
25

Figure S7 A

KDa

pAKT^{T308}

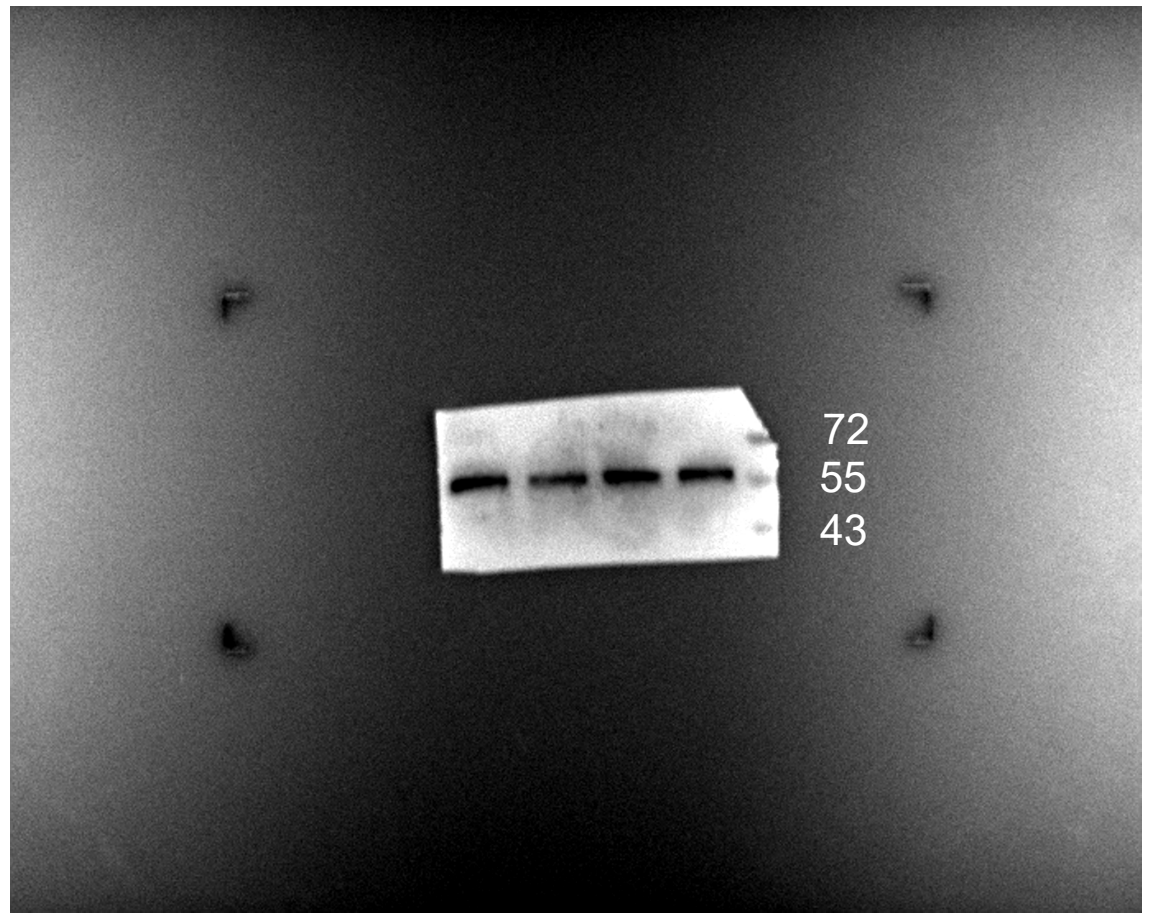

55

GPX4

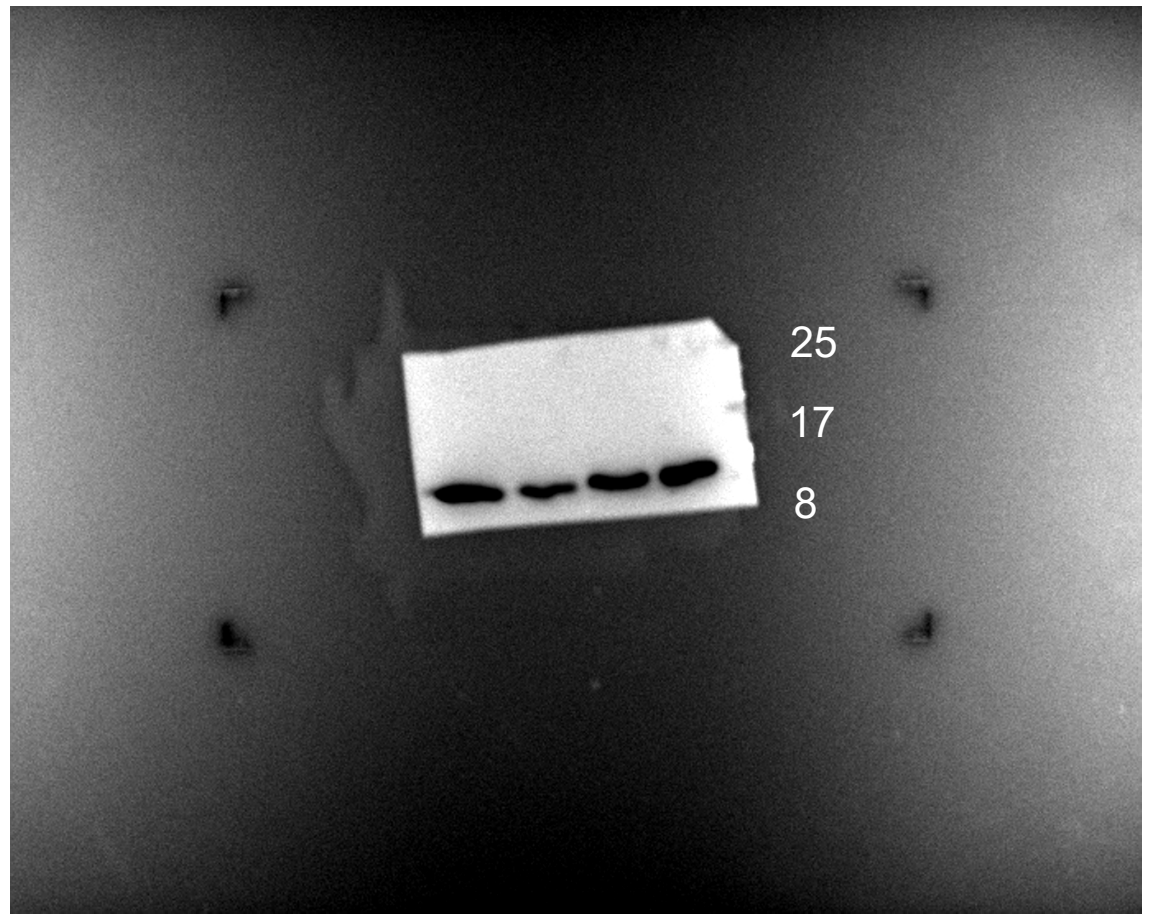

17

β-Actin

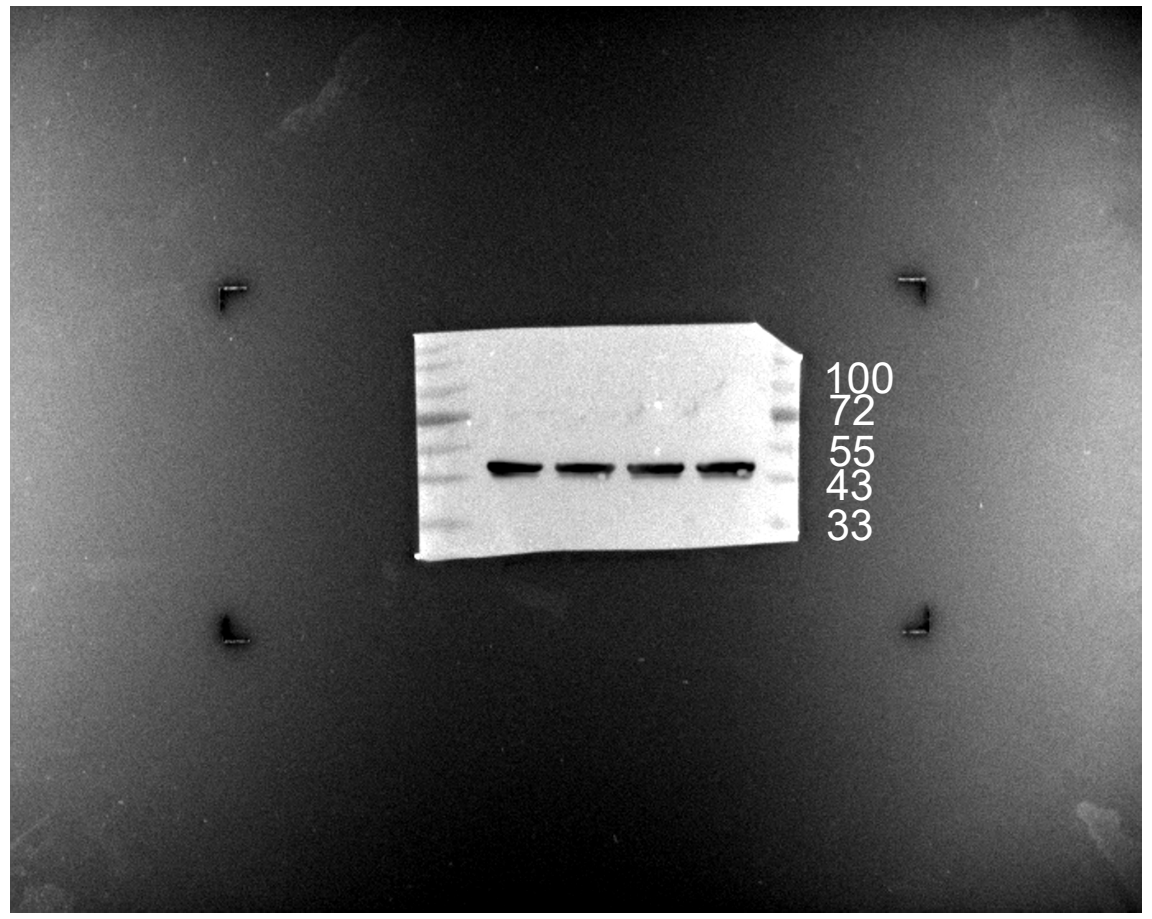

43

Figure S7 B

KDa

pAKT^{T308} 55

GPX4 17

β-Actin 43

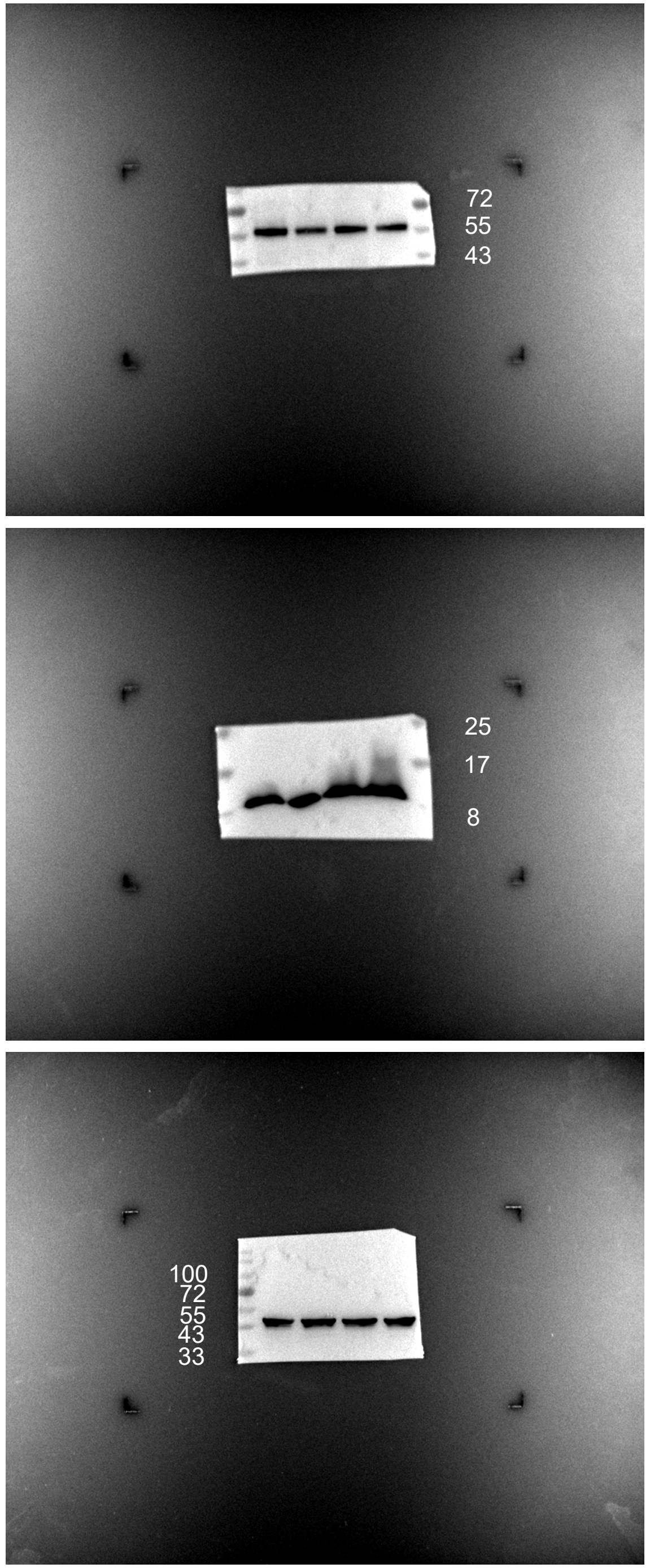

Supplement: Supplementary file 3 [file 41420_2026_3231_MOESM3_ESM.pdf]
